# Physiologically based radiopharmacokinetic (PBRPK) modeling to simulate and analyze radiopharmaceutical therapies: studies of non-linearities, multi-bolus injections, and albumin binding

**DOI:** 10.1186/s41181-023-00236-w

**Published:** 2024-01-22

**Authors:** Ali Fele-Paranj, Babak Saboury, Carlos Uribe, Arman Rahmim

**Affiliations:** 1 School of Biomedical Engineering, University of British Columbia, Vancouver, BC Canada; 2 Department of Integrative Oncology, BC Cancer Research Institute, Vancouver, BC Canada; 3 Department of Radiology and Imaging Sciences, Clinical Center, National Institutes of Health, Bethesda, Maryland US; 4 Department of Functional Imaging, BC Cancer, Vancouver, BC Canada; 5 Department of Radiology, University of British Columbia, Vancouver, BC Canada

**Keywords:** PBPK modeling, Radiopharmaceutical therapy, Theranostics, Computational modeling, Therapy optimization

## Abstract

**Background:**

We aimed to develop a publicly shared computational physiologically based pharmacokinetic (PBPK) model to reliably simulate and analyze radiopharmaceutical therapies (RPTs), including probing of hot-cold ligand competitions as well as alternative injection scenarios and drug designs, towards optimal therapies.

**Results:**

To handle the complexity of PBPK models (over 150 differential equations), a scalable modeling notation called the “reaction graph” is introduced, enabling easy inclusion of various interactions. We refer to this as physiologically based radiopharmacokinetic (PBRPK) modeling, fine-tuned specifically for radiopharmaceuticals. As three important applications, we used our PBRPK model to (1) study the effect of competition between hot and cold species on delivered doses to tumors and organs at risk. In addition, (2) we evaluated an alternative paradigm of utilizing multi-bolus injections in RPTs instead of prevalent single injections. Finally, (3) we used PBRPK modeling to study the impact of varying albumin-binding affinities by ligands, and the implications for RPTs. We found that competition between labeled and unlabeled ligands can lead to non-linear relations between injected activity and the delivered dose to a particular organ, in the sense that doubling the injected activity does not necessarily result in a doubled dose delivered to a particular organ (a false intuition from external beam radiotherapy). In addition, we observed that fractionating injections can lead to a higher payload of dose delivery to organs, though not a differential dose delivery to the tumor. By contrast, we found out that increased albumin-binding affinities of the injected ligands can lead to such a differential effect in delivering more doses to tumors, and this can be attributed to several factors that PBRPK modeling allows us to probe.

**Conclusions:**

Advanced computational PBRPK modeling enables simulation and analysis of a variety of intervention and drug design scenarios, towards more optimal delivery of RPTs.

**Supplementary Information:**

The online version contains supplementary material available at 10.1186/s41181-023-00236-w.

## Introduction

Radiopharmaceutical therapies (RPTs) involve the injection of molecules (ligands) labeled with radioisotopes to target specific binding sites with increased expression in tumors (Lever 2002; National Research Council 2007; Banerjee et al. 2015; Morris et al. 2021). The goal is to use radiopharmaceuticals to cause cellular damage instead of tracer amounts that do not perturb biological systems (Sgouros et al. 2020; Salih et al. 2022; Sgouros 2019; Lindsley et al. 2022; Sgouros 2023; Pallares and Abergel 2022). This vastly emerging paradigm has led to significant excitement, while there is a significant number of phenomena that need to be better understood towards optimal, personalized, and precision RPTs (Siebinga et al. 2022, 2021; Zaid et al. 2021; Rahmim et al. 2022; Kletting et al. 2016; Gospavic et al. 2016; Hardiansyah et al. 2016; Maaß et al. 2016).

In RPTs, dose delivery to the tumor and organs at risk (OARs) depends on a wide array of factors, e.g. the blood circulation in the cardiovascular system of the body (English et al. 2022). It is a challenging task to predict absorbed dose by tumors and OARs by solely knowing the injection parameters (i.e. injected activity and specific activity) (Divgi et al. 2021), and as a result, absorbed doses can span an order-of-magnitude (Del Prete et al. 2019; Violet et al. 2019), and as a result, patients are commonly undertreated. To provide a better understanding, physiologically based pharmacokinetics models fine-tuned to radiopharmaceuticals have been proposed (Siebinga et al. 2022).

There have been previous attempts in modeling the kinetics of pharmaceuticals, among which are ODE-based models (Zhang et al. 2022; Strand et al. 1993), PDE based models (Kiani Shahvandi et al. 2022), stochastic models (Convertino et al. 2018), etc. Many of these models were also fine-tuned to study the kinetics of radiopharmaceuticals (Kletting et al. 2009, 2012; Pfeifer et al. 2013; Hardiansyah et al. 2016; Gospavic et al. 2016; Hardiansyah et al. 2016; Maaß et al. 2016; Begum et al. 2018; Rinscheid et al. 2019; Bartelink et al. 2022). For instance, PBPK models utilize ordinary differential equations (ODE) to simulate the kinetics of pharmaceuticals/radiopharmaceuticals in the body right after the injection. These models include major organs in the body and treat their structures as different compartments while the flow of species from one compartment to the other is governed by ODEs.

PBPK model structures have been commonly designed in a way that can challenge scalability. To be more specific, previous models utilized the concept of “parallel” tracks to keep track of different species in the body; e.g. labeled and unlabeled radiopharmaceuticals. For instance, adding the concept of albumin interaction to the model (a new attempt pursued in our work) would require adding several other “parallel tracks” to the model which can make it very hard and complicated to implement. Lack of shared models in a standardized format, e.g., in systems biology markup language (SBML), can also challenge reproducible research into the kinetics of radiopharmaceuticals.

In this work, we provide a publicly-shared upgraded design of PBPK modeling that is easily scalable. We studied the effect of the following factors on the absorbed dose by tumor and OARs: the competition between labeled and unlabeled ligands in binding to the binding sites, multi-bolus injection instead of a single bolus injection, and the strength of ligand-albumin affinity.

## Methods

We used the PBPK model structure developed by Kletting et al. (2016) as a baseline model for the kinetics of ^177^Lu-PSMA. This involves a compartmental modeling approach used to simulate the distribution of radiopharmaceuticals among different organs in the body. Each organ is treated as a collection of compartments, each associated with a different spacial structure of the organs (i.e. vascular structure, interstitial space, PSMA binding sites, and the internal space of cells). The interchange of material between these compartments is governed by ordinary differential equations (ODE). However, since there are two types of pharmaceuticals circulating the body, labeled and unlabeled, two parallel tracks need to be implemented to take these two types into account (see the Additional file 1: supplementary file subsections S.1 and S.2 for more details about the model).

We expanded the model and made it more scalable to enable more detailed interactions of the radiopharmaceuticals with other species (i.e. albumin). We changed the definition of a compartment (as used in Kletting et al. (2016)) from a container containing only one variable to a container that can contain several variables called species, while species have their own interaction with each other (through the reaction graph as discussed in Additional file 1). This modeling structure eliminates the need for the “parallel” track to take the different pharmaceutical types into account. The concept of parallel tracks is not scalable if we were to explore the effect of albumin binding with more biological details (for instance to consider the fact that albumin can leak to the interstitial space of a tumor but not to the interstitial space of the OARs).

We implemented the model in MATLAB Simbiology and utilized population-measured values (see the Additional file 1: supplementary file section S.4) for the model parameters (as used in Kletting et al. 2016). Different variations of some parameters (e.g. different tumor volumes, tumor receptor densities, etc.) constitute the “virtual patients”. The data published in Kletting et al. (2016) were used for model validation. Implementation of this work is shared publicly in SBML format to ease model reproduction (see model sharing in Sect. ).

The PBRPK model is used to calculate the time activity curves for different organs under different simulation settings (as discussed below). Subsequently, we used the dosimetry methods, as discussed in Kletting et al. (2016) and section S.3 in the Additional file 1: supplementary file, to calculate the absorbed doses for further analysis.

### Hot and cold interaction

We injected virtual patients (differing in the tumor receptor densities and tumor volumes) with different combinations of labeled and unlabeled ligands (see Table 1) while measuring the dose delivered to the tumor and OAR.

**Table 1:** The range of parameter values on injected hot, injected cold, tumor receptor density, and tumor volume to study the competition between hot and cold species

Parameter	Range of values	Unit
Injected hot amount	10 linear samples from the interval [5,100]	nmol
Injected cold amount	10 linear samples from the interval [25,800]	nmol
Tumor receptor density	10 linear samples from the interval [10,890]	nmol/lit
Tumor volume	10 linear samples from the interval [40,2100]	ml

**Fig. 1:**
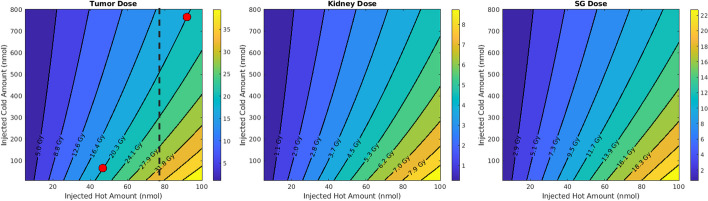
Dependence of the delivered dose to organs on the injected hot amount and injected cold amount. Moving from the left side of each plot to the right side, we observe that the iso-dose lines get tilted, suggesting more competition between hot and cold species. In addition, the two dots on the 20 Gy iso-dose curve reveal that one can achieve the same absorbed dose in the tumor by lower injected activity if accompanied by lower cold amount (i.e. higher specific activity)

Furthermore, to be able to compare the effect of different combinations of labeled and unlabeled radiopharmaceuticals under different patient settings (i.e. different tumor volume and tumor receptor density), we developed the concept of twist, which is a geometrical measure of the tiltedness of the iso-dose curves (see Fig. 1). In fact, twist is measuring the angle between a straight line fitted to each iso-dose curve with the straight line fitted to the iso-dose curve corresponding to the lowest dose value.

### Injection profile

As any multi-bolus injections (with the same injected and specific activity) can be characterized by two parameters, number of injections and time between each injection), we performed a parameter sweep on the Cartesian product of the different values for each of them, while keeping the total injected labeled and unlabeled ligands to be 10 nmol and 100 nmol respectively. It is worth noting that, for instance, a fractionated injection consisting of 5 injections with 100 minutes between each, is assumed to be all administrated in a single therapy session. As such, in this work, instead of injecting the entire portion at once, we explored the effects of injections in smaller portions, as characterized by the parameters “time between injections” and “number of injections” (see Table 2). Furthermore, to consider patient heterogeneities, we tested different injection strategies on virtual patients with different tumor receptor densities and tumor volumes of reasonable values (see Table 2).

**Table 2:** The range of parameter values utilized for evaluating the effect of injection profile (multi-bolus injection)

Parameter	Range of values	Unit
Number of injections	{1,2,3,4,5,6,7,8,9,10}	Dimensionless
Time between injections	{10,50,100,200,300,400,500,750,1000}	min
Tumor receptor density	10 linear samples from the interval [43,342]	nmol/lit
Tumor volume	10 linear samples from the interval [40,2100]	ml
Injection coefficient	{1,2,3,4,5,6}	Dimensionless

To quantify the effect of multi-bolus injection in contrast to the single bolus injection, we found the injection strategy yielding the highest delivered dose to each organ and tumor, and then calculated the relative change in dose compared to the bolus injection (we call it the maximum relative dose change or MRDC). Note that since dose and TIA are different up to a multiplicative constant, the maximum relative dose change is the same as the maximum relative TIA change. In short, MRDC can be defined as:

$$\begin{aligned} \text {MRDC} = \frac{\text {Dose}_i(n^*, \tau ^*) - \text {Dose}_i(0,0)}{\text {Dose}_i(0,0)}, \end{aligned}$$

in which $\text {Dose}_i(n,\tau )$ is the dose delivered to organ *i* as a function of number of injections (*n* indexed from 0 upwards) and time between injections ($\tau$), $n^*$ and $\tau ^*$ are the values which maximizes the dose to organ *i*. Note that $\text {Dose}_i(0,0)$ means the dose delivered to organ *i* under a single bolus injection.

### Albumin

For this study, we performed a parameter sweep on the different values of $K_D^{alb}$, logarithmically sampled from the range [5, $10^6$] nmol/lit (see Table 3). For each of those simulations, we injected the patients with 10 nmol hot ligands and 100 nmol cold ligands.

For each value of $K_D$, we calculated the blood residence time (time-integrated activity in the vein compartment divided by the injected activity) as well as the delivered dose to the organs under study. The blood residence time (BRT) can be written as

$$\begin{aligned} BRT = \frac{\int _{0}^{t_{end}} A_{vein}(\tau ) d\tau }{A_0} \end{aligned}$$

in which $A_{vein}(\tau )$ is the activity in the vein compartment which is simply $A_{vein}(\tau ) = \lambda _{phys} H_{vein}$ with $H_{vein}$ representing the hot ligands in the vein, and $A_{0}$ is the injected activity that is simply $A_0 = \lambda _{phys} H_0$ with $H_0$ representing the injected hot amount.

**Table 3:** The range of parameter values used to study the effect of albumin binding on the kinetics of radiopharmaceuticals

Parameter	Range of values	Unit
$K_D^{alb}$	20 logarithmically spaced values from [5, $10^5$]	nmol/lit

To reflect the seek for the optimal therapy according to the main objective (i.e. increase the absorbed dose to the tumor while decreasing the delivered dose to OAR), we calculate a quantity called ”enhancement factor“ using the following formula

$$\begin{aligned} EF(K_D^{alb}) = \frac{\text {TumorDose}(K_D^{alb})/ \text {OrganDose}(K_D^{alb}) }{\text {TumorDose}(\infty ) / \text {OrganDose}(\infty )}, \end{aligned}$$

in which *EF* is the enhancement factor, $\text {TumorDose}(K_D^{alb})$ represented the tumor arborbed dose as a function of $K_D^{alb}$, and similarly $\text {OARDose}(K_D^{alb})$ represents the dose to organ at risk. Intuitively speaking, *EF* quantifies the effectiveness of albumin binding in delivering differential dose to tumor (relative to OAR). For instance, for a given value of $K_D^{alb}$, $EF= 4$ means that enabling albumin binding with the given $K_D^{alb}$, delivered 4 times more dose to the tumor relative to OAR, compared to the situation where albumin binding was off (i.e. $K_D^{alb} = \infty$).

## Results

Our scalable model implementation in Simbiology Matlab has a run time of $0.15 \pm 0.2$ seconds to generate the time activity curves for more than 18 organs for about 50,000 minutes. For instance, a parameter sweep consisting of 1000 model runs, would take somewhere around 150 seconds to complete. However, this can be accelerated with the parallel processing utility in Matlab and the run time can be reduced to about 90 seconds. The implemented model is shared in SBML format and can be found here (link to the GitHub repository). In what follows, we show the results of applying our model to the three above-mentioned studies, with further elaborations in the discussion section.

### Hot and cold interaction

In Fig. 1 we observe that for a fixed amount of injected activity (i.e. fixed hot amount), increasing the number of cold molecules (i.e. decreasing specific activities, and moving up on the dashed line in Fig. 1), will result in lower delivered doses to the tumor. Furthermore, the figure shows that moving from the iso-dose curve corresponding to the lowest dose (the leftmost) to the iso-dose curve with the highest dose value (the rightmost), the curves become more horizontal.

**Fig. 2:**
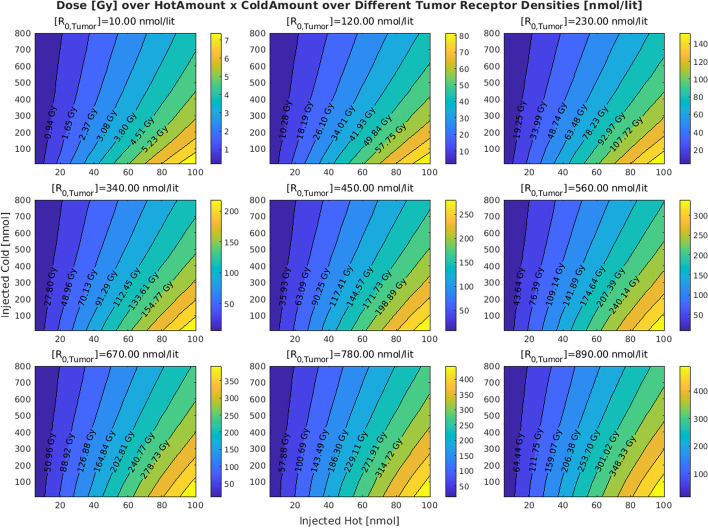
The effect of the tumor receptor density on the competition between hot and cold species for 20 ml for the tumor volume

**Fig. 3:**
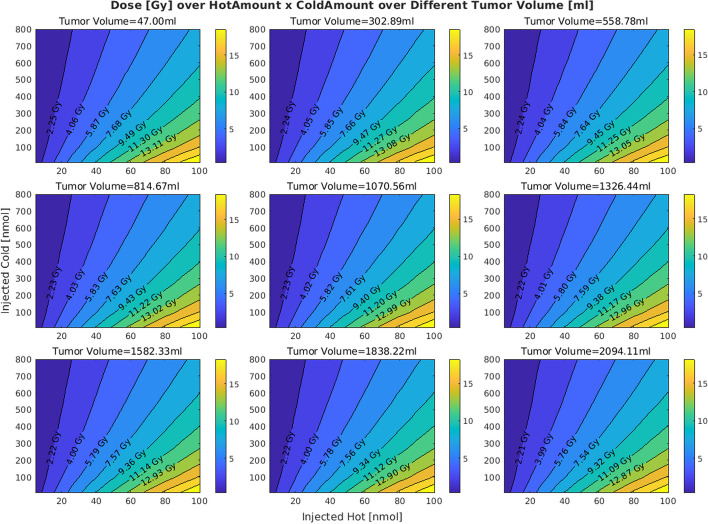
The effect of the tumor volume on the competition between hot and cold species. The value of tumor receptor density is set to 40 nmol/lit for this simulation

**Fig. 4:**
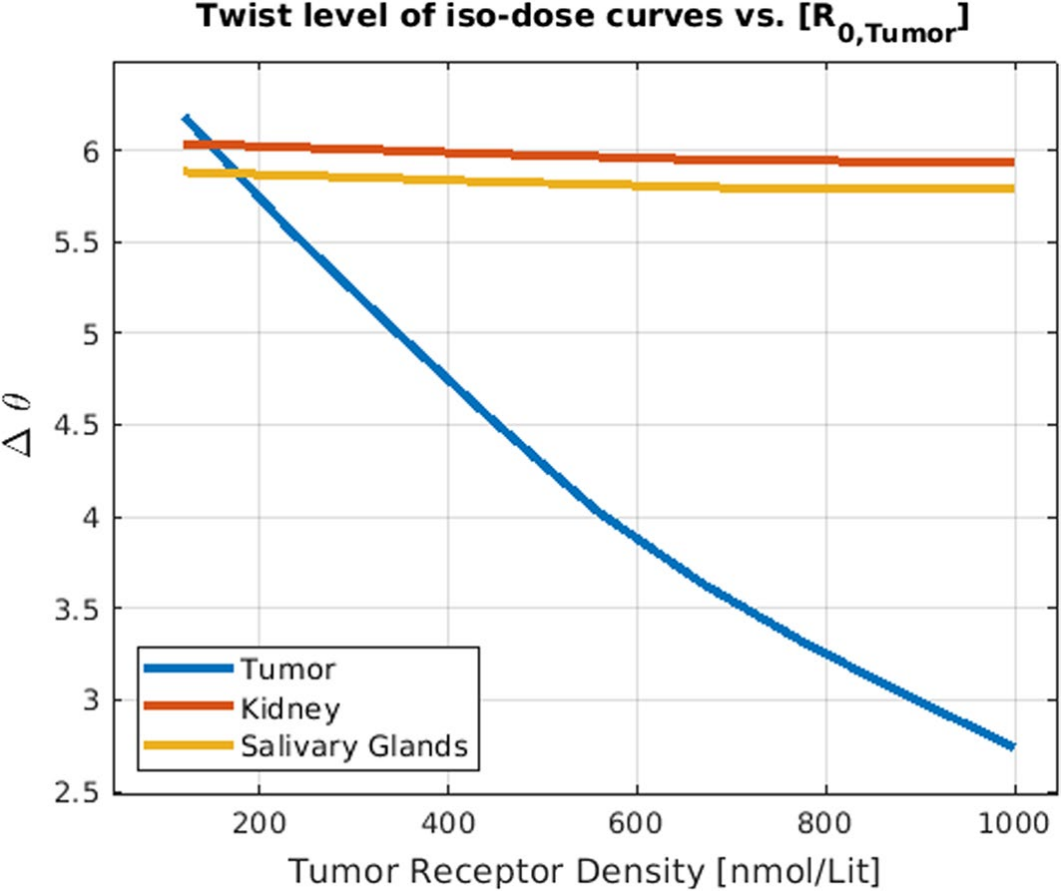
The effect of tumor receptor density on the competition degree (i.e. twist) between hot and cold species (with 20 ml for the tumor volume). Higher tumor receptor leads to less competition (thus less twist) in the tumor, while the competition level remains the same in kidney and salivary glands

**Fig. 5:**
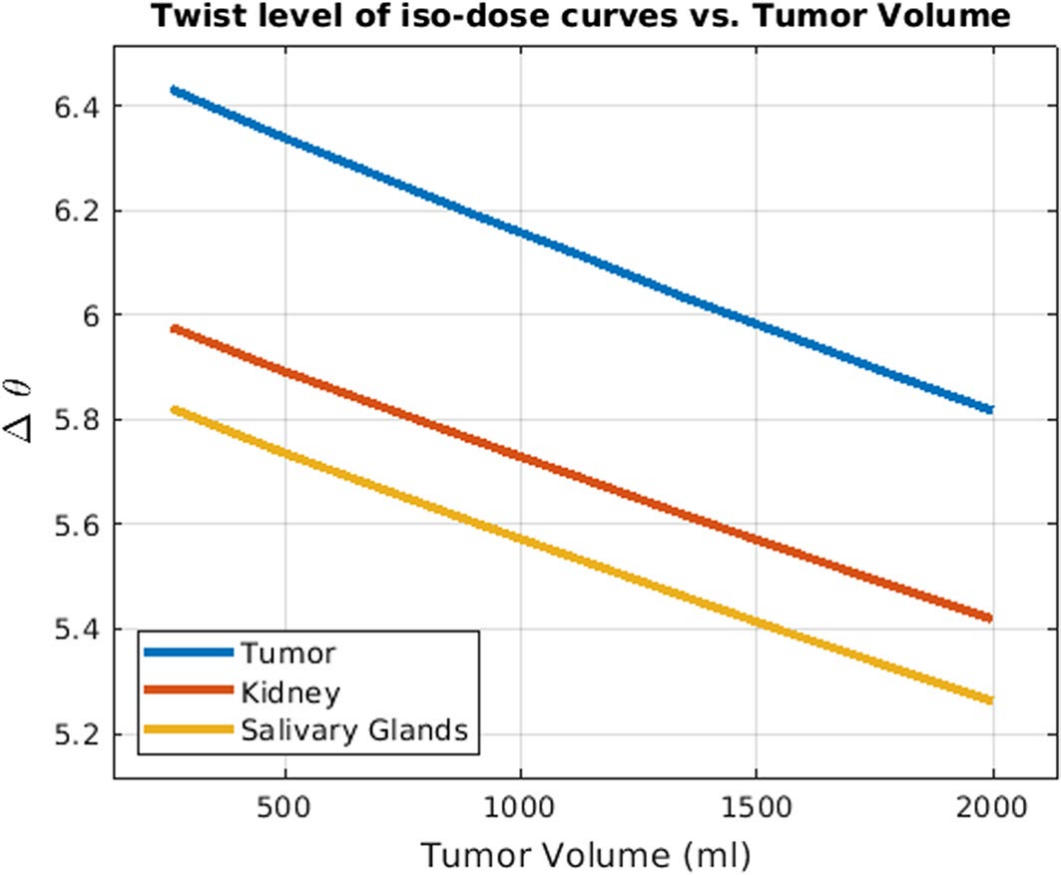
The effect of the tumor volume on the competition degree (i.e. twist) between hot and cold species (tumor receptor density set to 40 nmol/lit). Higher tumor volume does not alter competition in tumor as much as Fig. 4 (changing tumor receptor density), but more greatly impacts competition in kidney and salivary glands due to the tumor sink effect

Figures 2 and  3 show the effects of varying tumor receptor densities and tumor volumes on the tumor absorbed doses, respectively. It is observed that by increasing the tumor receptor density, the average delivered dose to the tumor increases. Furthermore, in doing so, the iso-dose curves become less tilted (horizontal) as we move from the iso-dose curve with the lowest dose value to the one with the highest dose value. This translates to the decreasing “twist” value as shown in Fig. 4. By contrast, when changing volumes, as shown in Fig. 5, this twist is less for tumors, but more for kidney and salivary glands (due to tumor sink effect). Overall, changing tumor receptors and volumes depict notable patterns.

### Injection profile

**Fig. 6:**
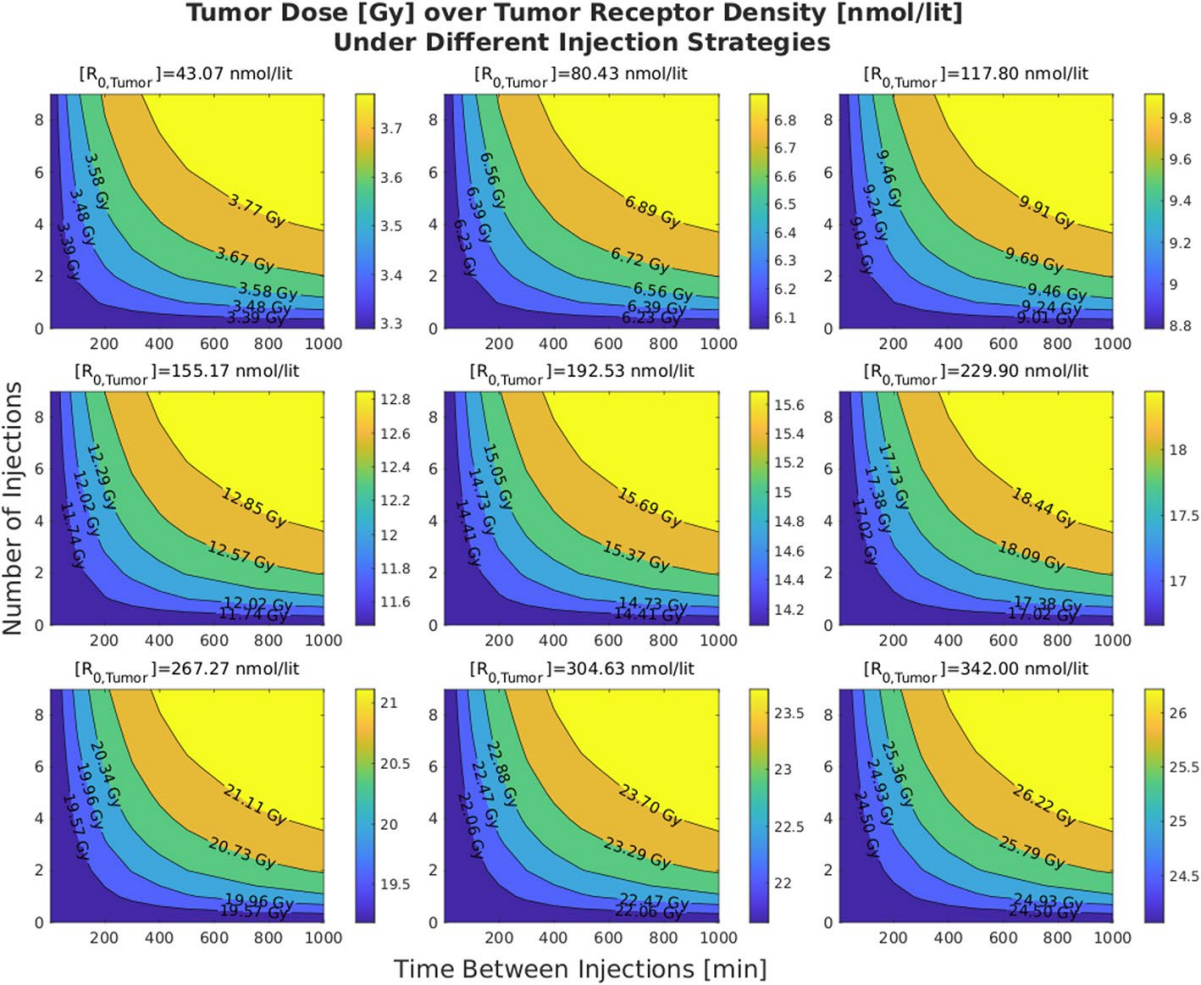
Effect of receptor density on the effectiveness of multi-bolus injection. These plots suggest that receptor density plays the main role in increasing the uptake due to injection fractionation

**Fig. 7:**
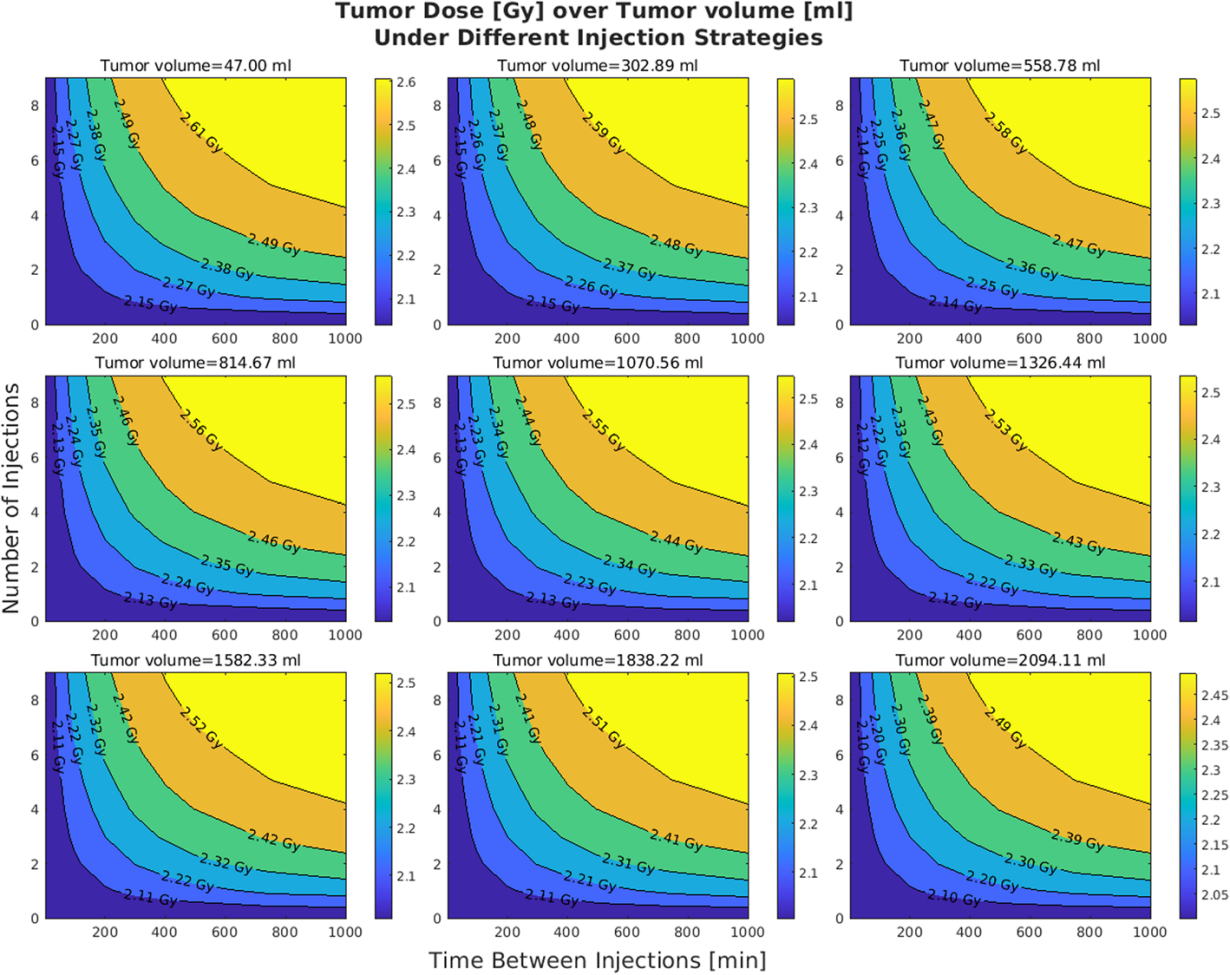
Effect of tumor volume on the effectiveness of multi-bolus injection. The value of tumor receptor density is set to 40 nmol/lit for these plots

Figures 6 and 7 shows the effect of multi-bolus injection on the delivered dose to the tumor, under different scenarios in which we consider different tumor volumes and tumor receptor densities. These figures show that under a fixed tumor volume or tumor receptor density, more fractionation (higher number of injections or higher time between injections) translates to a higher delivered dose to the tumor. Also, Fig. 6 especially shows the trend that with higher tumor receptor density the dose to tumor increases systematically (look at the color bar).

**Fig. 8:**
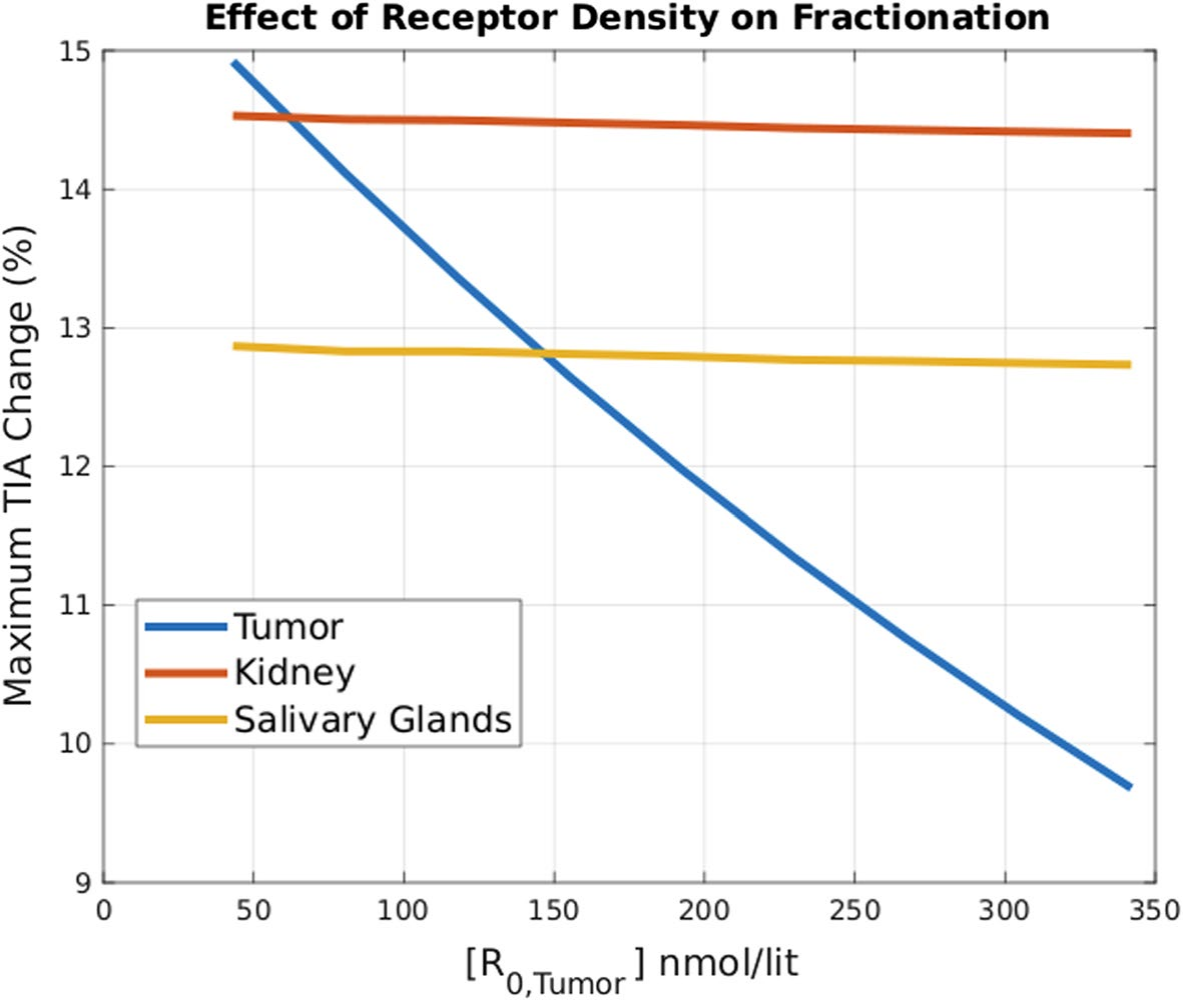
Effect of receptor density on injection fractionation (MRDC). We can observe that at low receptor densities, the effect of fractionation is more significant

However, these trends can be captured more clearly by calculating MRDC. For instance Fig. 8 shows that by fractionating the injection, the relative delivered dose to the tumor (compared to the baseline bolus injection) decreases with higher tumor receptor density.

### Albumin

**Fig. 9:**
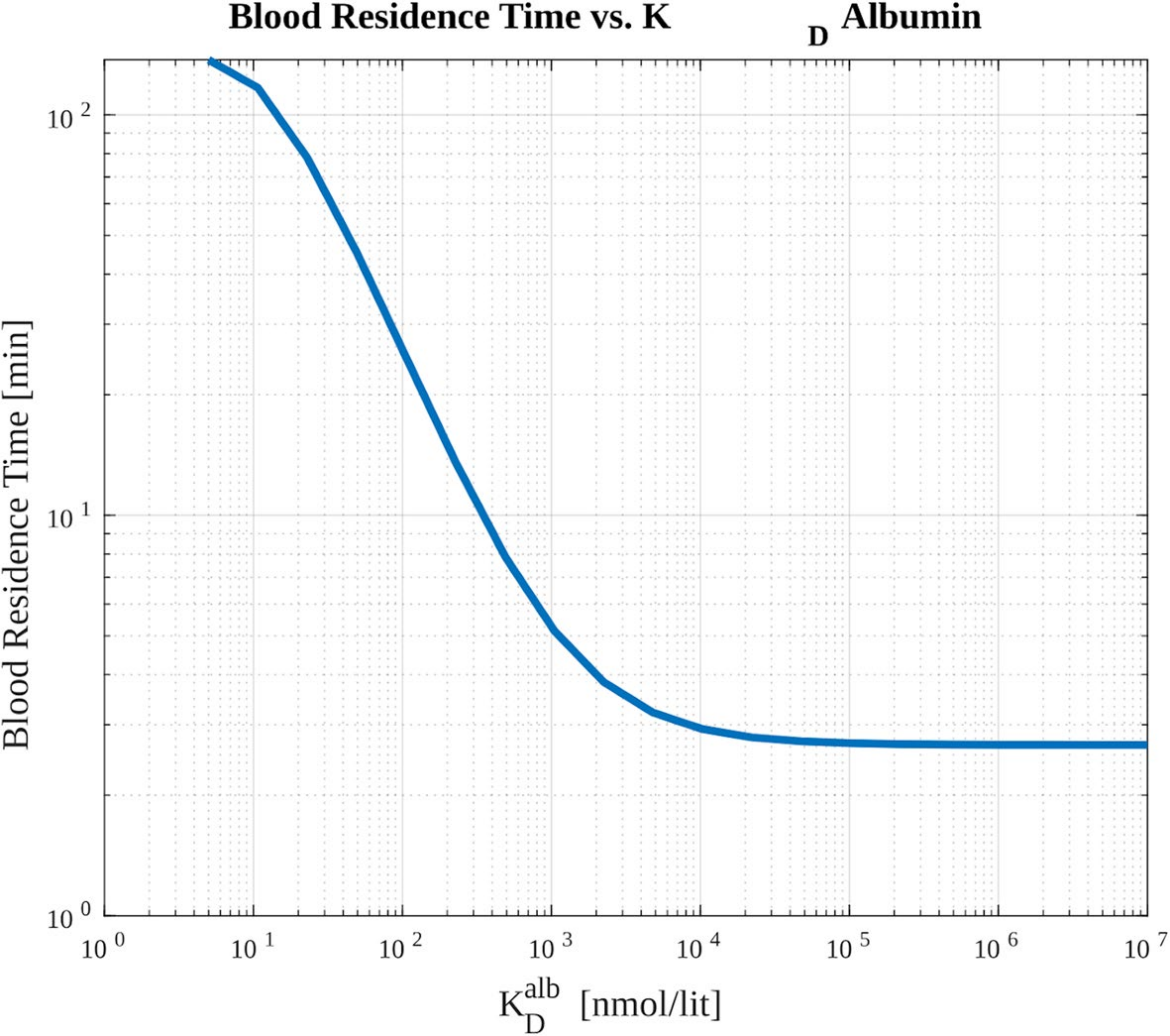
The effect of albumin affinity on the blood residence time of activity. We can observe that the increased albumin affinity (lower $K_D$) will increase the blood residence time

**Fig. 10:**
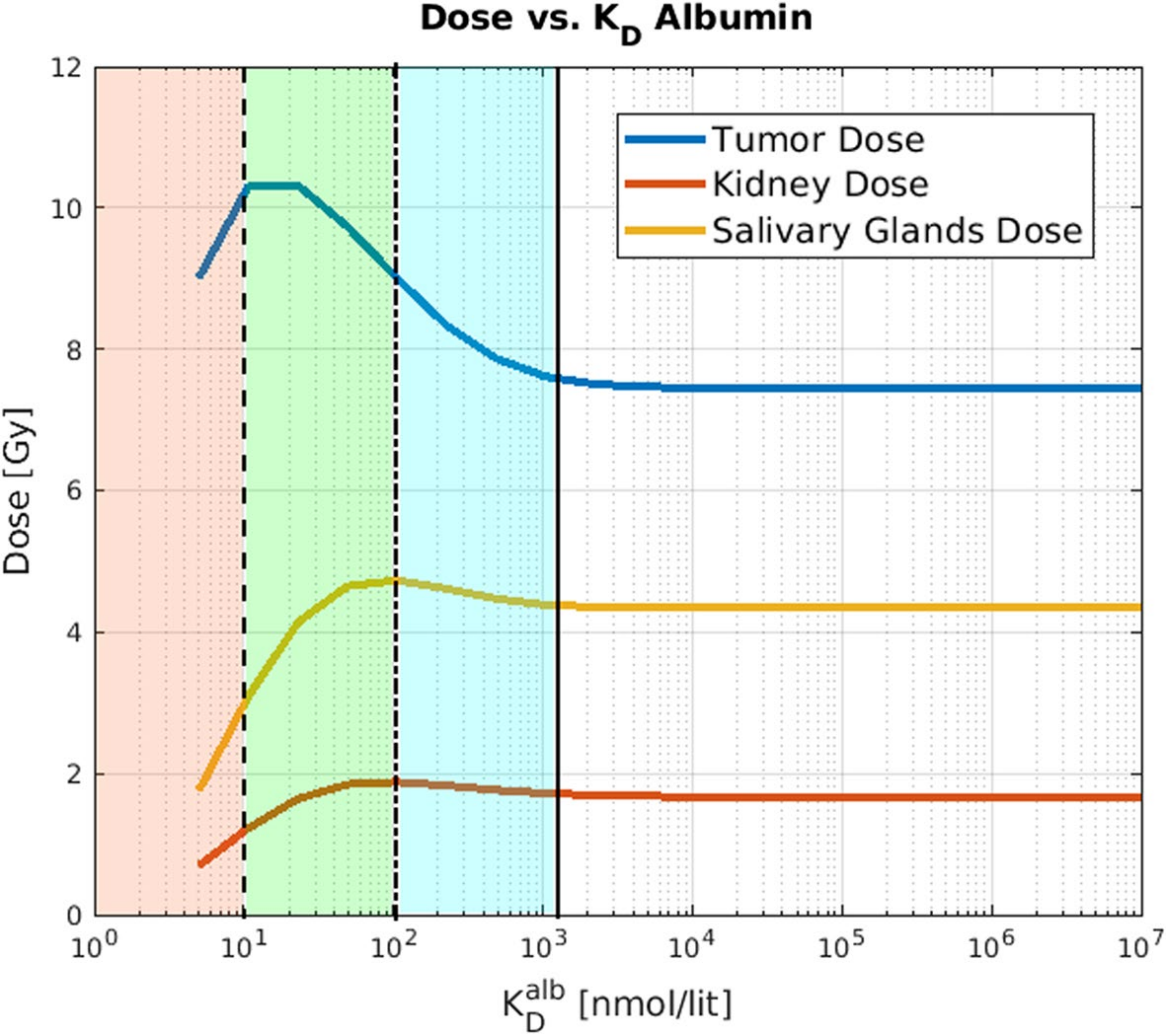
Dependence of dose on the dissociation constant of the albumin-ligand reaction. With higher albumin affinity (i.e. lower values of the dissociation constant), we observe that the dose to OAR and tumor both start to increase (as one moves to the left of the solid line) which is due to the increased residence time of the radiopharmaceuticals. However, for further lower dissociation value (beyond the dotted-dashed line), the dose to OAR starts decreasing while the dose to tumor still increases, attributed to leakage of albumin and albumin-bound ligands in the interstitial space of the tumor (see discussion). Finally, with much lower values of $K_D^{alb}$ (beyond the dashed line), all doses decrease related to the fact that with very strong affinities, there will be limited binding to receptors

Figure 9 shows that the blood residence time is increased with a higher affinity of the radiopharmaceutical to the albumin. Furthermore, Fig. 10 represents the absorbed dose by tumor and OARs as a function of dissociation constant. The vertical lines in this figure show different regions of the plot in which dose to tumor and OAR exhibit different behaviors in terms of decreases and increases (we discuss these regions in the figure caption and Discussion section).

**Fig. 11:**
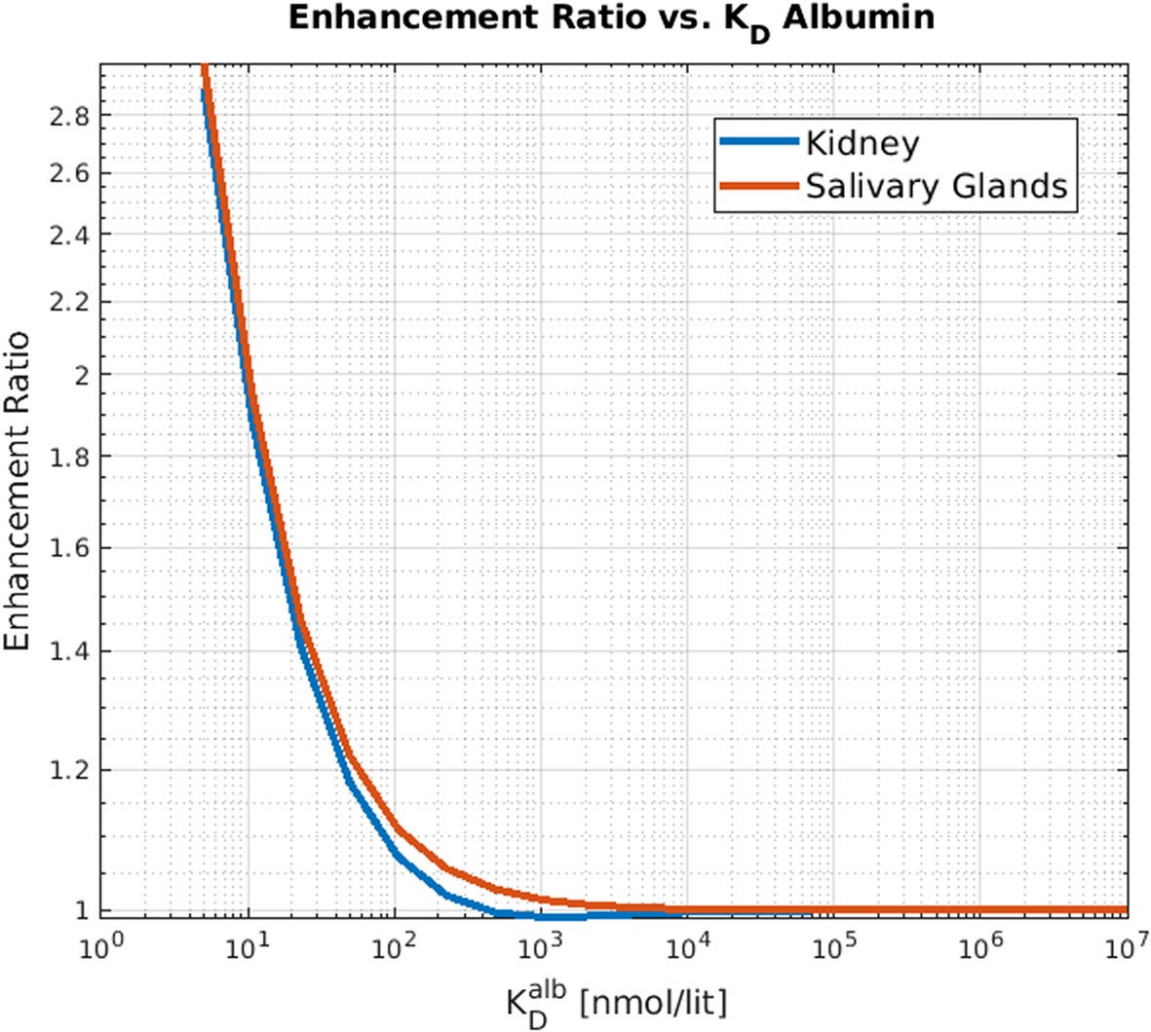
Dependence of enhancement factor on the dissociation rate of albumin-ligand interaction. Lower values of $K_D^{alb}$ lead to higher enhancement ratios, meaning that higher albumin affinity will help in delivering more dose to the tumor and sparing (to some degree) the OAR

Moreover, Fig. 11 depicts the enhancement factor for tumor dose with respect to salivary glands and kidney. It is seen that values increasingly greater than one are seen for the enhancement factor for increasing binding affinities to albumin. Overall, this computational framework enables the gaining of more insights into radiopharmaceutical development considerations.

## Discussion

### Model limitations

In general, PBPK models have many parameters and this can be both its strength and weakness (see Sager et al. 2015; Tan et al. 2018; Quijano-Mateos 2022; Khalil and Läer 2011 for more detailed discussions). It can be considered a strength because given enough accurate measurements of the parameters, the model can predict the behavior of complex interconnected systems. However, when dealing with few measured parameters, using PBPK models for accurate predictions requires significant care. In the present work, we focus on behavioral analysis of these models for a range of realistic parameters. Future efforts on personalization of PBPK models and digital twinning of patients (Rahmim et al. 2022) need to address the above-mentioned challenge. In this paper, we represented a scalable PBPK model structure and performed numerical explorations to find possible answers for questions of clinical importance to demonstrate the capabilities of PBPK models, also aiming to enable the community to use computational tools towards understanding and optimization of RPTs.

As discussed in the supplementary file, the reaction between albumin and ligand is assumed to be one-to-one (meaning only one ligand can bind to the albumin). This is not exactly true. The ligand can bind to different albumin binding sites thus having a larger-than-one stoichiometric coefficient, which is assumed to be 1 on our model. This assumption can be made more accurate following more investigations in this area. Furthermore, we have assumed that due to the larger size of albumin molecules, the albumin-ligand complex will not be able to bind to the binding sites. Also, we made the assumption that the albumin leakage to the tumor interstitial space is due to the porous structure of the vascular wall, thus its permeability coefficient in entering the interstitial space does not scale with its molecular weight, and in fact, we assumed the values are the same as the permeability coefficient of ligands (this assumption is worth further investigating; in the present work, it helps us obtain an upper bound for the effectiveness of albumin binding, which will help in designing future studies).

Our PBRPK model presented here reflects the kinetics of radiopharmaceuticals. However, making accurate statements about the number of cells killed because of a given activity profile requires yet another model that takes radio-biology into account. However, in this work, to limit the number of possible sources of errors to the conclusions, we did not include any radiobiological modeling. Needless to say, there are significant efforts towards reliable and accurate computational and mathematical models to describe the survival rate of cells in RPTs (EANM Radiobiology Working Group 2023) (which can be quite distinct from external beam radiotherapy), and our future investigations will include such models. In other words, while the present work focuses on pharmacokinetics (what the body does to a drug), pharmacodynamics (what the drug does to the body) which in our case involves radiation biology is an area of upcoming investigation, and upgrade to our PBRPK model.

### Hot and cold interaction

Considering two red dots on the iso-dose curve of the tumor in Fig. 1, we observe that despite the fact that they have the same delivered dose to the tumor, they are not achieved through the same injected activity, and one has almost half the injected activity of the other one; in other words, specific activities can play a significant role in delivered doses, which is a key consideration. Meanwhile, Figs. 4 and 5 suggest that the iso-dose curves become less parallel with respect to the very first iso-dose curve as we decrease tumor receptor densities especially, indicating significant impact in delivery of doses with varying injected radioactivities and specific activities.

Since smaller amounts of radioactive injection (i.e. low hot and cold amounts) fall on the very first iso-dose curve, and it has a certain relation between the hot and cold amount that results in the corresponding dose, we decided to compare other iso-dose curves with this particular one, given its almost constant behavior in all of the plots (i.e. can be thought of as a common ground between the figures). Obviously, we could select other common references between plots to perform the measurements, e.g. the vertical y-axis. However, a vertical iso-dose curve indicates that there is no competition between hot and cold species in binding to the receptors: a scenario where this can be achieved is when we have an infinite number of receptors, thus there is no limited common binding site for hot and cold. However, since this scenario is somehow hypothetical, the y-axis is not an appropriate reference for these measurements.

Lower twist values for the iso-dose curves for a given value of tumor receptor density and tumor volume indicate that the iso-dose curves associated with the high dose amount behave similarly to small injections. Lower twist value for a given hot-cold plot thus indicate lower receptor saturations.

Comparing Figs. 4 with 5 reveals that increasing the tumor receptor density decreases the receptor saturation in tumors but not in OAR. However, increasing the tumor volume will decrease the saturation in OAR as well. This is related to the tumor sink effect (Beauregard et al. 2012) wherein higher tumor volumes will inhibit uptake in OARs, and we show that this can result in decreased OAR saturation as well.

### Injection profile

A core observation in this work is that the delivered dose to tumors and OARs increases as we fractionate the injection more (i.e. injecting in smaller portions over time, relative to prevalent single bolus injections). The increasing trend in the effectiveness of multi-bolus injections as tumor receptor densities decrease (Fig. 8) is related to the fact that higher fractionation will help towards less saturation in the receptors, and thus more free ligands in the body are susceptible to clearance through the kidneys.

### Albumin

In utilizing the albumin kinetics for radiopharmaceutical delivery, three factors interact with each other, contributing to the 3 colored regions in Fig. 10 of different patterns of increase or decrease in tumor vs. OAR doses:

- **Kidney clearance:** Albumin is not cleared through the kidney, and as such, albumin-bound ligands will also not be removed from the body and will stay longer in the blood. This will result in higher blood residence time (see Fig. 9). This can explain the blue region in Fig. 10.
- **Porous vascular structure in the tumor microenvironment:** Due to the mechanically distorted and porous structure of the tumor vasculature, albumin (and also albumin-bound ligands) can leak into the interstitial space of the tumor but not the OAR. This can result in a higher dose to tumors but not OARs, resulting in the green region in Fig. 10.
- **The large size of albumin protein:** Albumin is a very large molecule (about 70 kD) compared to radiopharmaceuticals (about 1 kD). Thus the albumin-bound ligands will not be able to bind to the PSMA binding sites. This can result in a lower delivered dose to the tumors and OARs, resulting in the orange region (left-most) in Fig. 10.

As context for the present effort, we note that following successful exploitation of albumin binding mechanism for pharmaceuticals to enhance the therapeutic index (Lee and Wu 2015; Fan et al. 2022), there has been an increased motivation in the nuclear medicine community to study the effect of albumin binding in radiopharmaceuticals (Boinapally et al. 2023; Szücs et al. 2023; Busslinger et al. 2023; Brandt et al. 2022; Alati et al. 2023). However, to our best knowledge, there have been no computational models to study phenomena in this domain, motivating our design of a scalable PBRPK model and computational studies in albumin binding.

## Conclusion

In this work, we have extended PBPK modeling and made it more suitable for simulating complicated kinetics of radiopharmaceuticals. The new PBRPK model is publicly shared and easy to implement computationally. It is easy to expand the model and to include ligand-protein interactions. We implemented the model in MATLAB Simbiology, and shared the SBML implementation with the community. Using the new PBRPK model, we studied the interactions between hot and cold ligands and found out that cold ligands can saturate the receptors and leave limited binding sites available for the hot ligands, demonstrating that specific activities of injected radiopharmaceuticals can make a crucial difference. In addition, we studied the effect of multi-bolus injection on the efficiency of dose delivery to tumors and OAR. We found that fractionating the injection can lead to a higher payload (i.e. higher delivered doses per unit injected activity), though a differential advantage was not observed (i.e. no further sparing of OARs with respect to tumor doses). Moreover, we found that higher affinity of the ligands to albumin can lead to significant differential advantages in delivering doses to tumors, and provided more insights into phenomena involved towards optimal radiopharmaceutical therapies.

## Model sharing

The latest version of the PBPK model in SBML format can be found in the following repository: https://github.com/alifele/Computational-Physics/tree/main/PSMA-PBPK-Model-Matlab

The various parameters used in the model are also provided as Additional file 1: supplemental material.

### Supplementary Information

**Additional file 1**. Detailed overview of the structure of the PBPK model, and tabulated parameter values used to run the simulations.

## Data Availability

This work has no associated patient data. The latest version of the PBPK model in SBML format can be found in the following repository: https://github.com/alifele/Computational-Physics/tree/main/PSMA-PBPK-Model-Matlab The various parameters used in the model are also provided as Additional file 1: supplemental material.
