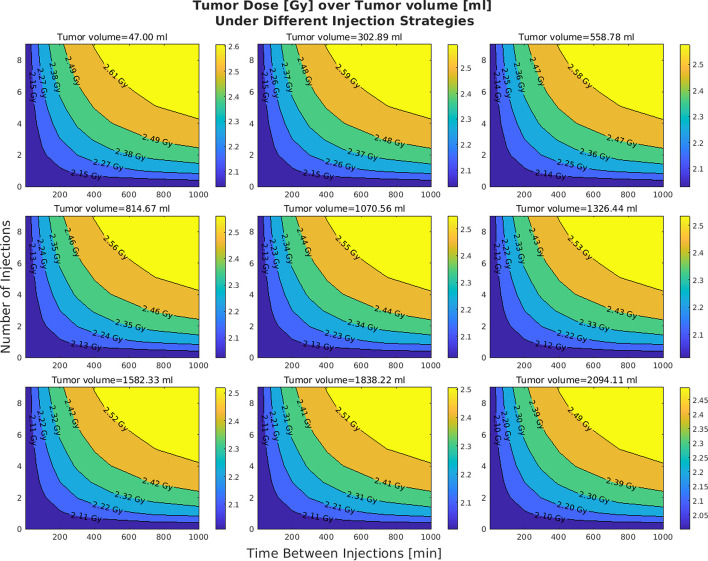# Correction: Physiologically based radiopharmacokinetic (PBRPK) modeling to simulate and analyze radiopharmaceutical therapies: studies of non-linearities, multi-bolus injections, and albumin binding

**DOI:** 10.1186/s41181-024-00251-5

**Published:** 2024-03-19

**Authors:** Ali Fele‑Paranj, Babak Saboury, Carlos Uribe, Arman Rahmim

**Affiliations:** 1 School of Biomedical Engineering, University of British Columbia, Vancouver, BC Canada; 2 Department of Integrative Oncology, BC Cancer Research Institute, Vancouver, BC Canada; 3 Department of Radiology and Imaging Sciences, Clinical Center, National Institutes of Health, Bethesda, MD USA; 4 Department of Functional Imaging, BC Cancer, Vancouver, BC Canada; 5 Department of Radiology, University of British Columbia, Vancouver, BC Canada

**Correction: EJNMMI Radiopharmacy and Chemistry (2024) 9:6** 10.1186/s41181-023-00236-w

In the original publication of the article, inaccuracies were identified in Figures 3 and 7 due to an incorrect application of S values in the dose calculations. The S values, which should vary with tumor volume to accurately represent dose distribution, were mistakenly kept constant.

Upon review, the authors have corrected this error and recalculated the doses with varying 1/s values as they should proportionally change with tumor sizes (volumes). Below is a Table 1 of the corrected S values and their corresponding tumor volumes:

**Table 1:** Corrected S values and corresponding tumor volumes

Tumor volume (ml)	S value (Gy ∗ min^−1^ ∗ MBq^−1^)
47	3.01E−05
302	4.67E−06
558	2.53E−06
814	1.73E−06
1070	1.32E−06
1326	1.06E−06
1582	8.95E−07
1838	7.70E−07
2094	6.76E−07
2350	6.02E−07

These errors also affected figures 3 and 7. The incorrect and correct figures are shown in this correction article. The corrected figures show that with increasing volumes the distributions are minimally altered; all other figures and conclusions remain intact.

**Incorrect figure 3** 
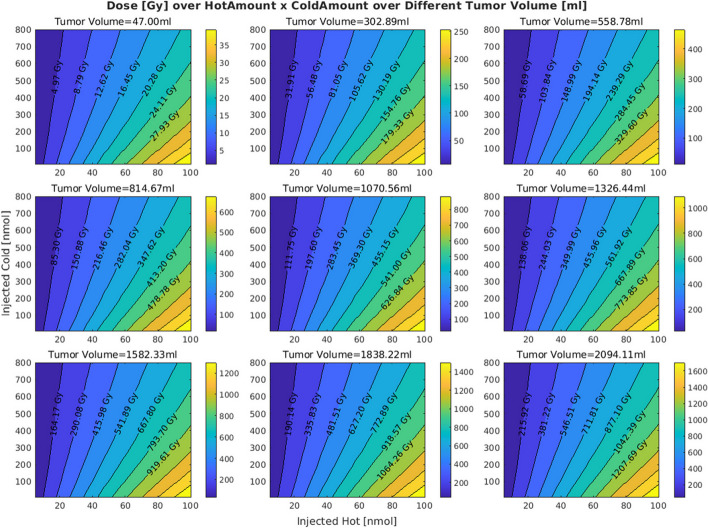


**Incorrect figure 7** 
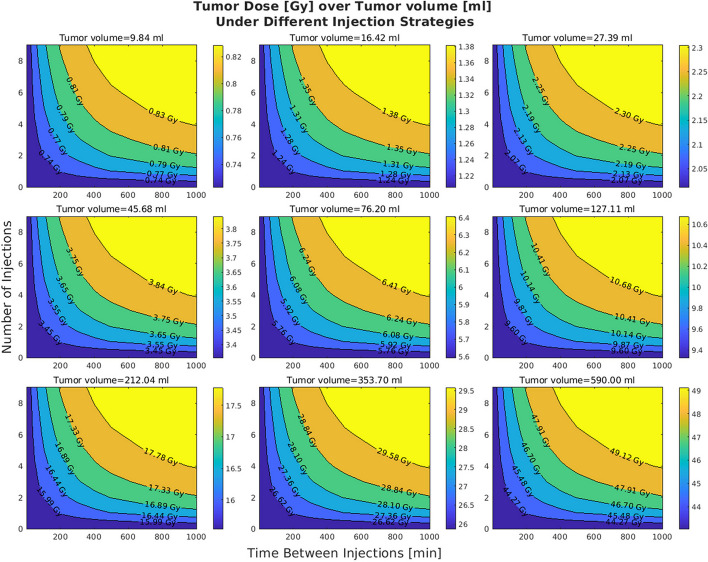


**Correct figure 3** 
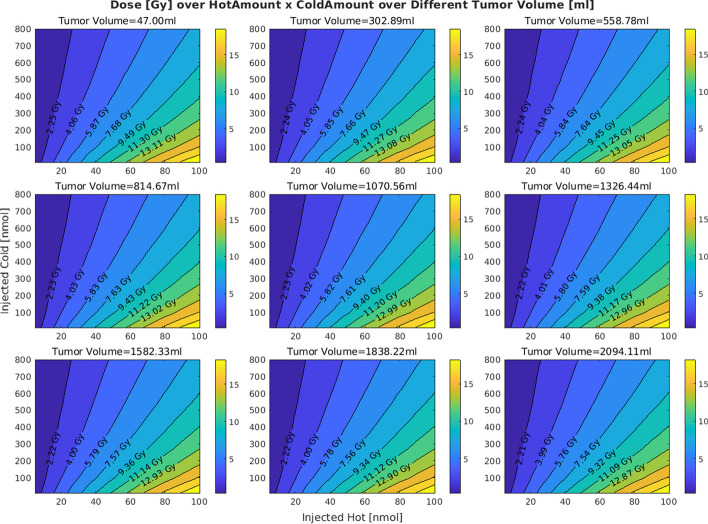


**Correct figure 7**