# Supplementary material for: Physiologically based radiopharmacokinetic (PBRPK) modeling to simulate and analyze radiopharmaceutical therapies: studies of non-linearities, multi-bolus injections, and albumin binding
Source: EJNMMI Radiopharm Chem. 2024 Jan 22;9:6. doi: 10.1186/s41181-023-00236-w (PMC10803696; doi:10.1186/s41181-023-00236-w)
Supplement: Supplementary file 1 — Additional file 1. Detailed overview of the structure of the PBPK model, and tabulated parameter values used to run the simulations. [file 41181_2023_236_MOESM1_ESM.pdf]

S Supplementary Material

This supplement covers the details of the PBRPK model discussed in the presented paper. The information shared here is enough to enable recreating the model from scratch and regenerating the results.

S.1 Naming Convention for Variables

It is often very common in the community of mathematics to show different variables with single letters such as x, α, etc. However, this approach is not really scalable and becomes very challenging in assigning names to variables when there are lots of them in a model. Hence we developed our own naming convention in developing our PBRPK model which makes it easily scalable.

The naming conventions are quite similar to the strategy when we use object-oriented programming. For instance, suppose that you want to refer to the hot (i.e. labeled) radiopharmaceuticals in the interstitial space of the liver. The name of that variable will be like:

Liver.int.H

in which "int" reflects the interstitial space, and "H" reflects the hot radiopharmaceuticals.

S.2 Structure of the model

As we discussed in the presented paper, the PBRPK model is created by connecting different organs to each other using ordinary differential equations. Lungs, heart, adipose, skin, bone, red marrow, and muscle are considered as the receptor-negative organs (that do not express any PSMA binding sites), while the liver, spleen, salivary glands, prostate, GI, tumor, and kidney are considered as receptor-positive organs which means that they effectively express PSMA binding sites.

Receptor-negative organs do not have any radiopharmaceutical uptake while the receptor-positive organs have radiopharmaceutical uptakes. This uptake can be in the form of specific uptake (bound to actual receptors), or non-specific uptake (which still can be modeled as binding to some imaginary receptors. The organs in each category have a very similar structure and are only distinguished due to the different parameters that they have. In the following section, we will explain the internal structure of each category of organs.

S.2.1 Internal Structure of the Organs in PBRPK

All receptor-negative organs have a similar structure which is shown in Figure S1. The only exception is the lungs in which the inflow of blood into the organ is from veins (instead of arteries) and the outflow of blood from the lungs is to the arteries.

In contrast, for the receptor-positive organs, we will have slightly different structures to take the receptor binding and internalization process into account (see Figure S2) However, due to the complex nature of the kidney, it has its own dedicated structure design to take the kidney functions into account (see Figure S3).

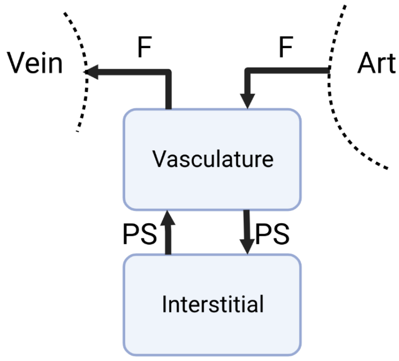

Fig. S1: Compartmentalized structure of the receptor-negative organs. Note that there are no “bound” and “intern” compartments, which reflects the fact that the organ is receptor-negative.

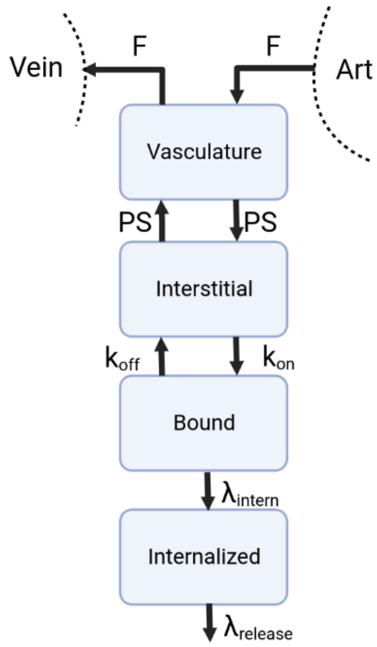

Fig. S2: Compartmentalized structure of the receptor-positive organs.

At each compartment (as the inner structure of the organs), there are generally five distinct species called H, C, AH, AC, Alb which stands for hot and cold (labeled and unlabeled) radiopharmaceuticals, hot-albumin and cold-albumin complex and albumin itself respectively. These species interact with each other which can be summarized in a “reaction graph” (see Figure S4).

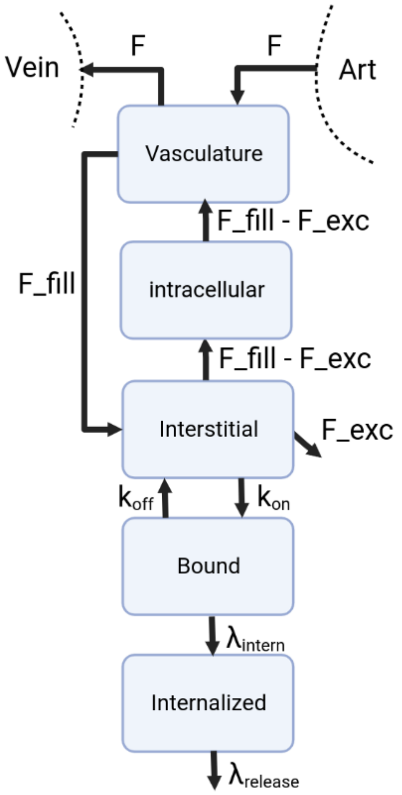

Fig. S3: Compartmentalized structure of the kidney. The distinct feature of kidney in filtering the blood leads to its different structure than other receptor-positive organs.

This reaction graph can be fully described by a system of ODEs as following

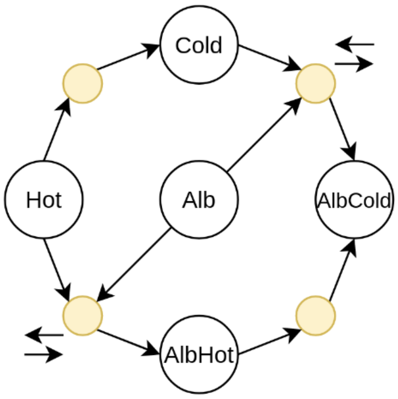

Fig. S4: Interactions of the different species with each other. The yellow circles represent the reactions between species and those circles labeled with a two-way arrow indicate that the reaction is a two-way reaction. The reactions that are not two-way are the decay process of radiopharmaceuticals.

$$\frac{dH}{dt} = -\lambda H - k_{on}^{alb} H * [Alb] + k_{off}^{alb} AH \tag{S1}$$

$$\frac{dC}{dt} = \lambda H - k_{on}^{alb} C * [Alb] + k_{off}^{alb} AC \tag{S2}$$

$$\frac{dAH}{dt} = -\lambda AH + k_{on}^{alb} H * [Alb] - k_{off}^{alb} AH \tag{S3}$$

$$\frac{dAC}{dt} = \lambda AH + k_{on}^{alb} C * [Alb] - k_{off}^{alb} AC \tag{S4}$$

$$Alb = Alb_0 - AH - AC \tag{S5}$$

Using the fact that these species are repeated in each compartment, we can now describe the concentration of each of these species in each compartment by writing a differential equation. Because the ODE for all receptor-negative organs (same as receptor-positive organs) looks similar to each other (except for the numerical values of the parameters as well as the variable names), we explicitly write the ODE system for just one organ from each category here. However, the full details of all of the equations can be found in the table (auto-generated by the Simbiology package in Matlab) attached at the end of this supplementary file (see subsection S.4).

The ODE system for any receptor-negative organ (name it X) will be like this:

$$\frac{dX.vas.H}{dt} = Q_1 - \lambda X.vas.H - k_{on}^{alb} X.vas.H * [X.vas.Alb] + k_{off}^{alb} X.vas.AH \tag{S6}$$

$$\frac{dX.vas.C}{dt} = Q_2 + \lambda X.vas.H - k_{on}^{alb} X.vas.C * [X.vas.Alb] + k_{off}^{alb} X.vas.AC \tag{S7}$$

$$\frac{dX.vas.AH}{dt} = Q_3 - \lambda X.vas.AH + k_{on}^{alb} X.vas.H * [X.vas.Alb] - k_{off}^{alb} X.vas.AH \tag{S8}$$

$$\frac{dX.vas.AC}{dt} = Q_4 + \lambda X.vas.AH + k_{on}^{alb} X.vas.C * [X.vas.Alb] - k_{off}^{alb} X.vas.AC \tag{S9}$$

$$\frac{dX.int.H}{dt} = Q_5 - \lambda X.int.H \tag{S10}$$
$$\frac{dX.int.C}{dt} = Q_6 + \lambda X.int.H \tag{S11}$$
$$X.vas.Alb = X.vas.Alb_0 - X.vas.AH - X.vas.AC \tag{S12}$$

in which we have

$$Q_1 = F_X([art.H] - [X.vas.H]) + PS_X([X.int.H] - [X.vas.H]) \tag{S13}$$
$$Q_2 = F_X([art.C] - [X.vas.C]) + PS_X([X.int.C] - [X.vas.C]) \tag{S14}$$
$$Q_3 = F_X([art.AH] - [X.vas.AH]) + PS_X([X.int.AH] - [X.vas.AH]) \tag{S15}$$
$$Q_4 = F_X([art.AC] - [X.vas.AC]) + PS_X([X.int.AC] - [X.vas.AC]) \tag{S16}$$
$$Q_5 = PS_X([X.vas.H] - [X.int.H]) \tag{S17}$$
$$Q_6 = PS_X([X.vas.C] - [X.int.C]) \tag{S18}$$

Note that the variables inside bracket $[\cdot]$ represent the concentration of that variable. For instance, we have

$$[X.vas.H] = \frac{X.vas.H}{V_{\mathbf{X,v}}}$$

in which $V_{\mathbf{X,v}}$ is the interstitial volume of the organ X.

Similarly, we can write the ODE system for the receptor-positive systems. The ODE system for the reaction graph for receptor-positive organs will be like the following:

$$\frac{dX.vas.H}{dt} = T_1 - \lambda X.vas.H - k_{on}^{alb} X.vas.H * [X.vas.Alb] + k_{off}^{alb} X.vas.AH \tag{S19}$$
$$\frac{dX.vas.C}{dt} = T_2 + \lambda X.vas.H - k_{on}^{alb} X.vas.C * [X.vas.Alb] + k_{off}^{alb} X.vas.AC \tag{S20}$$
$$\frac{dX.vas.AH}{dt} = T_3 - \lambda X.vas.AH + k_{on}^{alb} X.vas.H * [X.vas.Alb] - k_{off}^{alb} X.vas.AH \tag{S21}$$
$$\frac{dX.vas.AC}{dt} = T_4 + \lambda X.vas.AH + k_{on}^{alb} X.vas.C * [X.vas.Alb] - k_{off}^{alb} X.vas.AC \tag{S22}$$
$$\frac{dX.int.H}{dt} = T_5 - \lambda X.int.H - k_{on}^{alb} X.int.H * [X.int.Alb] + k_{off}^{alb} X.int.AH \tag{S23}$$
$$\frac{dX.int.C}{dt} = T_6 + \lambda X.int.H - k_{on}^{alb} X.int.C * [X.int.Alb] + k_{off}^{alb} X.int.AC \tag{S24}$$
$$\frac{dX.int.AH}{dt} = T_7 - \lambda X.int.AH + k_{on}^{alb} X.int.H * [X.int.Alb] - k_{off}^{alb} X.int.AH \tag{S25}$$
$$\frac{dX.int.AC}{dt} = T_8 + \lambda X.int.AH + k_{on}^{alb} X.int.C * [X.int.Alb] - k_{off}^{alb} X.int.AC \tag{S26}$$
$$\frac{dX.bound.H}{dt} = T_9 - \lambda X.int.H - k_{on}^{alb} X.int.H * [X.int.Alb] + k_{off}^{alb} X.int.AH \tag{S27}$$
$$\frac{dX.bound.C}{dt} = T_{10} + \lambda X.int.H - k_{on}^{alb} X.int.C * [X.int.Alb] + k_{off}^{alb} X.int.AC \tag{S28}$$
$$\frac{dX.intern.H}{dt} = T_{11} - \lambda X.int.H - k_{on}^{alb} X.int.H * [X.int.Alb] + k_{off}^{alb} X.int.AH \tag{S29}$$
$$\frac{dX.intern.C}{dt} = T_{12} + \lambda X.int.H - k_{on}^{alb} X.int.C * [X.int.Alb] + k_{off}^{alb} X.int.AC \tag{S30}$$
$$X.vas.Alb = X.vas.Alb_0 - X.vas.AH - X.vas.AC \tag{S31}$$

$$X.int.Alb = X.int.Alb_0 - X.int.AH - X.int.AC \tag{S32}$$

in which we have

$$T_1 = F_X([art.H] - [X.vas.H]) + PS_X([X.int.H] - [X.vas.H]) \tag{S33}$$

$$T_2 = F_X([art.C] - [X.vas.C]) + PS_X([X.int.C] - [X.vas.C]) \tag{S34}$$

$$T_3 = F_X([art.AH] - [X.vas.AH]) + PS_X([X.int.AH] - [X.vas.AH]) \tag{S35}$$

$$T_4 = F_X([art.AC] - [X.vas.AC]) + PS_X([X.int.AC] - [X.vas.AC]) \tag{S36}$$

$$T_5 = PS_X([X.vas.H] - [X.int.H]) - k_{on}X.R[X.int.H] + k_{off}X.bound.H \tag{S37}$$

$$T_6 = PS_X([X.vas.C] - [X.int.C]) - k_{on}X.R[X.int.C] + k_{off}X.bound.C \tag{S38}$$

$$T_7 = PS_X([X.vas.AC] - [X.int.AC]) - k_{on}X.R[X.int.AC] + k_{off}X.bound.AC \tag{S39}$$

$$T_8 = PS_X([X.vas.AH] - [X.int.AH]) - k_{on}X.R[X.int.AH] + k_{off}X.bound.AH \tag{S40}$$

$$T_9 = k_{on}X.R[X.int.H] - (k_{off} + X.\lambda_{int})X.bound.H \tag{S41}$$

$$T_{10} = k_{on}X.R[X.int.C] - (k_{off} + X.\lambda_{int})X.bound.C \tag{S42}$$

$$T_{11} = X.\lambda_{int}X.bound.H - X.\lambda_{rel}X.intern.H \tag{S43}$$

$$T_{12} = X.\lambda_{int}X.bound.C - X.\lambda_{rel}X.intern.C \tag{S44}$$

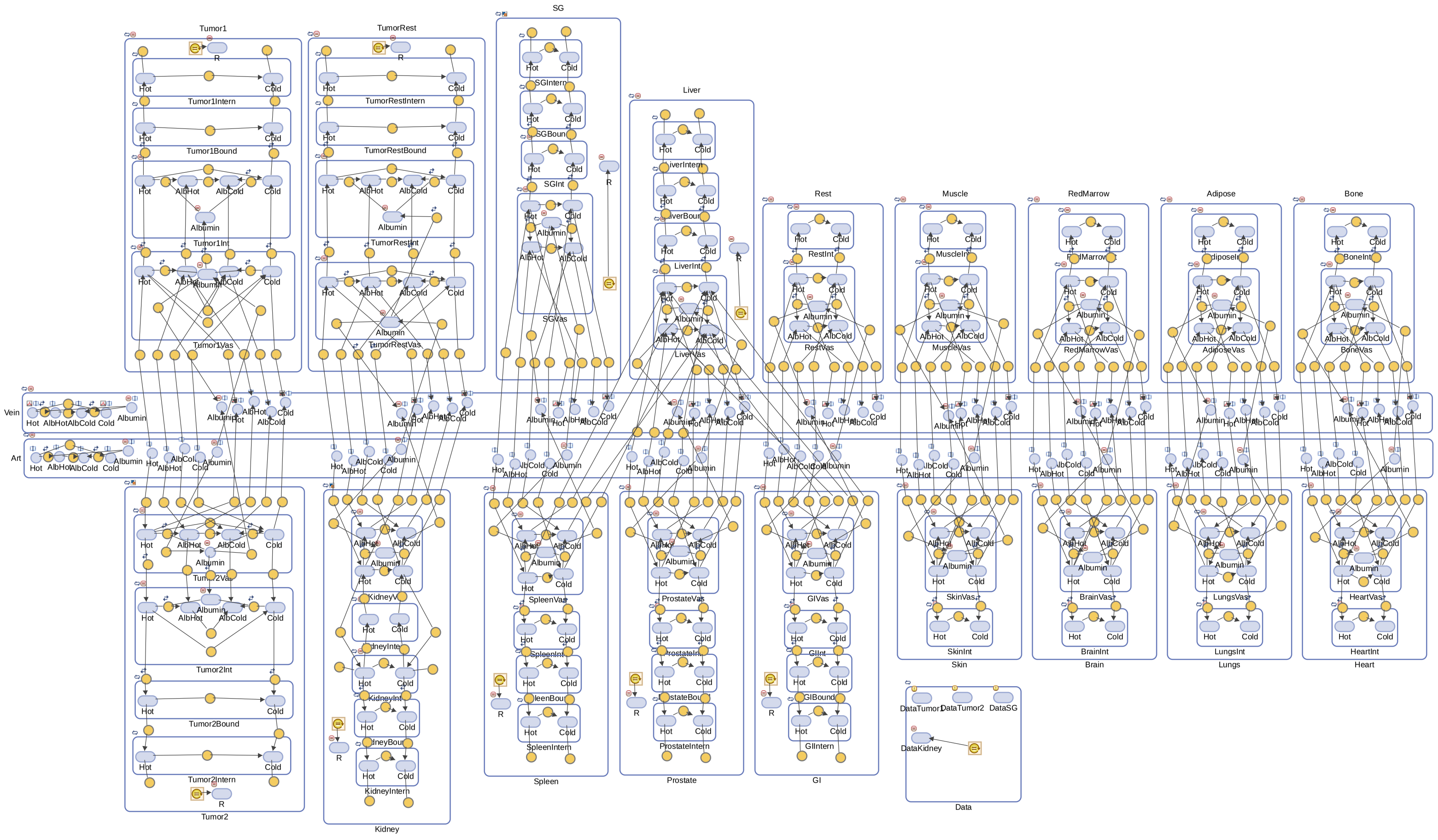

Fig. S5: The structure of PBPK model as implemented in Simbiology Matlab.

S.3 S-Values and Dose Calculation

Understanding the concept of radiation dose and radiation dose rate, as well as their units of measurement, is crucial for calculating radiation dose. Radiation dose, specifically radiation absorbed dose (D), quantifies the total energy absorbed by one unit of mass of a substance. In the SI Unit System, the absorbed dose is measured in gray (Gy), where 1 Gy equals 1 joule (J) of energy absorbed per kilogram of substance. Commonly used derived units from gray are centigray (cGy = 10^{-2} Gy) and milligray (mGy = 10^{-3} Gy). The older unit of radiation dose is rad, which represents 100 ergs (1 erg = 10^{-7}J) of absorbed energy per gram of tissue or substance. One rad is equivalent to (1/100) Gy or one cGy, and 1 Gy equals 100 rad. On the other hand, radiation dose rate (dD/dt) measures the amount of energy absorbed per unit of time per unit mass of tissue. It can be expressed in units such as mGy (rad) per minute, cGy (rad) per hour, or Gy (rad) per day.

In order to calculate radiation dose, it is necessary to determine the average energy absorbed by 1 gram of tissue in a target organ from the total energy released by the decay of a specific amount of radioactivity. Since x-rays or γ-rays are highly penetrating, a small source emitting these types of radiation, localized anywhere in the body, will irradiate virtually all organs. For instance, if a radionuclide emitting x-rays or γ-rays is localized solely in the liver (source S), it will deliver radiation dose not only to the liver but also to all other organs (targets T) in the body. Therefore, when a radiopharmaceutical is localized in multiple organs (sources), the radiation dose from each source to each organ (target) must be calculated. Only then can all the contributions be added together to determine the final radiation dose for each organ.

The process of determining radiation dose involves essential steps [1]. Firstly, calculate the rate of energy emission (erg/h) from the different types of radiation emitted by the radionuclide within the source volume. Next, determining the rate of energy absorption from these radiations by the target volume. Next, from the above-mentioned, calculating the average dose rate, dD/dt. This dose rate can be captured via the S-Values (shown in Table S1 and Table S2 for Lu-177 self-absorbed doses). Finally, calculating the average dose, D, by multiplying the S-values with the time-integrated activity (TIA). S-values rely on physical data, such as decay characteristics, and organ shape and size. On the other hand, the TIA necessitates radiopharmaceutical distribution data.

Table S1: S-Values for tumor and salivary glands. Table adapted from [2].

Volume [ml]	S-values $[Gy * min^{-1} * MBq^{-1}]$
0.5	$2.77 * 10^{-3}$
1	$1.40 * 10^{-3}$
1.5	$9.00 * 10^{-4}$
2	$7.02 * 10^{-4}$
3	$4.50 * 10^{-4}$
4	$3.52 * 10^{-4}$
13	$1.10 * 10^{-4}$
17	$8.30 * 10^{-5}$
21	$6.90 * 10^{-5}$
29	$4.80 * 10^{-5}$
34	$4.30 * 10^{-5}$
52	$2.67 * 10^{-5}$
54	$2.65 * 10^{-5}$

Table S2: S-Values used for kidney dose calculation. Table adapted from [2].

Organ	S-values $[Gy * min^{-1} * MBq^{-1}]$
Kidney	$4.82 * 10^{-6}$

S.4 Parameter Values

The following table summarizes the parameter values used for the simulation. Note that the values printed in the "initial values" column are the values used as the initial condition to solve the ODE system. The values printed under the "value" column are intermediate values that are transformed into initial values by the "initial assignments", which are also listed as separate tables in this section.

Model: testModel

Compartments

	Compartment Name	Scope	Value	Initial Value	Units
1	Tumor2Vas	Tumor2	1	0.0102	liter
2	Tumor2	testModel	1	0.34	liter
3	Tumor2Int	Tumor2	1	0.1292	liter
4	Tumor2Bound	Tumor2	1	1	liter
5	Tumor2Intern	Tumor2	1	1	liter
6	Art	testModel	1	0.33516	liter
7	Vein	testModel	1	0.7182	liter
8	Tumor1	testModel	1	0.01	liter
9	Tumor1Vas	Tumor1	1	0.0003	liter
10	Tumor1Int	Tumor1	1	0.0038	liter
11	Tumor1Bound	Tumor1	1	1	liter
12	Tumor1Intern	Tumor1	1	1	liter
13	Kidney	testModel	1	0.311	liter
14	KidneyVas	Kidney	1	0.017105	liter
15	KidneyInt	Kidney	1	0.04665	liter
16	KidneyBound	Kidney	1	1	liter
17	KidneyIntern	Kidney	1	1	liter
18	Heart	testModel	1	0.48028	liter
19	HeartVas	Heart	1	0.03192	liter
20	HeartInt	Heart	1	0.1181	liter
21	SG	testModel	1	0.021	liter
22	SGVas	SG	1	0.000378	liter
23	SGInt	SG	1	0.00483	liter
24	SGBound	SG	1	1	liter
25	SGIntern	SG	1	1	liter
26	Bone	testModel	1	12.7676	liter
27	BoneVas	Bone	1	0.09576	liter
28	BoneInt	Bone	1	0.80438	liter
29	TumorRest	testModel	1	0.048872	liter
30	TumorRestVas	TumorRest	1	0.0014662	liter
31	TumorRestInt	TumorRest	1	0.018571	liter
32	TumorRestBound	TumorRest	1	1	liter
33	TumorRestIntern	TumorRest	1	1	liter
34	Spleen	testModel	1	0.183	liter
35	SpleenVas	Spleen	1	0.02196	liter
36	SpleenInt	Spleen	1	0.0366	liter
37	SpleenBound	Spleen	1	1	liter
38	SpleenIntern	Spleen	1	1	liter
39	Liver	testModel	1	1.91	liter
40	LiverVas	Liver	1	0.16235	liter
41	LiverInt	Liver	1	0.382	liter
42	LiverBound	Liver	1	1	liter
43	LiverIntern	Liver	1	1	liter
44	Prostate	testModel	1	0.022535	liter
45	ProstateVas	Prostate	1	5.4085e-05	liter
46	ProstateInt	Prostate	1	0.0056338	liter
47	ProstateBound	Prostate	1	1	liter
48	ProstateIntern	Prostate	1	1	liter
49	GI	testModel	1	1.6718	liter
50	GIVas	GI	1	0.24259	liter
51	GIInt	GI	1	2.1348	liter
52	GIBound	GI	1	1	liter
53	GIIntern	GI	1	1	liter

54	Rest	testModel	1	11.5046	liter
55	RestVas	Rest	1	1.4165	liter
56	RestInt	Rest	1	5.8076	liter
57	Skin	testModel	1	4.8	liter
58	SkinVas	Skin	1	0.09576	liter
59	SkinInt	Skin	1	0.85226	liter
60	Muscle	testModel	1	42.3634	liter
61	MuscleVas	Muscle	1	0.44688	liter
62	MuscleInt	Muscle	1	2.6366	liter
63	Brain	testModel	1	2.0423	liter
64	BrainVas	Brain	1	0.038304	liter
65	BrainInt	Brain	1	1	liter
66	RedMarrow	testModel	1	1.5493	liter
67	RedMarrowVas	RedMarrow	1	0.12768	liter
68	RedMarrowInt	RedMarrow	1	0.47242	liter
69	Lungs	testModel	1	1.4085	liter
70	LungsVas	Lungs	1	0.33516	liter
71	LungsInt	Lungs	1	1.8434	liter
72	Adipose	testModel	1	18.9648	liter
73	AdiposeVas	Adipose	1	0.1596	liter
74	AdiposeInt	Adipose	1	2.4738	liter
75	KidneyIntera	Kidney	1	0.16483	liter
76	Data	testModel	1	1	liter

Species in Tumor2Vas

	Species Name	Value	Initial Value	Units
1	Hot	0	0	nanomole
2	Cold	0	0	nanomole
3	AlbHot	0	0	nanomole
4	AlbCold	0	0	nanomole
5	Albumin	0	0	nanomole

Species in Tumor2

	Species Name	Value	Initial Value	Units
1	R	0	6.46	nanomole

Species in Tumor2Int

	Species Name	Value	Initial Value	Units
1	Hot	0	0	nanomole
2	Cold	0	0	nanomole
3	AlbHot	0	0	nanomole
4	AlbCold	0	0	nanomole
5	Albumin	0	0	nanomole

Species in Tumor2Bound

	Species Name	Value	Initial Value	Units
1	Hot	0	0	nanomole
2	Cold	0	0	nanomole

Species in Tumor2Intern

	Species Name	Value	Initial Value	Units
1	Hot	0	0	nanomole
2	Cold	0	0	nanomole

Species in Art

	Species Name	Value	Initial Value	Units
1	Hot	0	0	nanomole
2	Cold	0	0	nanomole
3	AlbHot	0	0	nanomole

4	AlbCold	0	0	nanomole
5	Albumin	0	0	nanomole

Species in Vein

	Species Name	Value	Initial Value	Units
1	Hot	0	0	nanomole
2	Cold	0	0	nanomole
3	AlbHot	0	0	nanomole
4	AlbCold	0	0	nanomole
5	Albumin	0	0	nanomole

Species in Tumor1

	Species Name	Value	Initial Value	Units
1	R	0	0.57	nanomole

Species in Tumor1Vas

	Species Name	Value	Initial Value	Units
1	Hot	0	0	nanomole
2	Cold	0	0	nanomole
3	AlbHot	0	0	nanomole
4	AlbCold	0	0	nanomole
5	Albumin	0	0	nanomole

Species in Tumor1Int

	Species Name	Value	Initial Value	Units
1	Hot	0	0	nanomole
2	Cold	0	0	nanomole
3	AlbHot	0	0	nanomole
4	AlbCold	0	0	nanomole
5	Albumin	0	0	nanomole

Species in Tumor1Bound

	Species Name	Value	Initial Value	Units
1	Hot	0	0	nanomole
2	Cold	0	0	nanomole

Species in Tumor1Intern

	Species Name	Value	Initial Value	Units
1	Hot	0	0	nanomole
2	Cold	0	0	nanomole

Species in Kidney

	Species Name	Value	Initial Value	Units
1	R	0	4.354	nanomole

Species in KidneyVas

	Species Name	Value	Initial Value	Units
1	Hot	0	0	nanomole
2	Cold	0	0	nanomole
3	AlbHot	0	0	nanomole
4	AlbCold	0	0	nanomole
5	Albumin	0	0	nanomole

Species in KidneyInt

	Species Name	Value	Initial Value	Units
1	Hot	0	0	nanomole
2	Cold	0	0	nanomole

Species in KidneyBound

	Species Name	Value	Initial Value	Units

1	Hot	0	0	nanomole
2	Cold	0	0	nanomole

Species in KidneyIntern

	Species Name	Value	Initial Value	Units
1	Hot	0	0	nanomole
2	Cold	0	0	nanomole

Species in HeartVas

	Species Name	Value	Initial Value	Units
1	Hot	0	0	nanomole
2	Cold	0	0	nanomole
3	AlbHot	0	0	nanomole
4	AlbCold	0	0	nanomole
5	Albumin	0	0	nanomole

Species in HeartInt

	Species Name	Value	Initial Value	Units
1	Hot	0	0	nanomole
2	Cold	0	0	nanomole

Species in SG

	Species Name	Value	Initial Value	Units
1	R	0	0.798	nanomole

Species in SGVas

	Species Name	Value	Initial Value	Units
1	Hot	0	0	nanomole
2	Cold	0	0	nanomole
3	AlbHot	0	0	nanomole
4	AlbCold	0	0	nanomole
5	Albumin	0	0	nanomole

Species in SGInt

	Species Name	Value	Initial Value	Units
1	Hot	0	0	nanomole
2	Cold	0	0	nanomole

Species in SGBound

	Species Name	Value	Initial Value	Units
1	Hot	0	0	nanomole
2	Cold	0	0	nanomole

Species in SGIntern

	Species Name	Value	Initial Value	Units
1	Hot	0	0	nanomole
2	Cold	0	0	nanomole

Species in BoneVas

	Species Name	Value	Initial Value	Units
1	Hot	0	0	nanomole
2	Cold	0	0	nanomole
3	AlbHot	0	0	nanomole
4	AlbCold	0	0	nanomole
5	Albumin	0	0	nanomole

Species in BoneInt

	Species Name	Value	Initial Value	Units
1	Hot	0	0	nanomole
2	Cold	0	0	nanomole

Species in TumorRest

	Species Name	Value	Initial Value	Units
1	R	0	13	nanomole

Species in TumorRestVas

	Species Name	Value	Initial Value	Units
1	Hot	0	0	nanomole
2	Cold	0	0	nanomole
3	AlbHot	0	0	nanomole
4	AlbCold	0	0	nanomole
5	Albumin	0	0	nanomole

Species in TumorRestInt

	Species Name	Value	Initial Value	Units
1	Hot	0	0	nanomole
2	Cold	0	0	nanomole
3	AlbHot	0	0	nanomole
4	AlbCold	0	0	nanomole
5	Albumin	0	0	nanomole

Species in TumorRestBound

	Species Name	Value	Initial Value	Units
1	Hot	0	0	nanomole
2	Cold	0	0	nanomole

Species in TumorRestIntern

	Species Name	Value	Initial Value	Units
1	Hot	0	0	nanomole
2	Cold	0	0	nanomole

Species in Spleen

	Species Name	Value	Initial Value	Units
1	R	0	0.097356	nanomole

Species in SpleenVas

	Species Name	Value	Initial Value	Units
1	Hot	0	0	nanomole
2	Cold	0	0	nanomole
3	AlbHot	0	0	nanomole
4	AlbCold	0	0	nanomole
5	Albumin	0	0	nanomole

Species in SpleenInt

	Species Name	Value	Initial Value	Units
1	Hot	0	0	nanomole
2	Cold	0	0	nanomole

Species in SpleenBound

	Species Name	Value	Initial Value	Units
1	Hot	0	0	nanomole
2	Cold	0	0	nanomole

Species in SpleenIntern

	Species Name	Value	Initial Value	Units
1	Hot	0	0	nanomole
2	Cold	0	0	nanomole

Species in Liver

	Species Name	Value	Initial Value	Units
1	R	0	2.5403	nanomole

Species in LiverVas

	Species Name	Value	Initial Value	Units
1	Hot	0	0	nanomole
2	Cold	0	0	nanomole
3	AlbHot	0	0	nanomole
4	AlbCold	0	0	nanomole
5	Albumin	0	0	nanomole

Species in LiverInt

	Species Name	Value	Initial Value	Units
1	Hot	0	0	nanomole
2	Cold	0	0	nanomole

Species in LiverBound

	Species Name	Value	Initial Value	Units
1	Hot	0	0	nanomole
2	Cold	0	0	nanomole

Species in LiverIntern

	Species Name	Value	Initial Value	Units
1	Hot	0	0	nanomole
2	Cold	0	0	nanomole

Species in Prostate

	Species Name	Value	Initial Value	Units
1	R	0	0.59944	nanomole

Species in ProstateVas

	Species Name	Value	Initial Value	Units
1	Hot	0	0	nanomole
2	Cold	0	0	nanomole
3	AlbHot	0	0	nanomole
4	AlbCold	0	0	nanomole
5	Albumin	0	0	nanomole

Species in ProstateInt

	Species Name	Value	Initial Value	Units
1	Hot	0	0	nanomole
2	Cold	0	0	nanomole

Species in ProstateBound

	Species Name	Value	Initial Value	Units
1	Hot	0	0	nanomole
2	Cold	0	0	nanomole

Species in ProstateIntern

	Species Name	Value	Initial Value	Units
1	Hot	0	0	nanomole
2	Cold	0	0	nanomole

Species in GI

	Species Name	Value	Initial Value	Units
1	R	0	2.6682	nanomole

Species in GIVas

	Species Name	Value	Initial Value	Units
1	Hot	0	0	nanomole
2	Cold	0	0	nanomole
3	AlbHot	0	0	nanomole
4	AlbCold	0	0	nanomole

5	Albumin	0	0	nanomole

Species in GIInt

	Species Name	Value	Initial Value	Units
1	Hot	0	0	nanomole
2	Cold	0	0	nanomole

Species in GIBound

	Species Name	Value	Initial Value	Units
1	Hot	0	0	nanomole
2	Cold	0	0	nanomole

Species in GIIntern

	Species Name	Value	Initial Value	Units
1	Hot	0	0	nanomole
2	Cold	0	0	nanomole

Species in RestVas

	Species Name	Value	Initial Value	Units
1	Hot	0	0	nanomole
2	Cold	0	0	nanomole
3	AlbHot	0	0	nanomole
4	AlbCold	0	0	nanomole
5	Albumin	0	0	nanomole

Species in RestInt

	Species Name	Value	Initial Value	Units
1	Hot	0	0	nanomole
2	Cold	0	0	nanomole

Species in SkinVas

	Species Name	Value	Initial Value	Units
1	Hot	0	0	nanomole
2	Cold	0	0	nanomole
3	AlbHot	0	0	nanomole
4	AlbCold	0	0	nanomole
5	Albumin	0	0	nanomole

Species in SkinInt

	Species Name	Value	Initial Value	Units
1	Hot	0	0	nanomole
2	Cold	0	0	nanomole

Species in MuscleVas

	Species Name	Value	Initial Value	Units
1	Hot	0	0	nanomole
2	Cold	0	0	nanomole
3	AlbHot	0	0	nanomole
4	AlbCold	0	0	nanomole
5	Albumin	0	0	nanomole

Species in MuscleInt

	Species Name	Value	Initial Value	Units
1	Hot	0	0	nanomole
2	Cold	0	0	nanomole

Species in BrainVas

	Species Name	Value	Initial Value	Units
1	Hot	0	0	nanomole
2	Cold	0	0	nanomole

3	AlbHot	0	0	nanomole
4	AlbCold	0	0	nanomole
5	Albumin	0	0	nanomole

Species in BrainInt

	Species Name	Value	Initial Value	Units
1	Hot	0	0	nanomole
2	Cold	0	0	nanomole

Species in RedMarrowVas

	Species Name	Value	Initial Value	Units
1	Hot	0	0	nanomole
2	Cold	0	0	nanomole
3	AlbHot	0	0	nanomole
4	AlbCold	0	0	nanomole
5	Albumin	0	0	nanomole

Species in RedMarrowInt

	Species Name	Value	Initial Value	Units
1	Hot	0	0	nanomole
2	Cold	0	0	nanomole

Species in LungsVas

	Species Name	Value	Initial Value	Units
1	Hot	0	0	nanomole
2	Cold	0	0	nanomole
3	AlbHot	0	0	nanomole
4	AlbCold	0	0	nanomole
5	Albumin	0	0	nanomole

Species in LungsInt

	Species Name	Value	Initial Value	Units
1	Hot	0	0	nanomole
2	Cold	0	0	nanomole

Species in AdiposeVas

	Species Name	Value	Initial Value	Units
1	Hot	0	0	nanomole
2	Cold	0	0	nanomole
3	AlbHot	0	0	nanomole
4	AlbCold	0	0	nanomole
5	Albumin	0	0	nanomole

Species in AdiposeInt

	Species Name	Value	Initial Value	Units
1	Hot	0	0	nanomole
2	Cold	0	0	nanomole

Species in KidneyIntera

	Species Name	Value	Initial Value	Units
1	Hot	0	0	nanomole
2	Cold	0	0	nanomole

Species in Data

	Species Name	Value	Initial Value	Units
1	DataTumor1	0	0	nanomole
2	DataTumor2	0	0	nanomole
3	DataSG	0	0	nanomole
4	DataKidney	0	0	nanomole

Model Scoped and Reaction Scoped Parameters

	Parameter Name	Value	Initial Value	Units
1	lambdaPhys	7.15e-05	7.15e-05	1/minute
2	k_on_toAlb	1	0	liter/nanomole/minute
3	k_off_toAlb	1	0	1/minute
4	k_on	0.046	0.046	liter/nanomole/minute
5	k_off	0.368	0.368	1/minute
6	k_on_AlbPharm	0	0	liter/nanomole/minute
7	k_off_AlbPharm	0	0	1/minute
8	F_Tumor1	-1	0.005	liter/minute
9	PSAlb_Tumor1	-1	0	liter/minute
10	PS_Tumor1	-1	0.006	liter/minute
11	Rden_Tumor1	-1	57	nanomole/liter
12	R0_Tumor1	-1	0.57	nanomole
13	lambdaIntern_Tumor1	0.001	0.001	1/minute
14	lambdaRel_Tumor1	-1	0.00015	1/minute
15	F_Tumor2	-1	0.17	liter/minute
16	PSAlb_Tumor2	-1	0	liter/minute
17	PS_Tumor2	-1	0.204	liter/minute
18	Rden_Tumor2	-1	19	nanomole/liter
19	R0_Tumor2	-1	6.46	nanomole
20	lambdaIntern_Tumor2	0.001	0.001	1/minute
21	lambdaRel_Tumor2	-1	0.00015	1/minute
22	F_TumorRest	-1	0.024436	liter/minute
23	PSAlb_TumorRest	-1	0	liter/minute
24	PS_TumorRest	-1	0.029323	liter/minute
25	Rden_TumorRest	266	266	nanomole/liter
26	R0_TumorRest	-1	13	nanomole
27	lambdaIntern_TumorRest	0.001	0.001	1/minute
28	lambdaRel_TumorRest	-1	0.00015	1/minute
29	F_SG	-1	0.001554	liter/minute
30	PS_SG	-1	0.042	liter/minute
31	Rden_SG	-1	38	nanomole/liter
32	R0_SG	-1	0.798	nanomole
33	lambdaIntern_SG	-1	0.001	1/minute
34	lambdaRel_SG	-1	0.00042	1/minute
35	F_Spleen	-1	0.11778	liter/minute
36	PS_Spleen	-1	0.366	liter/minute
37	Rden_Spleen	-1	0.532	nanomole/liter
38	R0_Spleen	-1	0.097356	nanomole
39	lambdaIntern_Spleen	-1	0.001	1/minute
40	lambdaRel_Spleen	-1	0.00029	1/minute
41	F_Prostate	-1	0.0024338	liter/minute
42	PS_Prostate	-1	0.0022535	liter/minute
43	Rden_Prostate	-1	26.6	nanomole/liter
44	R0_Prostate	-1	0.59944	nanomole
45	lambdaIntern_Prostate	-1	0.001	1/minute
46	lambdaRel_Prostate	-1	0.00029	1/minute
47	F_Liver	-1	0.2552	liter/minute
48	PS_Liver	-1	3.82	liter/minute
49	Rden_Liver	-1	1.33	nanomole/liter
50	R0_Liver	-1	2.5403	nanomole
51	lambdaIntern_Liver	-1	0.001	1/minute
52	lambdaRel_Liver	-1	0.00029	1/minute
53	F_GI	-1	0.62819	liter/minute
54	PS_GI	-1	0.033437	liter/minute
55	Rden_GI	-1	1.596	nanomole/liter
[illegible]	[illegible]	[illegible]	[illegible]	[illegible]

56	R0_GI	-1	2.6682	nanomole
57	lambdaIntern_GI	-1	0.001	1/minute
58	lambdaRel_GI	-1	0.00029	1/minute
59	F_Rest	-1	0.17269	liter/minute
60	PS_Rest	-1	0.23009	liter/minute
61	F_Muscle	-1	0.66745	liter/minute
62	PS_Muscle	-1	0.84727	liter/minute
63	F_Skin	-1	0.19631	liter/minute
64	PS_Skin	-1	0.096	liter/minute
65	F_RedMarrow	-1	0.11778	liter/minute
66	PS_RedMarrow	-1	3.0986	liter/minute
67	F_Brain	-1	0.47114	liter/minute
68	PS_Brain	-1	0	liter/minute
69	F_Adipose	-1	0.19631	liter/minute
70	PS_Adipose	-1	0.3793	liter/minute
71	F_Bone	-1	0.19631	liter/minute
72	PS_Bone	-1	0.25535	liter/minute
73	F_Heart	-1	0.15705	liter/minute
74	PS_Heart	-1	0.0096056	liter/minute
75	F_Lungs	-1	3.9262	liter/minute
76	PS_Lungs	-1	2.8169	liter/minute
77	F_Kidney	-1	0.74597	liter/minute
78	TER_Kidney	-1	0.2	liter/minute
79	Rden_Kidney	-1	14	nanomole/liter
80	R0_Kidney	-1	4.354	nanomole
81	lambdaIntern_Kidney	-1	0.001	1/minute
82	lambdaRel_Kidney	-1	0.00029	1/minute
83	GFR	-1	0.088742	liter/minute
84	phi	0.66	0.66	dimensionless
85	f_exc	0.96	0.96	dimensionless
86	lambdaIntern_Normal	-1	0.001	1/minute
87	lambdaRel_Normal	-1	0.00029	1/minute
88	density	1	1	kilogram/liter
89	bodySurfaceArea	-1	1.9	meter^2
90	hematocrit	0.4	0.4	dimensionless
91	bodyHeight	-1	160	meter
92	bodyWeight	-1	100	kilogram
93	F	-1	3.9262	liter/minute
94	V_p	-1	3.192	liter
95	kPS_Tumor1	0.6	0.6	1/minute
96	kPS_Tumor2	0.6	0.6	1/minute
97	kPS_TumorRest	0.6	0.6	1/minute
98	kPS_SG	-1	2	1/minute
99	kPS_Spleen	-1	2	1/minute
100	kPS_Prostate	-1	0.1	1/minute
101	kPS_Liver	-1	2	1/minute
102	kPS_Rest	-1	0.02	1/minute
103	kPS_GI	-1	0.02	1/minute
104	kPS_Muscle	0.02	0.02	1/minute
105	kPS_Skin	-1	0.02	1/minute
106	kPS_Brain	0	0	1/minute
107	kPS_RedMarrow	-1	2	1/minute
108	kPS_Adipose	-1	0.02	1/minute
109	kPS_Bone	-1	0.02	1/minute
110	kPS_Heart	-1	0.02	1/minute
111	kPS_Lungs	-1	2	1/minute
112	kPSAlb_Tumor1	-1	0	1/minute

113	kPSAlb_Tumor2	-1	0	1/minute
114	kPSAlb_TumorRest	-1	0	1/minute
115	F_Total	1	4.1256	liter/minute
116	f_SG	1	0.074	1/minute
117	f_Prostate	1	0.108	1/minute
118	f_Tumor1	0.5	0.5	1/minute
119	f_Tumor2	0.5	0.5	1/minute
120	HotTotalAmountOriginal	10	10	nanomole
121	ColdTotalAmountOriginal	100	100	nanomole
122	RepeatTimeInterval	100	100	minute
123	RepeatCount	0	0	dimensionless
124	PerInjectionRate	100	100	nanomole/minute
125	HotPerInjection	0	20	nanomole
126	ColdPerInjection	0	200	nanomole
127	dMin	1	1	minute
128	dLiter	1	1	liter
129	dMeter	1	1	meter
130	dLiterKg	1	1	liter/kilogram
131	numberPerNanomole	602200000000000	602200000000000	dimensionless
132	Tumor1VolumeCoeff	1	1	dimensionless
133	Tumor1Volume	-1	0.01	liter
134	Albumin_0	-1	0	nanomole
135	AlbuminDen	-1	0	nanomole/liter
136	K_D_Alb	-1	1	nanomole/liter
137	injectionCoeff	2	2	dimensionless
138	HotTotalAmount	1	20	nanomole
139	ColdTotalAmount	1	200	nanomole
140	Rden_Tumor1_Coeff	1	1	dimensionless

Initial Assignments

	Initial Assignments	Initial Value
1	PS_Tumor1 = kPS_Tumor1 * Tumor1	0.006
2	PSAlb_Tumor1 = kPSAlb_Tumor1 * Tumor1	0
3	PS_Tumor2 = kPS_Tumor2 * Tumor2	0.204
4	PSAlb_Tumor2 = kPSAlb_Tumor2 * Tumor2	0
5	PS_TumorRest = kPS_TumorRest * TumorRest	0.029323
6	PSAlb_TumorRest = kPSAlb_TumorRest * TumorRest	0
7	PS_SG = kPS_SG * SG	0.042
8	PS_Liver = kPS_Liver * Liver	3.82
9	PS_Spleen = kPS_Spleen * Spleen	0.366
10	PS_Prostate = kPS_Prostate * Prostate	0.0022535
11	PS_GI = kPS_GI * GI	0.033437
12	PS_Rest = kPS_Rest * Rest	0.23009
13	PS_Muscle = kPS_Muscle * Muscle	0.84727
14	PS_RedMarrow = kPS_RedMarrow*RedMarrow	3.0986
15	PS_Adipose = kPS_Adipose * Adipose	0.3793
16	PS_Bone = kPS_Bone * Bone	0.25535
17	PS_Heart = kPS_Heart * Heart	0.0096056
18	PS_Lungs = kPS_Lungs * Lungs	2.8169
19	PS_Brain = kPS_Brain * Brain	0
20	PS_Skin = kPS_Skin * Skin	0.096
21	F_Muscle = 0.17 * F	0.66745
22	F_GI = 0.16 * F	0.62819
23	F_Skin = 0.05 * F	0.19631
24	F_Adipose = 0.05 * F	0.19631
25	F_RedMarrow = 0.03 * F	0.11778
26	F_Bone = 0.05 * F	0.19631

27	F_Heart = 0.04 * F	0.15705
28	F_Brain = 0.12 * F	0.47114
29	F_Rest = F - (F_Brain+F_Heart+F_Bone+F_RedMarrow+F_Adipose+F_Skin+F_GI+F_Muscle + F_Liver + F_Kidney + F_Spleen + F_Prostate + F_SG)	0.17269
30	F_Total = F + F_Tumor1 + F_Tumor2 + F_TumorRest	4.1256
31	F_Prostate = f_Prostate * Prostate	0.0024338
32	F_SG = f_SG * SG	0.001554
33	F_Liver = 0.065 * F	0.2552
34	F_Spleen = 0.03 * F	0.11778
35	F_Kidney = 0.19 * F	0.74597
36	F_Tumor1 = f_Tumor1 * Tumor1	0.005
37	F_Tumor2 = f_Tumor2 * Tumor2	0.17
38	F_TumorRest = f_Tumor1 * R0_TumorRest / Rden_TumorRest	0.024436
39	Tumor1Vas = 0.05 * (1-hematocrit) * Tumor1	0.0003
40	Tumor1Int = 0.38 * Tumor1	0.0038
41	Tumor2Vas = 0.05 * (1-hematocrit) * Tumor2	0.0102
42	Tumor2Int = 0.38 * Tumor2	0.1292
43	TumorRest = R0_TumorRest / Rden_TumorRest	0.048872
44	TumorRestVas = 0.05 * (1-hematocrit) * TumorRest	0.0014662
45	TumorRestInt = 0.38 * TumorRest	0.018571
46	Spleen = 0.183	0.183
47	SpleenVas = 0.12 * Spleen	0.02196
48	SpleenInt = 0.2 * Spleen	0.0366
49	SGVas = 0.03* (1-hematocrit) * SG	0.000378
50	SGInt = 0.23 * SG	0.00483
51	Liver = 1.91	1.91
52	LiverVas = 0.085 * Liver	0.16235
53	LiverInt = 0.2 * Liver	0.382
54	Prostate = 0.016 * bodyWeight / 71 * dLiterKg	0.022535
55	ProstateVas = 0.004 * (1-hematocrit) * Prostate	5.4085e-05
56	ProstateInt = 0.25 * Prostate	0.0056338
57	GI = (0.385 + 0.548 + 0.104 + 0.15)*bodyWeight/71 * dLiterKg	1.6718
58	GIInt = 8.8 * GIVas	2.1348
59	GIVas = 0.076 * V_p	0.24259
60	Rest = bodyWeight *dLiterKg -(Lungs + Brain+Heart+Bone+RedMarrow+Adipose+Skin+GI+Muscle + Liver + Kidney + Spleen + Prostate + SG)	11.5046
61	RestInt = 4.1 * RestVas	5.8076
62	RestVas = V_p -(LungsVas + BrainVas+HeartVas+BoneVas+RedMarrowVas+AdiposeVas+SkinVas+GIVas+MuscleVas + LiverVas + KidneyVas + SpleenVas + ProstateVas + SGVas)	1.4165
63	Muscle = 30.078 * bodyWeight/71 * dLiterKg	42.3634
64	MuscleVas = 0.14 * V_p	0.44688
65	MuscleInt = 5.9 * MuscleVas	2.6366
66	Skin = 3.408 * bodyWeight / 71 * dLiterKg	4.8
67	SkinVas = 0.03 * V_p	0.09576
68	SkinInt = 8.9 * SkinVas	0.85226
69	Brain = 1.45 * bodyWeight / 71 * dLiterKg	2.0423
70	BrainVas = 0.012 * V_p	0.038304
71	BrainInt = 1 * dLiter	1
72	Lungs = 1 * bodyWeight / 71 * dLiterKg	1.4085
73	LungsVas = 0.105 * V_p	0.33516
74	LungsInt = 5.5 * LungsVas	1.8434
75	Heart = 0.341 * bodyWeight / 71 * dLiterKg	0.48028
76	HeartVas = 0.01 * V_p	0.03192
77	HeartInt = 3.7 * HeartVas	0.1181
78	Bone = 10.165 * bodyWeight /71 * dLiterKg - RedMarrow	12.7676

79	BoneVas = 0.07*V_p - RedMarrowVas	0.09576	
80	BoneInt = 8.4 * BoneVas	0.80438	
81	Adipose = 13.465 * bodyWeight / 71 * dLiterKg	18.9648	
82	AdiposeVas = 0.05 * V_p	0.1596	
83	AdiposeInt = 15.5 * AdiposeVas	2.4738	
84	RedMarrow = 1.1 * bodyWeight/71 * dLiterKg	1.5493	
85	RedMarrowVas = 0.04 * V_p	0.12768	
86	RedMarrowInt = 3.7 * RedMarrowVas	0.47242	
87	KidneyIntera = (Kidney - KidneyInt - KidneyVas) * 2/3	0.16483	
88	KidneyVas = 0.055 * Kidney	0.017105	
89	KidneyInt = 0.15 * Kidney	0.04665	
90	R0_Tumor1 = Rden_Tumor1 * Tumor1	0.57	
91	R0_Tumor2 = Rden_Tumor2 * Tumor2	6.46	
92	R0_Kidney = Rden_Kidney*Kidney	4.354	
93	R0_Spleen = Rden_Spleen * Spleen	0.097356	
94	R0_Prostate = Rden_Prostate * Prostate	0.59944	
95	R0_GI = Rden_GI * GI	2.6682	
96	R0_SG = Rden_SG * SG	0.798	
97	R0_Liver = Rden_Liver * Liver	2.5403	
98	lambdaRel_TumorRest = (lambdaRel_Tumor1 + lambdaRel_Tumor2)/2	0.00015	
99	lambdaIntern_SG = lambdaIntern_Normal	0.001	
100	lambdaIntern_Liver = lambdaIntern_Tumor1	0.001	
101	lambdaIntern_Spleen = lambdaIntern_Tumor1	0.001	
102	lambdaRel_Liver = lambdaRel_Kidney	0.00029	
103	lambdaRel_Spleen = lambdaRel_Kidney	0.00029	
104	lambdaIntern_Prostate = lambdaIntern_Normal	0.001	
105	lambdaIntern_GI = lambdaIntern_Normal	0.001	
106	lambdaRel_Prostate = lambdaRel_Normal	0.00029	
107	lambdaRel_GI = lambdaRel_Normal	0.00029	
108	lambdaRel_Normal = lambdaRel_Kidney	0.00029	
109	lambdaIntern_Normal = lambdaIntern_Tumor1	0.001	
110	Rden_Spleen = 0.02 * Rden_Prostate	0.532	
111	Rden_Liver = 0.05 * Rden_Prostate	1.33	
112	Rden_Prostate = 0.1 * Rden_TumorRest	26.6	
113	Rden_GI = 0.06 * Rden_Prostate	1.596	
114	F_Lungs = F	3.9262	
115	GFR = TER_Kidney /3 * 201/151	0.088742	
116	F = V_p*1.23/dMin	3.9262	
117	V_p = 2.8 * (1-hematocrit) * bodySurfaceArea * dLiter / dMeter^2	3.192	
118	kPS_Liver = kPS_Muscle * 100	2	
119	kPS_Spleen = kPS_Liver	2	
120	kPS_Lungs = kPS_Muscle * 100	2	
121	kPS_SG = kPS_Muscle * 100	2	
122	kPS_GI = kPS_Muscle	0.02	
123	kPS_Skin = kPS_Muscle	0.02	
124	kPS_Adipose = kPS_Muscle	0.02	
125	kPS_RedMarrow = kPS_Liver	2	
126	kPS_Heart = kPS_Muscle	0.02	
127	kPS_Bone = kPS_Muscle	0.02	
128	kPS_Rest = kPS_Muscle	0.02	
129	f_Prostate = 0.18 * (1-hematocrit) / dMin	0.108	
130	HotPerInjection = HotTotalAmount/(RepeatCount+1)	20	
131	ColdPerInjection = ColdTotalAmount/(RepeatCount+1)	200	
132	lambdaIntern_Kidney = lambdaIntern_Tumor1	0.001	
133	kPS_Prostate = 0.1	0.1	
134	Tumor1 = Tumor1Volume * Tumor1VolumeCoeff	0.01	

135	Albumin_0 = AlbuminDen*V_p	0
136	Art = 0.06*V_p + 0.045*V_p	0.33516
137	Vein = 0.18*V_p + 0.045*V_p	0.7182
138	Vein.Albumin = AlbuminDen * Vein	0
139	Art.Albumin = AlbuminDen * Art	0
140	Tumor1Vas.Albumin = AlbuminDen* Tumor1Vas	0
141	Tumor1Int.Albumin = AlbuminDen * Tumor1Int	0
142	Tumor2Vas.Albumin = AlbuminDen * Tumor2Vas	0
143	Tumor2Int.Albumin = AlbuminDen * Tumor2Int	0
144	TumorRestVas.Albumin = AlbuminDen * TumorRestVas	0
145	TumorRestInt.Albumin = AlbuminDen * TumorRestInt	0
146	KidneyVas.Albumin = AlbuminDen * KidneyVas	0
147	SpleenVas.Albumin = AlbuminDen * SpleenVas	0
148	ProstateVas.Albumin = AlbuminDen * ProstateVas	0
149	GIVas.Albumin = AlbuminDen * GIVas	0
150	SkinVas.Albumin = AlbuminDen * SkinVas	0
151	BrainVas.Albumin = AlbuminDen * BrainVas	0
152	LungsVas.Albumin = AlbuminDen * LungsVas	0
153	HeartVas.Albumin = AlbuminDen * HeartVas	0
154	BoneVas.Albumin = AlbuminDen * BoneVas	0
155	AdiposeVas.Albumin = AlbuminDen * AdiposeVas	0
156	RedMarrowVas.Albumin = AlbuminDen * RedMarrowVas	0
157	MuscleVas.Albumin = AlbuminDen * MuscleVas	0
158	RestVas.Albumin = AlbuminDen * RestVas	0
159	LiverVas.Albumin = AlbuminDen * LiverVas	0
160	SGVas.Albumin = AlbuminDen * SGVas	0
161	k_on_toAlb = k_off_toAlb / K_D_Alb	0
162	K_D_Alb = k_off_toAlb / k_on_toAlb	1
163	HotTotalAmount = injectionCoeff * HotTotalAmountOriginal	20
164	ColdTotalAmount = injectionCoeff * ColdTotalAmountOriginal	200

Repeated Assignments

	Repeated Assignments	Initial Value
1	Tumor2.R = R0_Tumor2 - Tumor2Bound.Hot - Tumor2Bound.Cold	6.46
2	Tumor1.R = R0_Tumor1 - Tumor1Bound.Hot - Tumor1Bound.Cold	0.57
3	Kidney.R = R0_Kidney-KidneyBound.Hot-KidneyBound.Cold	4.354
4	SG.R = R0_SG - SGBound.Hot - SGBound.Cold	0.798
5	TumorRest.R = R0_TumorRest - TumorRestBound.Hot - TumorRestBound.Cold	13
6	Spleen.R = R0_Spleen - SpleenBound.Hot - SpleenBound.Cold	0.097356
7	Liver.R = R0_Liver - LiverBound.Hot - LiverBound.Cold	2.5403
8	Prostate.R = R0_Prostate - ProstateBound.Hot - ProstateBound.Cold	0.59944
9	GI.R = R0_GI - GIBound.Hot - GIBound.Cold	2.6682
10	Data.DataKidney = KidneyVas.Hot + KidneyInt.Hot + KidneyBound.Hot + KidneyIntern.Hot	0

Reactions

	Reactions
1	Tumor2Vas.Hot -> Tumor2Vas.Cold
	*lambdaPhys*Tumor2Vas.Hot*
2	Tumor2Vas.Hot + Tumor2Vas.Albumin <-> Tumor2Vas.AlbHot
	*k_on_toAlb*Tumor2Vas.Hot*Tumor2Vas.Albumin/Tumor2Vas-k_off_toAlb*Tumor2Vas.AlbHot*
3	Tumor2Vas.AlbHot -> Tumor2Vas.AlbCold
	*lambdaPhys*Tumor2Vas.AlbHot*
4	Tumor2Vas.Cold + Tumor2Vas.Albumin <-> Tumor2Vas.AlbCold
	*k_on_toAlb*Tumor2Vas.Cold*Tumor2Vas.Albumin/Tumor2Vas-k_off_toAlb*Tumor2Vas.AlbCold*
5	Tumor2Vas.Hot <-> Tumor2Int.Hot
	*PS_Tumor2/Tumor2Vas*Tumor2Vas.Hot - PS_Tumor2/Tumor2Int*Tumor2Int.Hot*

6	Tumor2Vas.AlbHot -> Tumor2Int.AlbHot
	PSAlb_Tumor2/Tumor2Vas*Tumor2Vas.AlbHot
7	Tumor2Vas.AlbCold -> Tumor2Int.AlbCold
	PSAlb_Tumor2/Tumor2Vas*Tumor2Vas.AlbCold
8	Tumor2Vas.Cold -> Tumor2Int.Cold
	PS_Tumor2/Tumor2Vas*Tumor2Vas.Cold - PS_Tumor2/Tumor2Int*Tumor2Int.Cold
9	Tumor2Int.Hot <-> Tumor2Bound.Hot
	k_on/Tumor2Int*Tumor2Int.Hot*Tumor2.R - k_off*Tumor2Bound.Hot
10	Tumor2Int.Cold <-> Tumor2Bound.Cold
	k_on/Tumor2Int*Tumor2Int.Cold*Tumor2.R - k_off*Tumor2Bound.Cold
11	Tumor2Bound.Hot -> Tumor2Intern.Hot
	lambdaIntern_Tumor2*Tumor2Bound.Hot
12	Tumor2Bound.Cold -> Tumor2Intern.Cold
	lambdaIntern_Tumor2*Tumor2Bound.Cold
13	Art.Hot -> Tumor2Vas.Hot
	F_Tumor2/Art*Art.Hot
14	Art.AlbHot -> Tumor2Vas.AlbHot
	F_Tumor2/Art*Art.AlbHot
15	Art.AlbCold -> Tumor2Vas.AlbCold
	F_Tumor2/Art*Art.AlbCold
16	Art.Cold -> Tumor2Vas.Cold
	F_Tumor2/Art*Art.Cold
17	Tumor2Vas.Hot -> Vein.Hot
	F_Tumor2/Tumor2Vas*Tumor2Vas.Hot
18	Tumor2Vas.AlbHot -> Vein.AlbHot
	F_Tumor2/Tumor2Vas*Tumor2Vas.AlbHot
19	Tumor2Vas.AlbCold -> Vein.AlbCold
	F_Tumor2/Tumor2Vas*Tumor2Vas.AlbCold
20	Tumor2Vas.Cold -> Vein.Cold
	F_Tumor2/Tumor2Vas*Tumor2Vas.Cold
21	Tumor2Int.Hot -> Tumor2Int.Cold
	lambdaPhys*Tumor2Int.Hot
22	Tumor2Int.Hot + Tumor2Int.Albumin <-> Tumor2Int.AlbHot
	k_on_toAlb*Tumor2Int.Hot*Tumor2Int.Albumin/Tumor2Int-k_off_toAlb*Tumor2Int.AlbHot
23	Tumor2Int.AlbHot -> Tumor2Int.AlbCold
	lambdaPhys*Tumor2Int.AlbHot
24	Tumor2Int.Cold + Tumor2Int.Albumin <-> Tumor2Int.AlbCold
	k_on_toAlb*Tumor2Int.Cold*Tumor2Int.Albumin/Tumor2Int-k_off_toAlb*Tumor2Int.AlbCold
25	Tumor2Bound.Hot -> Tumor2Bound.Cold
	lambdaPhys*Tumor2Bound.Hot
26	Tumor2Intern.Hot -> Tumor2Intern.Cold
	lambdaPhys*Tumor2Intern.Hot
27	Tumor2Intern.Hot -> null
	lambdaRel_Tumor2*Tumor2Intern.Hot
28	Tumor2Intern.Cold -> null
	lambdaRel_Tumor2*Tumor2Intern.Cold
29	Art.Hot -> Art.Cold
	lambdaPhys*Art.Hot
30	Art.Hot + Art.Albumin <-> Art.AlbHot
	k_on_toAlb*Art.Hot*Art.Albumin/Art-k_off_toAlb*Art.AlbHot
31	Art.AlbHot -> Art.AlbCold
	lambdaPhys*Art.AlbHot
32	Art.Cold + Art.Albumin <-> Art.AlbCold
	k_on_toAlb*Art.Cold*Art.Albumin/Art-k_off_toAlb*Art.AlbCold
33	Vein.Hot -> Vein.Cold
	lambdaPhys*Vein.Hot
34	Vein.Hot + Vein.Albumin <-> Vein.AlbHot

	k_on_toAlb*Vein.Hot*Vein.Albumin/Vein-k_off_toAlb*Vein.AlbHot
35	Vein.AlbHot -> Vein.AlbCold
	lambdaPhys*Vein.AlbHot
36	Vein.Cold + Vein.Albumin <-> Vein.AlbCold
	k_on_toAlb*Vein.Cold*Vein.Albumin/Vein-k_off_toAlb*Vein.AlbCold
37	Tumor1Vas.Hot -> Tumor1Vas.Cold
	lambdaPhys*Tumor1Vas.Hot
38	Tumor1Vas.Hot + Tumor1Vas.Albumin <-> Tumor1Vas.AlbHot
	k_on_toAlb*Tumor1Vas.Hot*Tumor1Vas.Albumin/Tumor1Vas-k_off_toAlb*Tumor1Vas.AlbHot
39	Tumor1Vas.AlbHot -> Tumor1Vas.AlbCold
	lambdaPhys*Tumor1Vas.AlbHot
40	Tumor1Vas.Cold + Tumor1Vas.Albumin <-> Tumor1Vas.AlbCold
	k_on_toAlb*Tumor1Vas.Cold*Tumor1Vas.Albumin/Tumor1Vas-k_off_toAlb*Tumor1Vas.AlbCold
41	Tumor1Vas.Hot <-> Tumor1Int.Hot
	PS_Tumor1/Tumor1Vas*Tumor1Vas.Hot - PS_Tumor1/Tumor1Int*Tumor1Int.Hot
42	Tumor1Vas.AlbHot <-> Tumor1Int.AlbHot
	PSAlb_Tumor1/Tumor1Vas*Tumor1Vas.AlbHot - PSAlb_Tumor1/Tumor1Int*Tumor1Int.AlbHot
43	Tumor1Vas.AlbCold -> Tumor1Int.AlbCold
	PSAlb_Tumor1/Tumor1Vas*Tumor1Vas.AlbCold - PSAlb_Tumor1/Tumor1Int * Tumor1Int.AlbCold
44	Tumor1Vas.Cold <-> Tumor1Int.Cold
	PS_Tumor1/Tumor1Vas*Tumor1Vas.Cold - PS_Tumor1/Tumor1Int*Tumor1Int.Cold
45	Tumor1Int.Hot <-> Tumor1Bound.Hot
	k_on/Tumor1Int*Tumor1Int.Hot*Tumor1.R - k_off*Tumor1Bound.Hot
46	Tumor1Int.Cold <-> Tumor1Bound.Cold
	k_on/Tumor1Int*Tumor1.R*Tumor1Int.Cold - k_off*Tumor1Bound.Cold
47	Tumor1Bound.Hot -> Tumor1Intern.Hot
	lambdaIntern_Tumor1*Tumor1Bound.Hot
48	Tumor1Bound.Cold -> Tumor1Intern.Cold
	lambdaIntern_Tumor1*Tumor1Bound.Cold
49	Art.Hot -> Tumor1Vas.Hot
	F_Tumor1/Art*Art.Hot
50	Art.AlbHot -> Tumor1Vas.AlbHot
	F_Tumor1/Art*Art.AlbHot
51	Art.AlbCold -> Tumor1Vas.AlbCold
	F_Tumor1/Art*Art.AlbCold
52	Art.Cold -> Tumor1Vas.Cold
	F_Tumor1/Art*Art.Cold
53	Tumor1Vas.Hot -> Vein.Hot
	F_Tumor1/Tumor1Vas*Tumor1Vas.Hot
54	Tumor1Vas.AlbHot -> Vein.AlbHot
	F_Tumor1/Tumor1Vas*Tumor1Vas.AlbHot
55	Tumor1Vas.AlbCold -> Vein.AlbCold
	F_Tumor1/Tumor1Vas*Tumor1Vas.AlbCold
56	Tumor1Vas.Cold -> Vein.Cold
	F_Tumor1/Tumor1Vas*Tumor1Vas.Cold
57	Tumor1Int.Hot -> Tumor1Int.Cold
	lambdaPhys*Tumor1Int.Hot
58	Tumor1Int.Hot + Tumor1Int.Albumin -> Tumor1Int.AlbHot
	k_on_toAlb*Tumor1Int.Hot*Tumor1Int.Albumin/Tumor1Int-k_off_toAlb*Tumor1Int.AlbHot
59	Tumor1Int.AlbHot -> Tumor1Int.AlbCold
	lambdaPhys*Tumor1Int.AlbHot
60	Tumor1Int.Cold + Tumor1Int.Albumin <-> Tumor1Int.AlbCold
	k_on_toAlb*Tumor1Int.Cold*Tumor1Int.Albumin/Tumor1Int-k_off_toAlb*Tumor1Int.AlbCold
61	Tumor1Bound.Hot -> Tumor1Bound.Cold
	lambdaPhys*Tumor1Bound.Hot
62	Tumor1Intern.Hot -> Tumor1Intern.Cold
	lambdaPhys*Tumor1Intern.Hot

63	Tumor1Intern.Hot -> null
	*lambdaRel_Tumor1*Tumor1Intern.Hot*
64	Tumor1Intern.Cold -> null
	*lambdaRel_Tumor1*Tumor1Intern.Cold*
65	KidneyVas.Hot -> KidneyVas.Cold
	*lambdaPhys*KidneyVas.Hot*
66	KidneyVas.Hot + KidneyVas.Albumin <-> KidneyVas.AlbHot
	*k_on_toAlb*KidneyVas.Hot*KidneyVas.Albumin/KidneyVas-k_off_toAlb*KidneyVas.AlbHot*
67	KidneyVas.AlbHot -> KidneyVas.AlbCold
	*lambdaPhys*KidneyVas.AlbHot*
68	KidneyVas.Cold + KidneyVas.Albumin <-> KidneyVas.AlbCold
	*k_on_toAlb*KidneyVas.Cold*KidneyVas.Albumin/KidneyVas-k_off_toAlb*KidneyVas.AlbCold*
69	KidneyInt.Hot <-> KidneyBound.Hot
	*k_on/KidneyInt*KidneyInt.Hot*Kidney.R - k_off*KidneyBound.Hot*
70	KidneyInt.Cold <-> KidneyBound.Cold
	*k_on/KidneyInt*KidneyInt.Cold*Kidney.R - k_off*KidneyBound.Cold*
71	KidneyBound.Hot -> KidneyIntern.Hot
	*lambdaIntern_Kidney*KidneyBound.Hot*
72	KidneyBound.Cold -> KidneyIntern.Cold
	*lambdaIntern_Kidney*KidneyBound.Cold*
73	Art.Hot -> KidneyVas.Hot
	*F_Kidney/Art*Art.Hot*
74	Art.AlbHot -> KidneyVas.AlbHot
	*F_Kidney/Art*Art.AlbHot*
75	Art.AlbCold -> KidneyVas.AlbCold
	*F_Kidney/Art*Art.AlbCold*
76	Art.Cold -> KidneyVas.Cold
	*F_Kidney/Art*Art.Cold*
77	KidneyVas.Hot -> Vein.Hot
	*F_Kidney/KidneyVas*KidneyVas.Hot*
78	KidneyVas.AlbHot -> Vein.AlbHot
	*F_Kidney/KidneyVas*KidneyVas.AlbHot*
79	KidneyVas.AlbCold -> Vein.AlbCold
	*F_Kidney/KidneyVas*KidneyVas.AlbCold*
80	KidneyVas.Cold -> Vein.Cold
	*F_Kidney/KidneyVas*KidneyVas.Cold*
81	KidneyInt.Hot -> KidneyInt.Cold
	*lambdaPhys*KidneyInt.Hot*
82	KidneyBound.Hot -> KidneyBound.Cold
	*lambdaPhys*KidneyBound.Hot*
83	KidneyIntern.Hot -> KidneyIntern.Cold
	*lambdaPhys*KidneyIntern.Hot*
84	KidneyIntern.Hot -> null
	*lambdaRel_Kidney*KidneyIntern.Hot*
85	KidneyIntern.Cold -> null
	*lambdaRel_Kidney*KidneyIntern.Cold*
86	HeartVas.Hot -> HeartVas.Cold
	*lambdaPhys*HeartVas.Hot*
87	HeartVas.Hot + HeartVas.Albumin <-> HeartVas.AlbHot
	*k_on_toAlb*HeartVas.Hot*HeartVas.Albumin/HeartVas-k_off_toAlb*HeartVas.AlbHot*
88	HeartVas.AlbHot -> HeartVas.AlbCold
	*lambdaPhys*HeartVas.AlbHot*
89	HeartVas.Cold + HeartVas.Albumin <-> HeartVas.AlbCold
	*k_on_toAlb*HeartVas.Cold*HeartVas.Albumin/HeartVas-k_off_toAlb*HeartVas.AlbCold*
90	HeartVas.Hot <-> HeartInt.Hot
	*PS_Heart/HeartVas*HeartVas.Hot - PS_Heart/HeartInt*HeartInt.Hot*

91	HeartVas.Cold <-> HeartInt.Cold
	PS_Heart/HeartVas*HeartVas.Cold - PS_Heart/HeartInt*HeartInt.Cold
92	Art.Hot -> HeartVas.Hot
	F_Heart/Art*Art.Hot
93	Art.AlbHot -> HeartVas.AlbHot
	F_Heart/Art*Art.AlbHot
94	Art.AlbCold -> HeartVas.AlbCold
	F_Heart/Art*Art.AlbCold
95	Art.Cold -> HeartVas.Cold
	F_Heart/Art*Art.Cold
96	HeartVas.Hot -> Vein.Hot
	F_Heart/HeartVas*HeartVas.Hot
97	HeartVas.AlbHot -> Vein.AlbHot
	F_Heart/HeartVas*HeartVas.AlbHot
98	HeartVas.AlbCold -> Vein.AlbCold
	F_Heart/HeartVas*HeartVas.AlbCold
99	HeartVas.Cold -> Vein.Cold
	F_Heart/HeartVas*HeartVas.Cold
100	HeartInt.Hot -> HeartInt.Cold
	lambdaPhys*HeartInt.Hot
101	SGVas.Hot -> SGVas.Cold
	lambdaPhys*SGVas.Hot
102	SGVas.Hot + SGVas.Albumin <-> SGVas.AlbHot
	k_on_toAlb*SGVas.Hot*SGVas.Albumin/SGVas-k_off_toAlb*SGVas.AlbHot
103	SGVas.AlbHot -> SGVas.AlbCold
	lambdaPhys*SGVas.AlbHot
104	SGVas.Cold + SGVas.Albumin <-> SGVas.AlbCold
	k_on_toAlb*SGVas.Cold*SGVas.Albumin/SGVas-k_off_toAlb*SGVas.AlbCold
105	SGVas.Hot <-> SGInt.Hot
	PS_SG/SGVas*SGVas.Hot - PS_SG/SGInt*SGInt.Hot
106	SGVas.Cold <-> SGInt.Cold
	PS_SG/SGVas*SGVas.Cold - PS_SG/SGInt*SGInt.Cold
107	SGInt.Hot <-> SGBound.Hot
	k_on/SGInt*SGInt.Hot*SG.R - k_off*SGBound.Hot
108	SGInt.Cold <-> SGBound.Cold
	k_on/SGInt*SGInt.Cold*SG.R - k_off*SGBound.Cold
109	SGBound.Hot -> SGIntern.Hot
	lambdaIntern_SG*SGBound.Hot
110	SGBound.Cold -> SGIntern.Cold
	lambdaIntern_SG*SGBound.Cold
111	Art.Hot -> SGVas.Hot
	F_SG/Art*Art.Hot
112	Art.AlbHot -> SGVas.AlbHot
	F_SG/Art*Art.AlbHot
113	Art.AlbCold -> SGVas.AlbCold
	F_SG/Art*Art.AlbCold
114	Art.Cold -> SGVas.Cold
	F_SG/Art*Art.Cold
115	SGVas.Hot -> Vein.Hot
	F_SG/SGVas*SGVas.Hot
116	SGVas.AlbHot -> Vein.AlbHot
	F_SG/SGVas*SGVas.AlbHot
117	SGVas.AlbCold -> Vein.AlbCold
	F_SG/SGVas*SGVas.AlbCold
118	SGVas.Cold -> Vein.Cold
	F_SG/SGVas*SGVas.Cold
119	SGInt.Hot -> SGInt.Cold

	lambdaPhys*SGInt.Hot
120	SGBound.Hot -> SGBound.Cold
	lambdaPhys*SGBound.Hot
121	SGIntern.Hot -> SGIntern.Cold
	lambdaPhys*SGIntern.Hot
122	SGIntern.Hot -> null
	lambdaRel_SG*SGIntern.Hot
123	SGIntern.Cold -> null
	lambdaRel_SG*SGIntern.Cold
124	BoneVas.Hot -> BoneVas.Cold
	lambdaPhys*BoneVas.Hot
125	BoneVas.Hot + BoneVas.Albumin <-> BoneVas.AlbHot
	k_on_toAlb*BoneVas.Hot*BoneVas.Albumin/BoneVas-k_off_toAlb*BoneVas.AlbHot
126	BoneVas.AlbHot -> BoneVas.AlbCold
	lambdaPhys*BoneVas.AlbHot
127	BoneVas.Cold + BoneVas.Albumin <-> BoneVas.AlbCold
	k_on_toAlb*BoneVas.Cold*BoneVas.Albumin/BoneVas-k_off_toAlb*BoneVas.AlbCold
128	BoneVas.Hot <-> BoneInt.Hot
	PS_Bone/BoneVas*BoneVas.Hot-PS_Bone/BoneInt*BoneInt.Hot
129	BoneVas.Cold <-> BoneInt.Cold
	PS_Bone/BoneVas*BoneVas.Cold - PS_Bone/BoneInt*BoneInt.Cold
130	Art.Hot -> BoneVas.Hot
	F_Bone/Art*Art.Hot
131	Art.AlbHot -> BoneVas.AlbHot
	F_Bone/Art*Art.AlbHot
132	Art.AlbCold -> BoneVas.AlbCold
	F_Bone/Art*Art.AlbCold
133	Art.Cold -> BoneVas.Cold
	F_Bone/Art*Art.Cold
134	BoneVas.Hot -> Vein.Hot
	F_Bone/BoneVas*BoneVas.Hot
135	BoneVas.AlbHot -> Vein.AlbHot
	F_Bone/BoneVas*BoneVas.AlbHot
136	BoneVas.AlbCold -> Vein.AlbCold
	F_Bone/BoneVas*BoneVas.AlbCold
137	BoneVas.Cold -> Vein.Cold
	F_Bone/BoneVas*BoneVas.Cold
138	BoneInt.Hot -> BoneInt.Cold
	lambdaPhys*BoneInt.Hot
139	TumorRestVas.Hot -> TumorRestVas.Cold
	lambdaPhys*TumorRestVas.Hot
140	TumorRestVas.Hot + TumorRestVas.Albumin <-> TumorRestVas.AlbHot
	k_on_toAlb*TumorRestVas.Hot*TumorRestVas.Albumin/TumorRestVas-k_off_toAlb*TumorRestVas.AlbHot
141	TumorRestVas.AlbHot -> TumorRestVas.AlbCold
	lambdaPhys*TumorRestVas.AlbHot
142	TumorRestVas.Cold + TumorRestVas.Albumin <-> TumorRestVas.AlbCold
	k_on_toAlb*TumorRestVas.Cold*TumorRestVas.Albumin/TumorRestVas-k_off_toAlb*TumorRestVas.AlbCold
143	TumorRestVas.Hot <-> TumorRestInt.Hot
	PS_TumorRest/TumorRestVas*TumorRestVas.Hot - PS_TumorRest/TumorRestInt*TumorRestInt.Hot
144	TumorRestVas.AlbHot <-> TumorRestInt.AlbHot
	PSAlb_TumorRest/TumorRestVas*TumorRestVas.AlbHot - PSAlb_TumorRest/TumorRestInt*TumorRestInt.AlbHot
145	TumorRestVas.AlbCold <-> TumorRestInt.AlbCold
	PSAlb_TumorRest/TumorRestVas*TumorRestVas.AlbCold - PSAlb_TumorRest/TumorRestInt*TumorRestInt.AlbCold
146	TumorRestVas.Cold <-> TumorRestInt.Cold
	PS_TumorRest/TumorRestVas * TumorRestVas.Cold - PS_TumorRest/TumorRestInt*TumorRestInt.Cold
147	TumorRestInt.Hot <-> TumorRestBound.Hot

	*k_on/TumorRestInt*TumorRestInt.Hot*TumorRest.R - k_off*TumorRestBound.Hot*
148	TumorRestInt.Cold <-> TumorRestBound.Cold
	*k_on/TumorRestInt*TumorRestInt.Cold*TumorRest.R - k_off*TumorRestBound.Cold*
149	TumorRestBound.Hot -> TumorRestIntern.Hot
	*lambdaIntern_TumorRest*TumorRestBound.Hot*
150	TumorRestBound.Cold -> TumorRestIntern.Cold
	*lambdaIntern_TumorRest*TumorRestBound.Cold*
151	Art.Hot -> TumorRestVas.Hot
	*F_TumorRest/Art*Art.Hot*
152	Art.AlbHot -> TumorRestVas.AlbHot
	*F_TumorRest/Art*Art.AlbHot*
153	Art.AlbCold <-> TumorRestVas.AlbCold
	*F_TumorRest/Art*Art.AlbCold*
154	Art.Cold <-> TumorRestVas.Cold
	*F_TumorRest/Art*Art.Cold*
155	TumorRestVas.Hot -> Vein.Hot
	*F_TumorRest/TumorRestVas*TumorRestVas.Hot*
156	TumorRestVas.AlbHot -> Vein.AlbHot
	*F_TumorRest/TumorRestVas*TumorRestVas.AlbHot*
157	TumorRestVas.AlbCold -> Vein.AlbCold
	*F_TumorRest/TumorRestVas*TumorRestVas.AlbCold*
158	TumorRestVas.Cold -> Vein.Cold
	*F_TumorRest/TumorRestVas*TumorRestVas.Cold*
159	TumorRestInt.Hot -> TumorRestInt.Cold
	*lambdaPhys*TumorRestInt.Hot*
160	TumorRestInt.Hot + TumorRestInt.Albumin -> TumorRestInt.AlbHot
	*k_on_toAlb*TumorRestInt.Hot*TumorRestInt.Albumin/TumorRestInt-k_off_toAlb*TumorRestInt.AlbHot*
161	TumorRestInt.AlbHot -> TumorRestInt.AlbCold
	*lambdaPhys*TumorRestInt.AlbHot*
162	TumorRestInt.Cold + TumorRestInt.Albumin <-> TumorRestInt.AlbCold
	*k_on_toAlb*TumorRestInt.Cold*TumorRestInt.Albumin/TumorRestInt-k_off_toAlb*TumorRestInt.AlbCold*
163	TumorRestBound.Hot -> TumorRestBound.Cold
	*lambdaPhys*TumorRestBound.Hot*
164	TumorRestIntern.Hot -> TumorRestIntern.Cold
	*lambdaPhys*TumorRestIntern.Hot*
165	TumorRestIntern.Hot -> null
	*lambdaRel_TumorRest*TumorRestIntern.Hot*
166	TumorRestIntern.Cold -> null
	*lambdaRel_TumorRest*TumorRestIntern.Cold*
167	SpleenVas.Hot -> SpleenVas.Cold
	*lambdaPhys*SpleenVas.Hot*
168	SpleenVas.Hot + SpleenVas.Albumin <-> SpleenVas.AlbHot
	*k_on_toAlb*SpleenVas.Hot*SpleenVas.Albumin/SpleenVas-k_off_toAlb*SpleenVas.AlbHot*
169	SpleenVas.AlbHot -> SpleenVas.AlbCold
	*lambdaPhys*SpleenVas.AlbHot*
170	SpleenVas.Cold + SpleenVas.Albumin <-> SpleenVas.AlbCold
	*k_on_toAlb*SpleenVas.Cold*SpleenVas.Albumin/SpleenVas-k_off_toAlb*SpleenVas.AlbCold*
171	SpleenVas.Hot <-> SpleenInt.Hot
	*PS_Spleen/SpleenVas*SpleenVas.Hot - PS_Spleen/SpleenInt*SpleenInt.Hot*
172	SpleenVas.Cold <-> SpleenInt.Cold
	*PS_Spleen/SpleenVas*SpleenVas.Cold - PS_Spleen/SpleenInt*SpleenInt.Cold*
173	SpleenInt.Hot <-> SpleenBound.Hot
	*k_on/SpleenInt*SpleenInt.Hot*Spleen.R - k_off*SpleenBound.Hot*
174	SpleenInt.Cold <-> SpleenBound.Cold
	*k_on/SpleenInt*SpleenInt.Cold*Spleen.R - k_off*SpleenBound.Cold*
175	SpleenBound.Hot -> SpleenIntern.Hot

	lambdaIntern_Spleen*SpleenBound.Hot
176	SpleenBound.Cold -> SpleenIntern.Cold
	lambdaIntern_Spleen*SpleenBound.Cold
177	Art.Hot -> SpleenVas.Hot
	F_Spleen/Art*Art.Hot
178	Art.AlbHot -> SpleenVas.AlbHot
	F_Spleen/Art*Art.AlbHot
179	Art.AlbCold -> SpleenVas.AlbCold
	F_Spleen/Art*Art.AlbCold
180	Art.Cold -> SpleenVas.Cold
	F_Spleen/Art*Art.Cold
181	SpleenInt.Hot -> SpleenInt.Cold
	lambdaPhys*SpleenInt.Hot
182	SpleenBound.Hot -> SpleenBound.Cold
	lambdaPhys*SpleenBound.Hot
183	SpleenIntern.Hot -> SpleenIntern.Cold
	lambdaPhys*SpleenIntern.Hot
184	SpleenIntern.Hot -> null
	lambdaRel_Spleen*SpleenIntern.Hot
185	SpleenIntern.Cold -> null
	lambdaRel_Spleen*SpleenIntern.Cold
186	LiverVas.Hot -> LiverVas.Cold
	lambdaPhys*LiverVas.Hot
187	LiverVas.Hot + LiverVas.Albumin <-> LiverVas.AlbHot
	k_on_toAlb*LiverVas.Hot*LiverVas.Albumin/LiverVas-k_off_toAlb*LiverVas.AlbHot
188	LiverVas.AlbHot -> LiverVas.AlbCold
	lambdaPhys*LiverVas.AlbHot
189	LiverVas.Cold + LiverVas.Albumin <-> LiverVas.AlbCold
	k_on_toAlb*LiverVas.Cold*LiverVas.Albumin/LiverVas-k_off_toAlb*LiverVas.AlbCold
190	LiverVas.Hot <-> LiverInt.Hot
	PS_Liver/LiverVas*LiverVas.Hot - PS_Liver/LiverInt*LiverInt.Hot
191	LiverVas.Cold <-> LiverInt.Cold
	PS_Liver/LiverVas*LiverVas.Cold - PS_Liver/LiverInt*LiverInt.Cold
192	LiverInt.Hot <-> LiverBound.Hot
	k_on/LiverInt*LiverInt.Hot*Liver.R - k_off*LiverBound.Hot
193	LiverInt.Cold <-> LiverBound.Cold
	k_on/LiverInt*LiverInt.Cold*Liver.R - k_off*LiverBound.Cold
194	LiverBound.Hot -> LiverIntern.Hot
	lambdaIntern_Liver*LiverBound.Hot
195	LiverBound.Cold -> LiverIntern.Cold
	lambdaIntern_Liver*LiverBound.Cold
196	Art.Hot -> LiverVas.Hot
	F_Liver/Art*Art.Hot
197	Art.AlbHot -> LiverVas.AlbHot
	F_Liver/Art*Art.AlbHot
198	Art.AlbCold -> LiverVas.AlbCold
	F_Liver/Art*Art.AlbCold
199	Art.Cold -> LiverVas.Cold
	F_Liver/Art*Art.Cold
200	LiverVas.Hot -> Vein.Hot
	F_Liver/LiverVas*LiverVas.Hot
201	LiverVas.AlbHot -> Vein.AlbHot
	F_Liver/LiverVas*LiverVas.AlbHot
202	LiverVas.AlbCold -> Vein.AlbCold
	F_Liver/LiverVas*LiverVas.AlbCold
203	LiverVas.Cold -> Vein.Cold
	F_Liver/LiverVas*LiverVas.Cold

204	LiverInt.Hot -> LiverInt.Cold
	*lambdaPhys*LiverInt.Hot*
205	LiverBound.Hot -> LiverBound.Cold
	*lambdaPhys*LiverBound.Hot*
206	LiverIntern.Hot -> LiverIntern.Cold
	*lambdaPhys*LiverIntern.Hot*
207	LiverIntern.Hot -> null
	*lambdaRel_Liver*LiverIntern.Hot*
208	LiverIntern.Cold -> null
	*lambdaRel_Liver*LiverIntern.Cold*
209	ProstateVas.Hot -> ProstateVas.Cold
	*lambdaPhys*ProstateVas.Hot*
210	ProstateVas.Hot + ProstateVas.Albumin <-> ProstateVas.AlbHot
	*k_on_toAlb*ProstateVas.Hot*ProstateVas.Albumin/ProstateVas-k_off_toAlb*ProstateVas.AlbHot*
211	ProstateVas.AlbHot -> ProstateVas.AlbCold
	*lambdaPhys*ProstateVas.AlbHot*
212	ProstateVas.Cold + ProstateVas.Albumin <-> ProstateVas.AlbCold
	*k_on_toAlb*ProstateVas.Cold*ProstateVas.Albumin/ProstateVas-k_off_toAlb*ProstateVas.AlbCold*
213	ProstateVas.Hot <-> ProstateInt.Hot
	*PS_Prostate/ProstateVas*ProstateVas.Hot - PS_Prostate/ProstateInt*ProstateInt.Hot*
214	ProstateVas.Cold <-> ProstateInt.Cold
	*PS_Prostate/ProstateVas*ProstateVas.Cold - PS_Prostate/ProstateInt*ProstateInt.Cold*
215	ProstateInt.Hot <-> ProstateBound.Hot
	*k_on/ProstateInt*ProstateInt.Hot*Prostate.R-k_off*ProstateBound.Hot*
216	ProstateInt.Cold <-> ProstateBound.Cold
	*k_on/ProstateInt*ProstateInt.Cold*Prostate.R-k_off*ProstateBound.Cold*
217	ProstateBound.Hot -> ProstateIntern.Hot
	*lambdaIntern_Prostate*ProstateBound.Hot*
218	ProstateBound.Cold -> ProstateIntern.Cold
	*lambdaIntern_Prostate*ProstateBound.Cold*
219	Art.Hot -> ProstateVas.Hot
	*F_Prostate/Art*Art.Hot*
220	Art.AlbHot -> ProstateVas.AlbHot
	*F_Prostate/Art*Art.AlbHot*
221	Art.AlbCold -> ProstateVas.AlbCold
	*F_Prostate/Art*Art.AlbCold*
222	Art.Cold -> ProstateVas.Cold
	*F_Prostate/Art*Art.Cold*
223	ProstateVas.Hot -> Vein.Hot
	*F_Prostate/ProstateVas*ProstateVas.Hot*
224	ProstateVas.AlbHot -> Vein.AlbHot
	*F_Prostate/ProstateVas*ProstateVas.AlbHot*
225	ProstateVas.AlbCold -> Vein.AlbCold
	*F_Prostate/ProstateVas*ProstateVas.AlbCold*
226	ProstateVas.Cold -> Vein.Cold
	*F_Prostate/ProstateVas*ProstateVas.Cold*
227	ProstateInt.Hot -> ProstateInt.Cold
	*lambdaPhys*ProstateInt.Hot*
228	ProstateBound.Hot -> ProstateBound.Cold
	*lambdaPhys*ProstateBound.Hot*
229	ProstateIntern.Hot -> ProstateIntern.Cold
	*lambdaPhys*ProstateIntern.Hot*
230	ProstateIntern.Hot -> null
	*lambdaRel_Prostate*ProstateIntern.Hot*
231	ProstateIntern.Cold -> null
	*lambdaRel_Prostate*ProstateIntern.Cold*

232	GIVas.Hot -> GIVas.Cold
	lambdaPhys*GIVas.Hot
233	GIVas.Hot + GIVas.Albumin <-> GIVas.AlbHot
	k_on_toAlb*GIVas.Hot*GIVas.Albumin/GIVas-k_off_toAlb*GIVas.AlbHot
234	GIVas.AlbHot -> GIVas.AlbCold
	lambdaPhys*GIVas.AlbHot
235	GIVas.Cold + GIVas.Albumin <-> GIVas.AlbCold
	k_on_toAlb*GIVas.Cold*GIVas.Albumin/GIVas-k_off_toAlb*GIVas.AlbCold
236	GIVas.Hot <-> GIInt.Hot
	PS_GI/GIVas*GIVas.Hot - PS_GI/GIInt*GIInt.Hot
237	GIVas.Cold <-> GIInt.Cold
	PS_GI/GIVas*GIVas.Cold - PS_GI/GIInt*GIInt.Cold
238	GIInt.Hot <-> GIBound.Hot
	k_on/GIInt*GIInt.Hot*GI.R - k_off*GIBound.Hot
239	GIInt.Cold <-> GIBound.Cold
	k_on/GIInt*GIInt.Cold*GI.R - k_off*GIBound.Cold
240	GIBound.Hot -> GIIntern.Hot
	lambdaIntern_GI*GIBound.Hot
241	GIBound.Cold -> GIIntern.Cold
	lambdaIntern_GI*GIBound.Cold
242	Art.Hot -> GIVas.Hot
	F_GI/Art*Art.Hot
243	Art.AlbHot -> GIVas.AlbHot
	F_GI/Art*Art.AlbHot
244	Art.AlbCold -> GIVas.AlbCold
	F_GI/Art*Art.AlbCold
245	Art.Cold -> GIVas.Cold
	F_GI/Art*Art.Cold
246	GIInt.Hot -> GIInt.Cold
	lambdaPhys*GIInt.Hot
247	GIBound.Hot -> GIBound.Cold
	lambdaPhys*GIBound.Hot
248	GIIntern.Hot -> GIIntern.Cold
	lambdaPhys*GIIntern.Hot
249	GIIntern.Hot -> null
	lambdaRel_GI*GIIntern.Hot
250	GIIntern.Cold -> null
	lambdaRel_GI*GIIntern.Cold
251	RestVas.Hot -> RestVas.Cold
	lambdaPhys*RestVas.Hot
252	RestVas.Hot + RestVas.Albumin <-> RestVas.AlbHot
	k_on_toAlb*RestVas.Hot*RestVas.Albumin/RestVas-k_off_toAlb*RestVas.AlbHot
253	RestVas.AlbHot -> RestVas.AlbCold
	lambdaPhys*RestVas.AlbHot
254	RestVas.Cold + RestVas.Albumin <-> RestVas.AlbCold
	k_on_toAlb*RestVas.Cold*RestVas.Albumin/RestVas-k_off_toAlb*RestVas.AlbCold
255	RestVas.Hot <-> RestInt.Hot
	PS_Rest/RestVas*RestVas.Hot - PS_Rest/RestInt*RestInt.Hot
256	RestVas.Cold <-> RestInt.Cold
	PS_Rest/RestVas*RestVas.Cold - PS_Rest/RestInt*RestInt.Cold
257	Art.Hot -> RestVas.Hot
	F_Rest/Art*Art.Hot
258	Art.AlbHot -> RestVas.AlbHot
	F_Rest/Art*Art.AlbHot
259	Art.AlbCold -> RestVas.AlbCold
	F_Rest/Art*Art.AlbCold
260	Art.Cold -> RestVas.Cold

	F_Rest/Art*Art.Cold
261	RestVas.Hot -> Vein.Hot
	F_Rest/RestVas*RestVas.Hot
262	RestVas.AlbHot -> Vein.AlbHot
	F_Rest/RestVas*RestVas.AlbHot
263	RestVas.AlbCold -> Vein.AlbCold
	F_Rest/RestVas*RestVas.AlbCold
264	RestVas.Cold -> Vein.Cold
	F_Rest/RestVas*RestVas.Cold
265	RestInt.Hot -> RestInt.Cold
	lambdaPhys*RestInt.Hot
266	SkinVas.Hot -> SkinVas.Cold
	lambdaPhys*SkinVas.Hot
267	SkinVas.Hot + SkinVas.Albumin <-> SkinVas.AlbHot
	k_on_toAlb*SkinVas.Hot*SkinVas.Albumin/SkinVas-k_off_toAlb*SkinVas.AlbHot
268	SkinVas.AlbHot -> SkinVas.AlbCold
	lambdaPhys*SkinVas.AlbHot
269	SkinVas.Cold + SkinVas.Albumin <-> SkinVas.AlbCold
	k_on_toAlb*SkinVas.Cold*SkinVas.Albumin/SkinVas-k_off_toAlb*SkinVas.AlbCold
270	SkinVas.Hot <-> SkinInt.Hot
	PS_Skin/SkinVas*SkinVas.Hot - PS_Skin/SkinVas*SkinInt.Hot
271	SkinVas.Cold <-> SkinInt.Cold
	PS_Skin/SkinVas*SkinVas.Cold - PS_Skin/SkinInt*SkinInt.Cold
272	Art.Hot -> SkinVas.Hot
	F_Skin/Art*Art.Hot
273	Art.AlbHot -> SkinVas.AlbHot
	F_Skin/Art*Art.AlbHot
274	Art.AlbCold -> SkinVas.AlbCold
	F_Skin/Art*Art.AlbCold
275	Art.Cold -> SkinVas.Cold
	F_Skin/Art*Art.Cold
276	SkinVas.Hot -> Vein.Hot
	F_Skin/SkinVas*SkinVas.Hot
277	SkinVas.AlbHot -> Vein.AlbHot
	F_Skin/SkinVas*SkinVas.AlbHot
278	SkinVas.AlbCold -> Vein.AlbCold
	F_Skin/SkinVas*SkinVas.AlbCold
279	SkinVas.Cold -> Vein.Cold
	F_Skin/SkinVas*SkinVas.Cold
280	SkinInt.Hot -> SkinInt.Cold
	lambdaPhys*SkinInt.Hot
281	MuscleVas.Hot -> MuscleVas.Cold
	lambdaPhys*MuscleVas.Hot
282	MuscleVas.Hot + MuscleVas.Albumin <-> MuscleVas.AlbHot
	k_on_toAlb*MuscleVas.Hot*MuscleVas.Albumin/MuscleVas-k_off_toAlb*MuscleVas.AlbHot
283	MuscleVas.AlbHot -> MuscleVas.AlbCold
	lambdaPhys*MuscleVas.AlbHot
284	MuscleVas.Cold + MuscleVas.Albumin <-> MuscleVas.AlbCold
	k_on_toAlb*MuscleVas.Cold*MuscleVas.Albumin/MuscleVas-k_off_toAlb*MuscleVas.AlbCold
285	MuscleVas.Hot <-> MuscleInt.Hot
	PS_Muscle/MuscleVas*MuscleVas.Hot - PS_Muscle/MuscleInt*MuscleInt.Hot
286	MuscleVas.Cold <-> MuscleInt.Cold
	PS_Muscle/MuscleVas*MuscleVas.Cold - PS_Muscle/MuscleInt*MuscleInt.Cold
287	Art.Hot -> MuscleVas.Hot
	F_Muscle/Art*Art.Hot
288	Art.AlbHot -> MuscleVas.AlbHot

	*F_Muscle/Art*Art.AlbHot*
289	Art.AlbCold -> MuscleVas.AlbCold
	*F_Muscle/Art*Art.AlbCold*
290	Art.Cold -> MuscleVas.Cold
	*F_Muscle/Art*Art.Cold*
291	MuscleVas.Hot -> Vein.Hot
	*F_Muscle/MuscleVas*MuscleVas.Hot*
292	MuscleVas.AlbHot -> Vein.AlbHot
	*F_Muscle/MuscleVas*MuscleVas.AlbHot*
293	MuscleVas.AlbCold -> Vein.AlbCold
	*F_Muscle/MuscleVas*MuscleVas.AlbCold*
294	MuscleVas.Cold -> Vein.Cold
	*F_Muscle/MuscleVas*MuscleVas.Cold*
295	MuscleInt.Hot -> MuscleInt.Cold
	*lambdaPhys*MuscleInt.Hot*
296	BrainVas.Hot -> BrainVas.Cold
	*lambdaPhys*BrainVas.Hot*
297	BrainVas.Hot + BrainVas.Albumin <-> BrainVas.AlbHot
	*k_on_toAlb*BrainVas.Hot*BrainVas.Albumin/BrainVas-k_off_toAlb*BrainVas.AlbHot*
298	BrainVas.AlbHot -> BrainVas.AlbCold
	*lambdaPhys*BrainVas.AlbHot*
299	BrainVas.Cold + BrainVas.Albumin <-> BrainVas.AlbCold
	*k_on_toAlb*BrainVas.Cold*BrainVas.Albumin/BrainVas-k_off_toAlb*BrainVas.AlbCold*
300	BrainVas.Hot <-> BrainInt.Hot
	*PS_Brain/BrainVas*BrainVas.Hot-PS_Brain/BrainInt*BrainInt.Hot*
301	BrainVas.Cold <-> BrainInt.Cold
	*PS_Brain/BrainVas*BrainVas.Cold - PS_Brain/BrainInt*BrainInt.Cold*
302	Art.Hot -> BrainVas.Hot
	*F_Brain/Art*Art.Hot*
303	Art.AlbHot -> BrainVas.AlbHot
	*F_Brain/Art*Art.AlbHot*
304	Art.AlbCold -> BrainVas.AlbCold
	*F_Brain/Art*Art.AlbCold*
305	Art.Cold -> BrainVas.Cold
	*F_Brain/Art*Art.Cold*
306	BrainVas.Hot -> Vein.Hot
	*F_Brain/BrainVas*BrainVas.Hot*
307	BrainVas.AlbHot -> Vein.AlbHot
	*F_Brain/BrainVas*BrainVas.AlbHot*
308	BrainVas.AlbCold -> Vein.AlbCold
	*F_Brain/BrainVas*BrainVas.AlbCold*
309	BrainVas.Cold -> Vein.Cold
	*F_Brain/BrainVas*BrainVas.Cold*
310	BrainInt.Hot -> BrainInt.Cold
	*lambdaPhys*BrainInt.Hot*
311	RedMarrowVas.Hot -> RedMarrowVas.Cold
	*lambdaPhys*RedMarrowVas.Hot*
312	RedMarrowVas.Hot + RedMarrowVas.Albumin <-> RedMarrowVas.AlbHot
	*k_on_toAlb*RedMarrowVas.Hot*RedMarrowVas.Albumin/RedMarrowVas-k_off_toAlb*RedMarrowVas.AlbHot*
313	RedMarrowVas.AlbHot -> RedMarrowVas.AlbCold
	*lambdaPhys*RedMarrowVas.AlbHot*
314	RedMarrowVas.Cold + RedMarrowVas.Albumin <-> RedMarrowVas.AlbCold
	*k_on_toAlb*RedMarrowVas.Cold*RedMarrowVas.Albumin/RedMarrowVas-k_off_toAlb*RedMarrowVas.AlbCold*
315	RedMarrowVas.Hot <-> RedMarrowInt.Hot
	*PS_RedMarrow/RedMarrowVas*RedMarrowVas.Hot - PS_RedMarrow/RedMarrowInt*RedMarrowInt.Hot*
316	RedMarrowVas.Cold <-> RedMarrowInt.Cold
	*PS_RedMarrow/RedMarrowVas*RedMarrowVas.Cold - PS_RedMarrow/RedMarrowInt*RedMarrowInt.Cold*

317	Art.Hot -> RedMarrowVas.Hot
	*F_RedMarrow/Art*Art.Hot*
318	Art.AlbHot -> RedMarrowVas.AlbHot
	*F_RedMarrow/Art*Art.AlbHot*
319	Art.AlbCold -> RedMarrowVas.AlbCold
	*F_RedMarrow/Art*Art.AlbCold*
320	Art.Cold -> RedMarrowVas.Cold
	*F_RedMarrow/Art*Art.Cold*
321	RedMarrowVas.Hot -> Vein.Hot
	*F_RedMarrow/RedMarrowVas*RedMarrowVas.Hot*
322	RedMarrowVas.AlbHot -> Vein.AlbHot
	*F_RedMarrow/RedMarrowVas*RedMarrowVas.AlbHot*
323	RedMarrowVas.AlbCold -> Vein.AlbCold
	*F_RedMarrow/RedMarrowVas*RedMarrowVas.AlbCold*
324	RedMarrowVas.Cold -> Vein.Cold
	*F_RedMarrow/RedMarrowVas*RedMarrowVas.Cold*
325	RedMarrowInt.Hot -> RedMarrowInt.Cold
	*lambdaPhys*RedMarrowInt.Hot*
326	LungsVas.Hot -> LungsVas.Cold
	*lambdaPhys*LungsVas.Hot*
327	LungsVas.Hot + LungsVas.Albumin <-> LungsVas.AlbHot
	*k_on_toAlb*LungsVas.Hot*LungsVas.Albumin/LungsVas-k_off_toAlb*LungsVas.AlbHot*
328	LungsVas.AlbHot -> LungsVas.AlbCold
	*lambdaPhys*LungsVas.AlbHot*
329	LungsVas.Cold + LungsVas.Albumin <-> LungsVas.AlbCold
	*k_on_toAlb*LungsVas.Cold*LungsVas.Albumin/LungsVas-k_off_toAlb*LungsVas.AlbCold*
330	LungsVas.Hot <-> LungsInt.Hot
	*PS_Lungs/LungsVas*LungsVas.Hot- PS_Lungs/LungsInt*LungsInt.Hot*
331	LungsVas.Cold <-> LungsInt.Cold
	*PS_Lungs/LungsVas*LungsVas.Cold - PS_Lungs/LungsInt*LungsInt.Cold*
332	LungsVas.Hot -> Art.Hot
	*F_Lungs/LungsVas*LungsVas.Hot*
333	LungsVas.AlbHot -> Art.AlbHot
	*F_Lungs/LungsVas*LungsVas.AlbHot*
334	LungsVas.AlbCold -> Art.AlbCold
	*F_Lungs/LungsVas*LungsVas.AlbCold*
335	LungsVas.Cold -> Art.Cold
	*F_Lungs/LungsVas*LungsVas.Cold*
336	Vein.Hot -> LungsVas.Hot
	*F_Lungs/Vein*Vein.Hot*
337	Vein.AlbHot -> LungsVas.AlbHot
	*F_Lungs/Vein*Vein.AlbHot*
338	Vein.AlbCold -> LungsVas.AlbCold
	*F_Lungs/Vein*Vein.AlbCold*
339	Vein.Cold -> LungsVas.Cold
	*F_Lungs/Vein*Vein.Cold*
340	LungsInt.Hot -> LungsInt.Cold
	*lambdaPhys*LungsInt.Hot*
341	AdiposeVas.Hot -> AdiposeVas.Cold
	*lambdaPhys*AdiposeVas.Hot*
342	AdiposeVas.Hot + AdiposeVas.Albumin <-> AdiposeVas.AlbHot
	*k_on_toAlb*AdiposeVas.Hot*AdiposeVas.Albumin/AdiposeVas-k_off_toAlb*AdiposeVas.AlbHot*
343	AdiposeVas.AlbHot -> AdiposeVas.AlbCold
	*lambdaPhys*AdiposeVas.AlbHot*
344	AdiposeVas.Cold + AdiposeVas.Albumin <-> AdiposeVas.AlbCold
	*k_on_toAlb*AdiposeVas.Cold*AdiposeVas.Albumin/AdiposeVas-k_off_toAlb*AdiposeVas.AlbCold*

345	AdiposeVas.Hot <-> AdiposeInt.Hot
	*PS_Adipose/AdiposeVas*AdiposeVas.Hot - PS_Adipose/AdiposeInt*AdiposeInt.Hot*
346	AdiposeVas.Cold <-> AdiposeInt.Cold
	*PS_Adipose/AdiposeVas*AdiposeVas.Cold - PS_Adipose/AdiposeInt*AdiposeInt.Cold*
347	Art.Hot -> AdiposeVas.Hot
	*F_Adipose/Art*Art.Hot*
348	Art.AlbHot -> AdiposeVas.AlbHot
	*F_Adipose/Art*Art.AlbHot*
349	Art.AlbCold -> AdiposeVas.AlbCold
	*F_Adipose/Art*Art.AlbCold*
350	Art.Cold -> AdiposeVas.Cold
	*F_Adipose/Art*Art.Cold*
351	AdiposeVas.Hot -> Vein.Hot
	*F_Adipose/AdiposeVas*AdiposeVas.Hot*
352	AdiposeVas.AlbHot -> Vein.AlbHot
	*F_Adipose/AdiposeVas*AdiposeVas.AlbHot*
353	AdiposeVas.AlbCold -> Vein.AlbCold
	*F_Adipose/AdiposeVas*AdiposeVas.AlbCold*
354	AdiposeVas.Cold -> Vein.Cold
	*F_Adipose/AdiposeVas*AdiposeVas.Cold*
355	AdiposeInt.Hot -> AdiposeInt.Cold
	*lambdaPhys*AdiposeInt.Hot*
356	KidneyVas.Hot -> KidneyInt.Hot
	*GFR*phi/KidneyVas*KidneyVas.Hot*
357	KidneyVas.Cold -> KidneyInt.Cold
	*GFR*phi/KidneyVas*KidneyVas.Cold*
358	KidneyInt.Hot -> KidneyIntera.Hot
	*GFR*phi*(1-f_exc)/KidneyInt*KidneyInt.Hot*
359	KidneyInt.Cold -> KidneyIntera.Cold
	*GFR*phi*(1-f_exc)/KidneyInt*KidneyInt.Cold*
360	KidneyIntera.Hot -> KidneyVas.Hot
	*GFR*phi*(1-f_exc)/KidneyIntern*KidneyIntera.Hot*
361	KidneyIntera.Cold -> KidneyVas.Cold
	*GFR*phi*(1-f_exc)/KidneyIntern*KidneyIntera.Cold*
362	KidneyInt.Cold -> null
	*GFR*phi*f_exc/KidneyInt*KidneyInt.Cold*
363	KidneyInt.Hot -> null
	*GFR*phi*f_exc/KidneyInt*KidneyInt.Hot*
364	SpleenVas.Hot -> LiverVas.Hot
	*F_Spleen/SpleenVas*SpleenVas.Hot*
365	GIVas.Hot -> LiverVas.Hot
	*F_GI/GIVas*GIVas.Hot*
366	SpleenVas.AlbHot -> LiverVas.AlbHot
	*F_Spleen/SpleenVas*SpleenVas.AlbHot*
367	SpleenVas.AlbCold -> LiverVas.AlbCold
	*F_Spleen/SpleenVas*SpleenVas.AlbCold*
368	SpleenVas.Cold -> LiverVas.Cold
	*F_Spleen/SpleenVas * SpleenVas.Cold*
369	GIVas.AlbHot -> LiverVas.AlbHot
	*F_GI/GIVas*GIVas.AlbHot*
370	GIVas.AlbCold -> LiverVas.AlbCold
	*F_GI/GIVas*GIVas.AlbCold*
371	GIVas.Cold -> LiverVas.Cold
	*F_GI/GIVas*GIVas.Cold*
372	Art.Albumin -> Tumor2Vas.Albumin
	*F_Tumor2/Art*Art.Albumin*

373	Art.Albumin -> Tumor1Vas.Albumin
	*F_Tumor1/Art*Art.Albumin*
374	Tumor1Vas.Albumin -> Vein.Albumin
	*F_Tumor1/Tumor1Vas*Tumor1Vas.Albumin*
375	Tumor2Vas.Albumin -> Vein.Albumin
	*F_Tumor2/Tumor2Vas*Tumor2Vas.Albumin*
376	Art.Albumin -> KidneyVas.Albumin
	*F_Kidney/Art*Art.Albumin*
377	KidneyVas.Albumin -> Vein.Albumin
	*F_Kidney/KidneyVas*KidneyVas.Albumin*
378	Art.Albumin -> SpleenVas.Albumin
	*F_Spleen/Art*Art.Albumin*
379	SpleenVas.Albumin -> Vein.Albumin
	*F_Spleen/SpleenVas*SpleenVas.Albumin*
380	Art.Albumin -> ProstateVas.Albumin
	*F_Prostate/Art*Art.Albumin*
381	ProstateVas.Albumin -> Vein.Albumin
	*F_Prostate/ProstateVas*ProstateVas.Albumin*
382	Art.Albumin -> GIVas.Albumin
	*F_GI/Art*Art.Albumin*
383	GIVas.Albumin -> Vein.Albumin
	*F_GI/GIVas*GIVas.Albumin*
384	Art.Albumin -> SkinVas.Albumin
	*F_Skin/Art*Art.Albumin*
385	SkinVas.Albumin -> Vein.Albumin
	*F_Skin/SkinVas*SkinVas.Albumin*
386	Art.Albumin -> BrainVas.Albumin
	*F_Brain/Art*Art.Albumin*
387	BrainVas.Albumin -> Vein.Albumin
	*F_Brain/BrainVas*BrainVas.Albumin*
388	Vein.Albumin -> LungsVas.Albumin
	*F_Lungs/Vein*Vein.Albumin*
389	LungsVas.Albumin -> Art.Albumin
	*F_Lungs/LungsVas*LungsVas.Albumin*
390	Art.Albumin -> HeartVas.Albumin
	*F_Heart/Art*Art.Albumin*
391	HeartVas.Albumin -> Vein.Albumin
	*F_Heart/HeartVas*HeartVas.Albumin*
392	Art.Albumin -> BoneVas.Albumin
	*F_Bone/Art*Art.Albumin*
393	BoneVas.Albumin -> Vein.Albumin
	*F_Bone/BoneVas*BoneVas.Albumin*
394	Art.Albumin -> AdiposeVas.Albumin
	*F_Adipose/Art*Art.Albumin*
395	AdiposeVas.Albumin -> Vein.Albumin
	*F_Adipose/AdiposeVas*AdiposeVas.Albumin*
396	Art.Albumin -> RedMarrowVas.Albumin
	*F_RedMarrow/Art*Art.Albumin*
397	RedMarrowVas.Albumin -> Vein.Albumin
	*F_RedMarrow/RedMarrowVas*RedMarrowVas.Albumin*
398	Art.Albumin -> MuscleVas.Albumin
	*F_Muscle/Art*Art.Albumin*
399	MuscleVas.Albumin -> Vein.Albumin
	*F_Muscle/MuscleVas*MuscleVas.Albumin*
400	Art.Albumin -> RestVas.Albumin
	*F_Rest/Art*Art.Albumin*

401	RestVas.Albumin -> Vein.Albumin
	F_Rest/RestVas*RestVas.Albumin
402	Art.Albumin -> LiverVas.Albumin
	F_Liver/Art*Art.Albumin
403	LiverVas.Albumin -> Vein.Albumin
	F_Liver/LiverVas*LiverVas.Albumin
404	Art.Albumin -> SGVas.Albumin
	F_SG/Art*Art.Albumin
405	SGVas.Albumin -> Vein.Albumin
	F_SG/SGVas*SGVas.Albumin
406	Art.Albumin -> TumorRestVas.Albumin
	F_TumorRest/Art*Art.Albumin
407	TumorRestVas.Albumin -> Vein.Albumin
	F_TumorRest/TumorRestVas*TumorRestVas.Albumin
408	Tumor2Vas.Albumin <-> Tumor2Int.Albumin
	PSAlb_Tumor2/Tumor2Vas*Tumor2Vas.Albumin - PSAlb_Tumor2/Tumor2Int*Tumor2Int.Albumin
409	Tumor1Vas.Albumin <-> Tumor1Int.Albumin
	PSAlb_Tumor1/Tumor1Vas*Tumor1Vas.Albumin - PSAlb_Tumor1/Tumor1Int*Tumor1Int.Albumin
410	TumorRestVas.Albumin <-> TumorRestInt.Albumin
	PSAlb_TumorRest/TumorRestVas*TumorRestVas.Albumin - PSAlb_TumorRest/TumorRestInt*TumorRestInt.Albumin

Observables

	Observables
1	Tumor1_TotalHot = Tumor1Vas.Hot + Tumor1Int.Hot + Tumor1Bound.Hot + Tumor1Intern.Hot + Tumor1Vas.AlbHot + Tumor1Int.AlbHot
2	Tumor2_TotalHot = Tumor2Vas.Hot + Tumor2Int.Hot + Tumor2Bound.Hot + Tumor2Intern.Hot + Tumor2Vas.AlbHot + Tumor2Int.AlbHot
3	TumorTotalHot = Tumor1_TotalHot
4	KidneyTotalHot = KidneyVas.Hot + KidneyInt.Hot + KidneyBound.Hot + KidneyIntern.Hot
5	SGTotalHot = SGVas.Hot + SGInt.Hot + SGBound.Hot + SGIntern.Hot
6	LiverTotalHot = LiverVas.Hot + LiverInt.Hot + LiverBound.Hot + LiverIntern.Hot
7	ProstateTotalHot = ProstateVas.Hot + ProstateInt.Hot + ProstateBound.Hot + ProstateIntern.Hot
8	SpleenTotalHot = SpleenVas.Hot + SpleenInt.Hot + SpleenBound.Hot + SpleenIntern.Hot
9	RedMarrowTotalHot = RedMarrowVas.Hot + RedMarrowInt.Hot
10	BoneTotalHot = BoneVas.Hot + BoneInt.Hot
11	TIA_TumorTotal = trapz(time, TumorTotalHot .* lambdaPhys ./ 60 .* numberPerNanomole)
12	TIA_Kidney = trapz(time, KidneyTotalHot .* lambdaPhys ./ 60 .* numberPerNanomole)
13	TIA_SG = trapz(time, SGTotalHot .* lambdaPhys ./ 60 .* numberPerNanomole)
14	TIA_Liver = trapz(time, LiverTotalHot .* lambdaPhys ./ 60 .* numberPerNanomole)
15	TIA_Spleen = trapz(time, SpleenTotalHot .* lambdaPhys ./ 60 .* numberPerNanomole)
16	TIA_RedMarrow = trapz(time, RedMarrowTotalHot .* lambdaPhys ./ 60 .* numberPerNanomole)
17	HotStuffBlood = Vein.Hot + Vein.AlbHot
18	BloodTIA = trapz(time, HotStuffBlood .* lambdaPhys ./ 60 .* numberPerNanomole)

Repeat Doses

	TargetName	Amount	Rate	StartTime	RepeatCount	Interval	Name
1	Vein.Hot	HotPerInjection	PerInjectionRate	0 (minute)	RepeatCount	RepeatTimeInterval	HotInjection
2	Vein.Cold	ColdPerInjection	PerInjectionRate	0 (minute)	RepeatCount	RepeatTimeInterval	ColdInjection

Model Equations

ODEs

	ODEs
1	d(Tumor2Vas.Hot)/dt = -(lambdaPhys*Tumor2Vas.Hot) - (k_on_toAlb*Tumor2Vas.Hot*Tumor2Vas.Albumin/Tumor2Vas-k_off_toAlb*Tumor2Vas.AlbHot) - (PS_Tumor2/Tumor2Vas*Tumor2Vas.Hot-PS_Tumor2/Tumor2Int*Tumor2Int.Hot) + (F_Tumor2/Art*Art.Hot) - (F_Tumor2/Tumor2Vas*Tumor2Vas.Hot)
2	d(Tumor2Vas.Cold)/dt = (lambdaPhys*Tumor2Vas.Hot) - (k_on_toAlb*Tumor2Vas.Cold*Tumor2Vas.Albumin/Tumor2Vas-k_off_toAlb*Tumor2Vas.AlbCold) - (PS_Tumor2/Tumor2Vas*Tumor2Vas.Cold-PS_Tumor2/Tumor2Int*Tumor2Int.Cold) + (F_Tumor2/Art*Art.Cold) - (F_Tumor2/Tumor2Vas*Tumor2Vas.Cold)

3	d(Tumor2Vas.AlbHot)/dt = (k_on_toAlb*Tumor2Vas.Hot*Tumor2Vas.Albumin/Tumor2Vas-k_off_toAlb*Tumor2Vas.AlbHot) - (lambdaPhys*Tumor2Vas.AlbHot) - (PSAlb_Tumor2/Tumor2Vas*Tumor2Vas.AlbHot) + (F_Tumor2/Art*Art.AlbHot) - (F_Tumor2/Tumor2Vas*Tumor2Vas.AlbHot)
4	d(Tumor2Vas.AlbCold)/dt = (lambdaPhys*Tumor2Vas.AlbHot) + (k_on_toAlb*Tumor2Vas.Cold*Tumor2Vas.Albumin/Tumor2Vas-k_off_toAlb*Tumor2Vas.AlbCold) - (PSAlb_Tumor2/Tumor2Vas*Tumor2Vas.AlbCold) + (F_Tumor2/Art*Art.AlbCold) - (F_Tumor2/Tumor2Vas*Tumor2Vas.AlbCold)
5	d(Tumor2Vas.Albumin)/dt = -(k_on_toAlb*Tumor2Vas.Hot*Tumor2Vas.Albumin/Tumor2Vas-k_off_toAlb*Tumor2Vas.AlbHot) - (k_on_toAlb*Tumor2Vas.Cold*Tumor2Vas.Albumin/Tumor2Vas-k_off_toAlb*Tumor2Vas.AlbCold) + (F_Tumor2/Art*Art.Albumin) - (F_Tumor2/Tumor2Vas*Tumor2Vas.Albumin) - (PSAlb_Tumor2/Tumor2Vas*Tumor2Vas.Albumin-PSAlb_Tumor2/Tumor2Int*Tumor2Int.Albumin)
6	d(Tumor2Int.Hot)/dt = (PS_Tumor2/Tumor2Vas*Tumor2Vas.Hot-PS_Tumor2/Tumor2Int*Tumor2Int.Hot) - (k_on/Tumor2Int*Tumor2Int.Hot*Tumor2.R-k_off*Tumor2Bound.Hot) - (lambdaPhys*Tumor2Int.Hot) - (k_on_toAlb*Tumor2Int.Hot*Tumor2Int.Albumin/Tumor2Int-k_off_toAlb*Tumor2Int.AlbHot)
7	d(Tumor2Int.Cold)/dt = (PS_Tumor2/Tumor2Vas*Tumor2Vas.Cold-PS_Tumor2/Tumor2Int*Tumor2Int.Cold) - (k_on/Tumor2Int*Tumor2Int.Cold*Tumor2.R-k_off*Tumor2Bound.Cold) + (lambdaPhys*Tumor2Int.Hot) - (k_on_toAlb*Tumor2Int.Cold*Tumor2Int.Albumin/Tumor2Int-k_off_toAlb*Tumor2Int.AlbCold)
8	d(Tumor2Int.AlbHot)/dt = (PSAlb_Tumor2/Tumor2Vas*Tumor2Vas.AlbHot) + (k_on_toAlb*Tumor2Int.Hot*Tumor2Int.Albumin/Tumor2Int-k_off_toAlb*Tumor2Int.AlbHot) - (lambdaPhys*Tumor2Int.AlbHot)
9	d(Tumor2Int.AlbCold)/dt = (PSAlb_Tumor2/Tumor2Vas*Tumor2Vas.AlbCold) + (lambdaPhys*Tumor2Int.AlbHot) + (k_on_toAlb*Tumor2Int.Cold*Tumor2Int.Albumin/Tumor2Int-k_off_toAlb*Tumor2Int.AlbCold)
10	d(Tumor2Int.Albumin)/dt = -(k_on_toAlb*Tumor2Int.Hot*Tumor2Int.Albumin/Tumor2Int-k_off_toAlb*Tumor2Int.AlbHot) - (k_on_toAlb*Tumor2Int.Cold*Tumor2Int.Albumin/Tumor2Int-k_off_toAlb*Tumor2Int.AlbCold) + (PSAlb_Tumor2/Tumor2Vas*Tumor2Vas.Albumin-PSAlb_Tumor2/Tumor2Int*Tumor2Int.Albumin)
11	d(Tumor2Bound.Hot)/dt = (k_on/Tumor2Int*Tumor2Int.Hot*Tumor2.R-k_off*Tumor2Bound.Hot) - (lambdaIntern_Tumor2*Tumor2Bound.Hot) - (lambdaPhys*Tumor2Bound.Hot)
12	d(Tumor2Bound.Cold)/dt = (k_on/Tumor2Int*Tumor2Int.Cold*Tumor2.R-k_off*Tumor2Bound.Cold) - (lambdaIntern_Tumor2*Tumor2Bound.Cold) + (lambdaPhys*Tumor2Bound.Hot)
13	d(Tumor2Intern.Hot)/dt = (lambdaIntern_Tumor2*Tumor2Bound.Hot) - (lambdaPhys*Tumor2Intern.Hot) - (lambdaRel_Tumor2*Tumor2Intern.Hot)
14	d(Tumor2Intern.Cold)/dt = (lambdaIntern_Tumor2*Tumor2Bound.Cold) + (lambdaPhys*Tumor2Intern.Hot) - (lambdaRel_Tumor2*Tumor2Intern.Cold)
15	d(Art.Hot)/dt = -(F_Tumor2/Art*Art.Hot) - (lambdaPhys*Art.Hot) - (k_on_toAlb*Art.Hot*Art.Albumin/Art-k_off_toAlb*Art.AlbHot) - (F_Tumor1/Art*Art.Hot) - (F_Kidney/Art*Art.Hot) - (F_Heart/Art*Art.Hot) - (F_SG/Art*Art.Hot) - (F_Bone/Art*Art.Hot) - (F_TumorRest/Art*Art.Hot) - (F_Spleen/Art*Art.Hot) - (F_Liver/Art*Art.Hot) - (F_Prostate/Art*Art.Hot) - (F_GI/Art*Art.Hot) - (F_Rest/Art*Art.Hot) - (F_Skin/Art*Art.Hot) - (F_Muscle/Art*Art.Hot) - (F_Brain/Art*Art.Hot) - (F_RedMarrow/Art*Art.Hot) + (F_Lungs/LungsVas*LungsVas.Hot) - (F_Adipose/Art*Art.Hot)
16	d(Art.Cold)/dt = -(F_Tumor2/Art*Art.Cold) + (lambdaPhys*Art.Hot) - (k_on_toAlb*Art.Cold*Art.Albumin/Art-k_off_toAlb*Art.AlbCold) - (F_Tumor1/Art*Art.Cold) - (F_Kidney/Art*Art.Cold) - (F_Heart/Art*Art.Cold) - (F_SG/Art*Art.Cold) - (F_Bone/Art*Art.Cold) - (F_TumorRest/Art*Art.Cold) - (F_Spleen/Art*Art.Cold) - (F_Liver/Art*Art.Cold) - (F_Prostate/Art*Art.Cold) - (F_GI/Art*Art.Cold) - (F_Rest/Art*Art.Cold) - (F_Skin/Art*Art.Cold) - (F_Muscle/Art*Art.Cold) - (F_Brain/Art*Art.Cold) - (F_RedMarrow/Art*Art.Cold) + (F_Lungs/LungsVas*LungsVas.Cold) - (F_Adipose/Art*Art.Cold)
17	d(Art.AlbHot)/dt = -(F_Tumor2/Art*Art.AlbHot) + (k_on_toAlb*Art.Hot*Art.Albumin/Art-k_off_toAlb*Art.AlbHot) - (lambdaPhys*Art.AlbHot) - (F_Tumor1/Art*Art.AlbHot) - (F_Kidney/Art*Art.AlbHot) - (F_Heart/Art*Art.AlbHot) - (F_SG/Art*Art.AlbHot) - (F_Bone/Art*Art.AlbHot) - (F_TumorRest/Art*Art.AlbHot) - (F_Spleen/Art*Art.AlbHot) - (F_Liver/Art*Art.AlbHot) - (F_Prostate/Art*Art.AlbHot) - (F_GI/Art*Art.AlbHot) - (F_Rest/Art*Art.AlbHot) - (F_Skin/Art*Art.AlbHot) - (F_Muscle/Art*Art.AlbHot) - (F_Brain/Art*Art.AlbHot) - (F_RedMarrow/Art*Art.AlbHot) + (F_Lungs/LungsVas*LungsVas.AlbHot) - (F_Adipose/Art*Art.AlbHot)
18	d(Art.AlbCold)/dt = -(F_Tumor2/Art*Art.AlbCold) + (lambdaPhys*Art.AlbHot) + (k_on_toAlb*Art.Cold*Art.Albumin/Art-k_off_toAlb*Art.AlbCold) - (F_Tumor1/Art*Art.AlbCold) - (F_Kidney/Art*Art.AlbCold) - (F_Heart/Art*Art.AlbCold) - (F_SG/Art*Art.AlbCold) - (F_Bone/Art*Art.AlbCold) - (F_TumorRest/Art*Art.AlbCold) - (F_Spleen/Art*Art.AlbCold) - (F_Liver/Art*Art.AlbCold) - (F_Prostate/Art*Art.AlbCold) - (F_GI/Art*Art.AlbCold) - (F_Rest/Art*Art.AlbCold) - (F_Skin/Art*Art.AlbCold) - (F_Muscle/Art*Art.AlbCold) - (F_Brain/Art*Art.AlbCold) - (F_RedMarrow/Art*Art.AlbCold) + (F_Lungs/LungsVas*LungsVas.AlbCold) - (F_Adipose/Art*Art.AlbCold)
19	d(Art.Albumin)/dt = -(k_on_toAlb*Art.Hot*Art.Albumin/Art-k_off_toAlb*Art.AlbHot) - (k_on_toAlb*Art.Cold*Art.Albumin/Art-k_off_toAlb*Art.AlbCold) - (F_Tumor2/Art*Art.Albumin) - (F_Tumor1/Art*Art.Albumin) - (F_Kidney/Art*Art.Albumin) - (F_Spleen/Art*Art.Albumin) - (F_Prostate/Art*Art.Albumin) - (F_GI/Art*Art.Albumin) - (F_Skin/Art*Art.Albumin) - (F_Brain/Art*Art.Albumin) + (F_Lungs/LungsVas*LungsVas.Albumin) - (F_Heart/Art*Art.Albumin) - (F_Bone/Art*Art.Albumin) - (F_Adipose/Art*Art.Albumin) - (F_RedMarrow/Art*Art.Albumin) - (F_Muscle/Art*Art.Albumin) - (F_Rest/Art*Art.Albumin) - (F_Liver/Art*Art.Albumin) - (F_SG/Art*Art.Albumin) - (F_TumorRest/Art*Art.Albumin)
20	d(Vein.Hot)/dt = (F_Tumor2/Tumor2Vas*Tumor2Vas.Hot) - (lambdaPhys*Vein.Hot) - (k_on_toAlb*Vein.Hot*Vein.Albumin/Vein-k_off_toAlb*Vein.AlbHot) + (F_Tumor1/Tumor1Vas*Tumor1Vas.Hot) + (F_Kidney/KidneyVas*KidneyVas.Hot) + (F_Heart/HeartVas*HeartVas.Hot) + (F_SG/SGVas*SGVas.Hot) + (F_Bone/BoneVas*BoneVas.Hot) + (F_TumorRest/TumorRestVas*TumorRestVas.Hot) + (F_Liver/LiverVas*LiverVas.Hot) + (F_Prostate/ProstateVas*ProstateVas.Hot) + (F_Rest/RestVas*RestVas.Hot) + (F_Skin/SkinVas*SkinVas.Hot) + (F_Muscle/MuscleVas*MuscleVas.Hot) + (F_Brain/BrainVas*BrainVas.Hot) + (F_RedMarrow/RedMarrowVas*RedMarrowVas.Hot) - (F_Lungs/Vein*Vein.Hot) + (F_Adipose/AdiposeVas*AdiposeVas.Hot) + HotInjection
21	d(Vein.Cold)/dt = (F_Tumor2/Tumor2Vas*Tumor2Vas.Cold) + (lambdaPhys*Vein.Hot) - (k_on_toAlb*Vein.Cold*Vein.Albumin/Vein-k_off_toAlb*Vein.AlbCold) + (F_Tumor1/Tumor1Vas*Tumor1Vas.Cold) + (F_Kidney/KidneyVas*KidneyVas.Cold) + (F_Heart/HeartVas*HeartVas.Cold) + (F_SG/SGVas*SGVas.Cold) + (F_Bone/BoneVas*BoneVas.Cold) + (F_TumorRest/TumorRestVas*TumorRestVas.Cold) + (F_Liver/LiverVas*LiverVas.Cold) + (F_Prostate/ProstateVas*ProstateVas.Cold) + (F_Rest/RestVas*RestVas.Cold) + (F_Skin/SkinVas*SkinVas.Cold) + (F_Muscle/MuscleVas*MuscleVas.Cold) + (F_Brain/BrainVas*BrainVas.Cold) + (F_RedMarrow/RedMarrowVas*RedMarrowVas.Cold) - (F_Lungs/Vein*Vein.Cold) + (F_Adipose/AdiposeVas*AdiposeVas.Cold) + ColdInjection
22	d(Vein.AlbHot)/dt = (F_Tumor2/Tumor2Vas*Tumor2Vas.AlbHot) + (k_on_toAlb*Vein.Hot*Vein.Albumin/Vein-k_off_toAlb*Vein.AlbHot) - (lambdaPhys*Vein.AlbHot) + (F_Tumor1/Tumor1Vas*Tumor1Vas.AlbHot) + (F_Kidney/KidneyVas*KidneyVas.AlbHot) + (F_Heart/HeartVas*HeartVas.AlbHot) + (F_SG/SGVas*SGVas.AlbHot) + (F_Bone/BoneVas*BoneVas.AlbHot) + (F_TumorRest/TumorRestVas*TumorRestVas.AlbHot) + (F_Liver/LiverVas*LiverVas.AlbHot) + (F_Prostate/ProstateVas*ProstateVas.AlbHot) + (F_Rest/RestVas*RestVas.AlbHot) + (F_Skin/SkinVas*SkinVas.AlbHot) + (F_Muscle/MuscleVas*MuscleVas.AlbHot) + (F_Brain/BrainVas*BrainVas.AlbHot) + (F_RedMarrow/RedMarrowVas*RedMarrowVas.AlbHot) - (F_Lungs/Vein*Vein.AlbHot) + (F_Adipose/AdiposeVas*AdiposeVas.AlbHot)

23	d(Vein.AlbCold)/dt = (F_Tumor2/Tumor2Vas*Tumor2Vas.AlbCold) + (lambdaPhys*Vein.AlbHot) + (k_on_toAlb*Vein.Cold*Vein.Albumin/Vein-k_off_toAlb*Vein.AlbCold) + (F_Tumor1/Tumor1Vas*Tumor1Vas.AlbCold) + (F_Kidney/KidneyVas*KidneyVas.AlbCold) + (F_Heart/HeartVas*HeartVas.AlbCold) + (F_SG/SGVas*SGVas.AlbCold) + (F_Bone/BoneVas*BoneVas.AlbCold) + (F_TumorRest/TumorRestVas*TumorRestVas.AlbCold) + (F_Liver/LiverVas*LiverVas.AlbCold) + (F_Prostate/ProstateVas*ProstateVas.AlbCold) + (F_Rest/RestVas*RestVas.AlbCold) + (F_Skin/SkinVas*SkinVas.AlbCold) + (F_Muscle/MuscleVas*MuscleVas.AlbCold) + (F_Brain/BrainVas*BrainVas.AlbCold) + (F_RedMarrow/RedMarrowVas*RedMarrowVas.AlbCold) - (F_Lungs/Vein*Vein.AlbCold) + (F_Adipose/AdiposeVas*AdiposeVas.AlbCold)
24	d(Vein.Albumin)/dt = -(k_on_toAlb*Vein.Hot*Vein.Albumin/Vein-k_off_toAlb*Vein.AlbHot) - (k_on_toAlb*Vein.Cold*Vein.Albumin/Vein-k_off_toAlb*Vein.AlbCold) + (F_Tumor1/Tumor1Vas*Tumor1Vas.Albumin) + (F_Tumor2/Tumor2Vas*Tumor2Vas.Albumin) + (F_Kidney/KidneyVas*KidneyVas.Albumin) + (F_Spleen/SpleenVas*SpleenVas.Albumin) + (F_Prostate/ProstateVas*ProstateVas.Albumin) + (F_GI/GIVas*GIVas.Albumin) + (F_Skin/SkinVas*SkinVas.Albumin) + (F_Brain/BrainVas*BrainVas.Albumin) - (F_Lungs/Vein*Vein.Albumin) + (F_Heart/HeartVas*HeartVas.Albumin) + (F_Bone/BoneVas*BoneVas.Albumin) + (F_Adipose/AdiposeVas*AdiposeVas.Albumin) + (F_RedMarrow/RedMarrowVas*RedMarrowVas.Albumin) + (F_Muscle/MuscleVas*MuscleVas.Albumin) + (F_Rest/RestVas*RestVas.Albumin) + (F_Liver/LiverVas*LiverVas.Albumin) + (F_SG/SGVas*SGVas.Albumin) + (F_TumorRest/TumorRestVas*TumorRestVas.Albumin)
25	d(Tumor1Vas.Hot)/dt = -(lambdaPhys*Tumor1Vas.Hot) - (k_on_toAlb*Tumor1Vas.Hot*Tumor1Vas.Albumin/Tumor1Vas-k_off_toAlb*Tumor1Vas.AlbHot) - (PS_Tumor1/Tumor1Vas*Tumor1Vas.Hot-PS_Tumor1/Tumor1Int*Tumor1Int.Hot) + (F_Tumor1/Art*Art.Hot) - (F_Tumor1/Tumor1Vas*Tumor1Vas.Hot)
26	d(Tumor1Vas.Cold)/dt = (lambdaPhys*Tumor1Vas.Hot) - (k_on_toAlb*Tumor1Vas.Cold*Tumor1Vas.Albumin/Tumor1Vas-k_off_toAlb*Tumor1Vas.AlbCold) - (PS_Tumor1/Tumor1Vas*Tumor1Vas.Cold-PS_Tumor1/Tumor1Int*Tumor1Int.Cold) + (F_Tumor1/Art*Art.Cold) - (F_Tumor1/Tumor1Vas*Tumor1Vas.Cold)
27	d(Tumor1Vas.AlbHot)/dt = (k_on_toAlb*Tumor1Vas.Hot*Tumor1Vas.Albumin/Tumor1Vas-k_off_toAlb*Tumor1Vas.AlbHot) - (lambdaPhys*Tumor1Vas.AlbHot) - (PSAlb_Tumor1/Tumor1Vas*Tumor1Vas.AlbHot-PSAlb_Tumor1/Tumor1Int*Tumor1Int.AlbHot) + (F_Tumor1/Art*Art.AlbHot) - (F_Tumor1/Tumor1Vas*Tumor1Vas.AlbHot)
28	d(Tumor1Vas.AlbCold)/dt = (lambdaPhys*Tumor1Vas.AlbHot) + (k_on_toAlb*Tumor1Vas.Cold*Tumor1Vas.Albumin/Tumor1Vas-k_off_toAlb*Tumor1Vas.AlbCold) - (PSAlb_Tumor1/Tumor1Vas*Tumor1Vas.AlbCold-PSAlb_Tumor1/Tumor1Int*Tumor1Int.AlbCold) + (F_Tumor1/Art*Art.AlbCold) - (F_Tumor1/Tumor1Vas*Tumor1Vas.AlbCold)
29	d(Tumor1Vas.Albumin)/dt = -(k_on_toAlb*Tumor1Vas.Hot*Tumor1Vas.Albumin/Tumor1Vas-k_off_toAlb*Tumor1Vas.AlbHot) - (k_on_toAlb*Tumor1Vas.Cold*Tumor1Vas.Albumin/Tumor1Vas-k_off_toAlb*Tumor1Vas.AlbCold) + (F_Tumor1/Art*Art.Albumin) - (F_Tumor1/Tumor1Vas*Tumor1Vas.Albumin) - (PSAlb_Tumor1/Tumor1Vas*Tumor1Vas.Albumin-PSAlb_Tumor1/Tumor1Int*Tumor1Int.Albumin)
30	d(Tumor1Int.Hot)/dt = (PS_Tumor1/Tumor1Vas*Tumor1Vas.Hot-PS_Tumor1/Tumor1Int*Tumor1Int.Hot) - (k_on/Tumor1Int*Tumor1Int.Hot*Tumor1.R-k_off*Tumor1Bound.Hot) - (lambdaPhys*Tumor1Int.Hot) - (k_on_toAlb*Tumor1Int.Hot*Tumor1Int.Albumin/Tumor1Int-k_off_toAlb*Tumor1Int.AlbHot)
31	d(Tumor1Int.Cold)/dt = (PS_Tumor1/Tumor1Vas*Tumor1Vas.Cold-PS_Tumor1/Tumor1Int*Tumor1Int.Cold) - (k_on/Tumor1Int*Tumor1.R*Tumor1Int.Cold-k_off*Tumor1Bound.Cold) + (lambdaPhys*Tumor1Int.Hot) - (k_on_toAlb*Tumor1Int.Cold*Tumor1Int.Albumin/Tumor1Int-k_off_toAlb*Tumor1Int.AlbCold)
32	d(Tumor1Int.AlbHot)/dt = (PSAlb_Tumor1/Tumor1Vas*Tumor1Vas.AlbHot-PSAlb_Tumor1/Tumor1Int*Tumor1Int.AlbHot) + (k_on_toAlb*Tumor1Int.Hot*Tumor1Int.Albumin/Tumor1Int-k_off_toAlb*Tumor1Int.AlbHot) - (lambdaPhys*Tumor1Int.AlbHot)
33	d(Tumor1Int.AlbCold)/dt = (PSAlb_Tumor1/Tumor1Vas*Tumor1Vas.AlbCold-PSAlb_Tumor1/Tumor1Int*Tumor1Int.AlbCold) + (lambdaPhys*Tumor1Int.AlbHot) + (k_on_toAlb*Tumor1Int.Cold*Tumor1Int.Albumin/Tumor1Int-k_off_toAlb*Tumor1Int.AlbCold)
34	d(Tumor1Int.Albumin)/dt = -(k_on_toAlb*Tumor1Int.Hot*Tumor1Int.Albumin/Tumor1Int-k_off_toAlb*Tumor1Int.AlbHot) - (k_on_toAlb*Tumor1Int.Cold*Tumor1Int.Albumin/Tumor1Int-k_off_toAlb*Tumor1Int.AlbCold) + (PSAlb_Tumor1/Tumor1Vas*Tumor1Vas.Albumin-PSAlb_Tumor1/Tumor1Int*Tumor1Int.Albumin)
35	d(Tumor1Bound.Hot)/dt = (k_on/Tumor1Int*Tumor1Int.Hot*Tumor1.R-k_off*Tumor1Bound.Hot) - (lambdaIntern_Tumor1*Tumor1Bound.Hot) - (lambdaPhys*Tumor1Bound.Hot)
36	d(Tumor1Bound.Cold)/dt = (k_on/Tumor1Int*Tumor1.R*Tumor1Int.Cold-k_off*Tumor1Bound.Cold) - (lambdaIntern_Tumor1*Tumor1Bound.Cold) + (lambdaPhys*Tumor1Bound.Hot)
37	d(Tumor1Intern.Hot)/dt = (lambdaIntern_Tumor1*Tumor1Bound.Hot) - (lambdaPhys*Tumor1Intern.Hot) - (lambdaRel_Tumor1*Tumor1Intern.Hot)
38	d(Tumor1Intern.Cold)/dt = (lambdaIntern_Tumor1*Tumor1Bound.Cold) + (lambdaPhys*Tumor1Intern.Hot) - (lambdaRel_Tumor1*Tumor1Intern.Cold)
39	d(KidneyVas.Hot)/dt = -(lambdaPhys*KidneyVas.Hot) - (k_on_toAlb*KidneyVas.Hot*KidneyVas.Albumin/KidneyVas-k_off_toAlb*KidneyVas.AlbHot) + (F_Kidney/Art*Art.Hot) - (F_Kidney/KidneyVas*KidneyVas.Hot) - (GFR*phi/KidneyVas*KidneyVas.Hot) + (GFR*phi*(1-f_exc)/KidneyIntern*KidneyIntera.Hot)
40	d(KidneyVas.Cold)/dt = (lambdaPhys*KidneyVas.Hot) - (k_on_toAlb*KidneyVas.Cold*KidneyVas.Albumin/KidneyVas-k_off_toAlb*KidneyVas.AlbCold) + (F_Kidney/Art*Art.Cold) - (F_Kidney/KidneyVas*KidneyVas.Cold) - (GFR*phi/KidneyVas*KidneyVas.Cold) + (GFR*phi*(1-f_exc)/KidneyIntern*KidneyIntera.Cold)
41	d(KidneyVas.AlbHot)/dt = (k_on_toAlb*KidneyVas.Hot*KidneyVas.Albumin/KidneyVas-k_off_toAlb*KidneyVas.AlbHot) - (lambdaPhys*KidneyVas.AlbHot) + (F_Kidney/Art*Art.AlbHot) - (F_Kidney/KidneyVas*KidneyVas.AlbHot)
42	d(KidneyVas.AlbCold)/dt = (lambdaPhys*KidneyVas.AlbHot) + (k_on_toAlb*KidneyVas.Cold*KidneyVas.Albumin/KidneyVas-k_off_toAlb*KidneyVas.AlbCold) + (F_Kidney/Art*Art.AlbCold) - (F_Kidney/KidneyVas*KidneyVas.AlbCold)
43	d(KidneyVas.Albumin)/dt = -(k_on_toAlb*KidneyVas.Hot*KidneyVas.Albumin/KidneyVas-k_off_toAlb*KidneyVas.AlbHot) - (k_on_toAlb*KidneyVas.Cold*KidneyVas.Albumin/KidneyVas-k_off_toAlb*KidneyVas.AlbCold) + (F_Kidney/Art*Art.Albumin) - (F_Kidney/KidneyVas*KidneyVas.Albumin)
44	d(KidneyInt.Hot)/dt = -(k_on/KidneyInt*KidneyInt.Hot*Kidney.R-k_off*KidneyBound.Hot) - (lambdaPhys*KidneyInt.Hot) + (GFR*phi/KidneyVas*KidneyVas.Hot) - (GFR*phi*(1-f_exc)/KidneyInt*KidneyInt.Hot) - (GFR*phi*f_exc/KidneyInt*KidneyInt.Hot)
45	d(KidneyInt.Cold)/dt = -(k_on/KidneyInt*KidneyInt.Cold*Kidney.R-k_off*KidneyBound.Cold) + (lambdaPhys*KidneyInt.Hot) + (GFR*phi/KidneyVas*KidneyVas.Cold) - (GFR*phi*(1-f_exc)/KidneyInt*KidneyInt.Cold) - (GFR*phi*f_exc/KidneyInt*KidneyInt.Cold)
46	d(KidneyBound.Hot)/dt = (k_on/KidneyInt*KidneyInt.Hot*Kidney.R-k_off*KidneyBound.Hot) - (lambdaIntern_Kidney*KidneyBound.Hot) - (lambdaPhys*KidneyBound.Hot)
47	d(KidneyBound.Cold)/dt = (k_on/KidneyInt*KidneyInt.Cold*Kidney.R-k_off*KidneyBound.Cold) - (lambdaIntern_Kidney*KidneyBound.Cold) + (lambdaPhys*KidneyBound.Hot)
48	d(KidneyIntern.Hot)/dt = (lambdaIntern_Kidney*KidneyBound.Hot) - (lambdaPhys*KidneyIntern.Hot) -

	(lambdaRel_Kidney*KidneyIntern.Hot)
49	d(KidneyIntern.Cold)/dt = (lambdaIntern_Kidney*KidneyBound.Cold) + (lambdaPhys*KidneyIntern.Hot) - (lambdaRel_Kidney*KidneyIntern.Cold)
50	d(KidneyIntera.Hot)/dt = (GFR*phi*(1-f_exc)/KidneyInt*KidneyInt.Hot) - (GFR*phi*(1-f_exc)/KidneyIntern*KidneyIntera.Hot)
51	d(KidneyIntera.Cold)/dt = (GFR*phi*(1-f_exc)/KidneyInt*KidneyInt.Cold) - (GFR*phi*(1-f_exc)/KidneyIntern*KidneyIntera.Cold)
52	d(HeartVas.Hot)/dt = -(lambdaPhys*HeartVas.Hot) - (k_on_toAlb*HeartVas.Hot*HeartVas.Albumin/HeartVas-k_off_toAlb*HeartVas.AlbHot) - (PS_Heart/HeartVas*HeartVas.Hot-PS_Heart/HeartInt*HeartInt.Hot) + (F_Heart/Art*Art.Hot) - (F_Heart/HeartVas*HeartVas.Hot)
53	d(HeartVas.Cold)/dt = (lambdaPhys*HeartVas.Hot) - (k_on_toAlb*HeartVas.Cold*HeartVas.Albumin/HeartVas-k_off_toAlb*HeartVas.AlbCold) - (PS_Heart/HeartVas*HeartVas.Cold-PS_Heart/HeartInt*HeartInt.Cold) + (F_Heart/Art*Art.Cold) - (F_Heart/HeartVas*HeartVas.Cold)
54	d(HeartVas.AlbHot)/dt = (k_on_toAlb*HeartVas.Hot*HeartVas.Albumin/HeartVas-k_off_toAlb*HeartVas.AlbHot) - (lambdaPhys*HeartVas.AlbHot) + (F_Heart/Art*Art.AlbHot) - (F_Heart/HeartVas*HeartVas.AlbHot)
55	d(HeartVas.AlbCold)/dt = (lambdaPhys*HeartVas.AlbHot) + (k_on_toAlb*HeartVas.Cold*HeartVas.Albumin/HeartVas-k_off_toAlb*HeartVas.AlbCold) + (F_Heart/Art*Art.AlbCold) - (F_Heart/HeartVas*HeartVas.AlbCold)
56	d(HeartVas.Albumin)/dt = -(k_on_toAlb*HeartVas.Hot*HeartVas.Albumin/HeartVas-k_off_toAlb*HeartVas.AlbHot) - (k_on_toAlb*HeartVas.Cold*HeartVas.Albumin/HeartVas-k_off_toAlb*HeartVas.AlbCold) + (F_Heart/Art*Art.Albumin) - (F_Heart/HeartVas*HeartVas.Albumin)
57	d(HeartInt.Hot)/dt = (PS_Heart/HeartVas*HeartVas.Hot-PS_Heart/HeartInt*HeartInt.Hot) - (lambdaPhys*HeartInt.Hot)
58	d(HeartInt.Cold)/dt = (PS_Heart/HeartVas*HeartVas.Cold-PS_Heart/HeartInt*HeartInt.Cold) + (lambdaPhys*HeartInt.Hot)
59	d(SGVas.Hot)/dt = -(lambdaPhys*SGVas.Hot) - (k_on_toAlb*SGVas.Hot*SGVas.Albumin/SGVas-k_off_toAlb*SGVas.AlbHot) - (PS_SG/SGVas*SGVas.Hot-PS_SG/SGInt*SGInt.Hot) + (F_SG/Art*Art.Hot) - (F_SG/SGVas*SGVas.Hot)
60	d(SGVas.Cold)/dt = (lambdaPhys*SGVas.Hot) - (k_on_toAlb*SGVas.Cold*SGVas.Albumin/SGVas-k_off_toAlb*SGVas.AlbCold) - (PS_SG/SGVas*SGVas.Cold-PS_SG/SGInt*SGInt.Cold) + (F_SG/Art*Art.Cold) - (F_SG/SGVas*SGVas.Cold)
61	d(SGVas.AlbHot)/dt = (k_on_toAlb*SGVas.Hot*SGVas.Albumin/SGVas-k_off_toAlb*SGVas.AlbHot) - (lambdaPhys*SGVas.AlbHot) + (F_SG/Art*Art.AlbHot) - (F_SG/SGVas*SGVas.AlbHot)
62	d(SGVas.AlbCold)/dt = (lambdaPhys*SGVas.AlbHot) + (k_on_toAlb*SGVas.Cold*SGVas.Albumin/SGVas-k_off_toAlb*SGVas.AlbCold) + (F_SG/Art*Art.AlbCold) - (F_SG/SGVas*SGVas.AlbCold)
63	d(SGVas.Albumin)/dt = -(k_on_toAlb*SGVas.Hot*SGVas.Albumin/SGVas-k_off_toAlb*SGVas.AlbHot) - (k_on_toAlb*SGVas.Cold*SGVas.Albumin/SGVas-k_off_toAlb*SGVas.AlbCold) + (F_SG/Art*Art.Albumin) - (F_SG/SGVas*SGVas.Albumin)
64	d(SGInt.Hot)/dt = (PS_SG/SGVas*SGVas.Hot-PS_SG/SGInt*SGInt.Hot) - (k_on/SGInt*SGInt.Hot*SG.R-k_off*SGBound.Hot) - (lambdaPhys*SGInt.Hot)
65	d(SGInt.Cold)/dt = (PS_SG/SGVas*SGVas.Cold-PS_SG/SGInt*SGInt.Cold) - (k_on/SGInt*SGInt.Cold*SG.R-k_off*SGBound.Cold) + (lambdaPhys*SGInt.Hot)
66	d(SGBound.Hot)/dt = (k_on/SGInt*SGInt.Hot*SG.R-k_off*SGBound.Hot) - (lambdaIntern_SG*SGBound.Hot) - (lambdaPhys*SGBound.Hot)
67	d(SGBound.Cold)/dt = (k_on/SGInt*SGInt.Cold*SG.R-k_off*SGBound.Cold) - (lambdaIntern_SG*SGBound.Cold) + (lambdaPhys*SGBound.Hot)
68	d(SGIntern.Hot)/dt = (lambdaIntern_SG*SGBound.Hot) - (lambdaPhys*SGIntern.Hot) - (lambdaRel_SG*SGIntern.Hot)
69	d(SGIntern.Cold)/dt = (lambdaIntern_SG*SGBound.Cold) + (lambdaPhys*SGIntern.Hot) - (lambdaRel_SG*SGIntern.Cold)
70	d(BoneVas.Hot)/dt = -(lambdaPhys*BoneVas.Hot) - (k_on_toAlb*BoneVas.Hot*BoneVas.Albumin/BoneVas-k_off_toAlb*BoneVas.AlbHot) - (PS_Bone/BoneVas*BoneVas.Hot-PS_Bone/BoneInt*BoneInt.Hot) + (F_Bone/Art*Art.Hot) - (F_Bone/BoneVas*BoneVas.Hot)
71	d(BoneVas.Cold)/dt = (lambdaPhys*BoneVas.Hot) - (k_on_toAlb*BoneVas.Cold*BoneVas.Albumin/BoneVas-k_off_toAlb*BoneVas.AlbCold) - (PS_Bone/BoneVas*BoneVas.Cold-PS_Bone/BoneInt*BoneInt.Cold) + (F_Bone/Art*Art.Cold) - (F_Bone/BoneVas*BoneVas.Cold)
72	d(BoneVas.AlbHot)/dt = (k_on_toAlb*BoneVas.Hot*BoneVas.Albumin/BoneVas-k_off_toAlb*BoneVas.AlbHot) - (lambdaPhys*BoneVas.AlbHot) + (F_Bone/Art*Art.AlbHot) - (F_Bone/BoneVas*BoneVas.AlbHot)
73	d(BoneVas.AlbCold)/dt = (lambdaPhys*BoneVas.AlbHot) + (k_on_toAlb*BoneVas.Cold*BoneVas.Albumin/BoneVas-k_off_toAlb*BoneVas.AlbCold) + (F_Bone/Art*Art.AlbCold) - (F_Bone/BoneVas*BoneVas.AlbCold)
74	d(BoneVas.Albumin)/dt = -(k_on_toAlb*BoneVas.Hot*BoneVas.Albumin/BoneVas-k_off_toAlb*BoneVas.AlbHot) - (k_on_toAlb*BoneVas.Cold*BoneVas.Albumin/BoneVas-k_off_toAlb*BoneVas.AlbCold) + (F_Bone/Art*Art.Albumin) - (F_Bone/BoneVas*BoneVas.Albumin)
75	d(BoneInt.Hot)/dt = (PS_Bone/BoneVas*BoneVas.Hot-PS_Bone/BoneInt*BoneInt.Hot) - (lambdaPhys*BoneInt.Hot)
76	d(BoneInt.Cold)/dt = (PS_Bone/BoneVas*BoneVas.Cold-PS_Bone/BoneInt*BoneInt.Cold) + (lambdaPhys*BoneInt.Hot)
77	d(TumorRestVas.Hot)/dt = -(lambdaPhys*TumorRestVas.Hot) - (k_on_toAlb*TumorRestVas.Hot*TumorRestVas.Albumin/TumorRestVas-k_off_toAlb*TumorRestVas.AlbHot) - (PS_TumorRest/TumorRestVas*TumorRestVas.Hot-PS_TumorRest/TumorRestInt*TumorRestInt.Hot) + (F_TumorRest/Art*Art.Hot) - (F_TumorRest/TumorRestVas*TumorRestVas.Hot)
78	d(TumorRestVas.Cold)/dt = (lambdaPhys*TumorRestVas.Hot) - (k_on_toAlb*TumorRestVas.Cold*TumorRestVas.Albumin/TumorRestVas-k_off_toAlb*TumorRestVas.AlbCold) - (PS_TumorRest/TumorRestVas*TumorRestVas.Cold-PS_TumorRest/TumorRestInt*TumorRestInt.Cold) + (F_TumorRest/Art*Art.Cold) - (F_TumorRest/TumorRestVas*TumorRestVas.Cold)
79	d(TumorRestVas.AlbHot)/dt = (k_on_toAlb*TumorRestVas.Hot*TumorRestVas.Albumin/TumorRestVas-k_off_toAlb*TumorRestVas.AlbHot) - (lambdaPhys*TumorRestVas.AlbHot) - (PSAlb_TumorRest/TumorRestVas*TumorRestVas.AlbHot-PSAlb_TumorRest/TumorRestInt*TumorRestInt.AlbHot) + (F_TumorRest/Art*Art.AlbHot) - (F_TumorRest/TumorRestVas*TumorRestVas.AlbHot)
80	d(TumorRestVas.AlbCold)/dt = (lambdaPhys*TumorRestVas.AlbHot) + (k_on_toAlb*TumorRestVas.Cold*TumorRestVas.Albumin/TumorRestVas-k_off_toAlb*TumorRestVas.AlbCold) - (PSAlb_TumorRest/TumorRestVas*TumorRestVas.AlbCold-PSAlb_TumorRest/TumorRestInt*TumorRestInt.AlbCold) + (F_TumorRest/Art*Art.AlbCold) - (F_TumorRest/TumorRestVas*TumorRestVas.AlbCold)
81	d(TumorRestVas.Albumin)/dt = -(k_on_toAlb*TumorRestVas.Hot*TumorRestVas.Albumin/TumorRestVas-k_off_toAlb*TumorRestVas.AlbHot) - (k_on_toAlb*TumorRestVas.Cold*TumorRestVas.Albumin/TumorRestVas-k_off_toAlb*TumorRestVas.AlbCold) + (F_TumorRest/Art*Art.Albumin) - (F_TumorRest/TumorRestVas*TumorRestVas.Albumin) - (PSAlb_TumorRest/TumorRestVas*TumorRestVas.Albumin-PSAlb_TumorRest/TumorRestInt*TumorRestInt.Albumin)

82	d(TumorRestInt.Hot)/dt = (PS_TumorRest/TumorRestVas*TumorRestVas.Hot-PS_TumorRest/TumorRestInt*TumorRestInt.Hot) - (k_on/TumorRestInt*TumorRestInt.Hot*TumorRest.R-k_off*TumorRestBound.Hot) - (lambdaPhys*TumorRestInt.Hot) - (k_on_toAlb*TumorRestInt.Hot*TumorRestInt.Albumin/TumorRestInt-k_off_toAlb*TumorRestInt.AlbHot)
83	d(TumorRestInt.Cold)/dt = (PS_TumorRest/TumorRestVas*TumorRestVas.Cold-PS_TumorRest/TumorRestInt*TumorRestInt.Cold) - (k_on/TumorRestInt*TumorRestInt.Cold*TumorRest.R-k_off*TumorRestBound.Cold) + (lambdaPhys*TumorRestInt.Hot) - (k_on_toAlb*TumorRestInt.Cold*TumorRestInt.Albumin/TumorRestInt-k_off_toAlb*TumorRestInt.AlbCold)
84	d(TumorRestInt.AlbHot)/dt = (PSAlb_TumorRest/TumorRestVas*TumorRestVas.AlbHot-PSAlb_TumorRest/TumorRestInt*TumorRestInt.AlbHot) + (k_on_toAlb*TumorRestInt.Hot*TumorRestInt.Albumin/TumorRestInt-k_off_toAlb*TumorRestInt.AlbHot) - (lambdaPhys*TumorRestInt.AlbHot)
85	d(TumorRestInt.AlbCold)/dt = (PSAlb_TumorRest/TumorRestVas*TumorRestVas.AlbCold-PSAlb_TumorRest/TumorRestInt*TumorRestInt.AlbCold) + (lambdaPhys*TumorRestInt.AlbHot) + (k_on_toAlb*TumorRestInt.Cold*TumorRestInt.Albumin/TumorRestInt-k_off_toAlb*TumorRestInt.AlbCold)
86	d(TumorRestInt.Albumin)/dt = -(k_on_toAlb*TumorRestInt.Hot*TumorRestInt.Albumin/TumorRestInt-k_off_toAlb*TumorRestInt.AlbHot) - (k_on_toAlb*TumorRestInt.Cold*TumorRestInt.Albumin/TumorRestInt-k_off_toAlb*TumorRestInt.AlbCold) + (PSAlb_TumorRest/TumorRestVas*TumorRestVas.Albumin-PSAlb_TumorRest/TumorRestInt*TumorRestInt.Albumin)
87	d(TumorRestBound.Hot)/dt = (k_on/TumorRestInt*TumorRestInt.Hot*TumorRest.R-k_off*TumorRestBound.Hot) - (lambdaIntern_TumorRest*TumorRestBound.Hot) - (lambdaPhys*TumorRestBound.Hot)
88	d(TumorRestBound.Cold)/dt = (k_on/TumorRestInt*TumorRestInt.Cold*TumorRest.R-k_off*TumorRestBound.Cold) - (lambdaIntern_TumorRest*TumorRestBound.Cold) + (lambdaPhys*TumorRestBound.Hot)
89	d(TumorRestIntern.Hot)/dt = (lambdaIntern_TumorRest*TumorRestBound.Hot) - (lambdaPhys*TumorRestIntern.Hot) - (lambdaRel_TumorRest*TumorRestIntern.Hot)
90	d(TumorRestIntern.Cold)/dt = (lambdaIntern_TumorRest*TumorRestBound.Cold) + (lambdaPhys*TumorRestIntern.Hot) - (lambdaRel_TumorRest*TumorRestIntern.Cold)
91	d(SpleenVas.Hot)/dt = -(lambdaPhys*SpleenVas.Hot) - (k_on_toAlb*SpleenVas.Hot*SpleenVas.Albumin/SpleenVas-k_off_toAlb*SpleenVas.AlbHot) - (PS_Spleen/SpleenVas*SpleenVas.Hot-PS_Spleen/SpleenInt*SpleenInt.Hot) + (F_Spleen/Art*Art.Hot) - (F_Spleen/SpleenVas*SpleenVas.Hot)
92	d(SpleenVas.Cold)/dt = (lambdaPhys*SpleenVas.Hot) - (k_on_toAlb*SpleenVas.Cold*SpleenVas.Albumin/SpleenVas-k_off_toAlb*SpleenVas.AlbCold) - (PS_Spleen/SpleenVas*SpleenVas.Cold-PS_Spleen/SpleenInt*SpleenInt.Cold) + (F_Spleen/Art*Art.Cold) - (F_Spleen/SpleenVas*SpleenVas.Cold)
93	d(SpleenVas.AlbHot)/dt = (k_on_toAlb*SpleenVas.Hot*SpleenVas.Albumin/SpleenVas-k_off_toAlb*SpleenVas.AlbHot) - (lambdaPhys*SpleenVas.AlbHot) + (F_Spleen/Art*Art.AlbHot) - (F_Spleen/SpleenVas*SpleenVas.AlbHot)
94	d(SpleenVas.AlbCold)/dt = (lambdaPhys*SpleenVas.AlbHot) + (k_on_toAlb*SpleenVas.Cold*SpleenVas.Albumin/SpleenVas-k_off_toAlb*SpleenVas.AlbCold) + (F_Spleen/Art*Art.AlbCold) - (F_Spleen/SpleenVas*SpleenVas.AlbCold)
95	d(SpleenVas.Albumin)/dt = -(k_on_toAlb*SpleenVas.Hot*SpleenVas.Albumin/SpleenVas-k_off_toAlb*SpleenVas.AlbHot) - (k_on_toAlb*SpleenVas.Cold*SpleenVas.Albumin/SpleenVas-k_off_toAlb*SpleenVas.AlbCold) + (F_Spleen/Art*Art.Albumin) - (F_Spleen/SpleenVas*SpleenVas.Albumin)
96	d(SpleenInt.Hot)/dt = (PS_Spleen/SpleenVas*SpleenVas.Hot-PS_Spleen/SpleenInt*SpleenInt.Hot) - (k_on/SpleenInt*SpleenInt.Hot*Spleen.R-k_off*SpleenBound.Hot) - (lambdaPhys*SpleenInt.Hot)
97	d(SpleenInt.Cold)/dt = (PS_Spleen/SpleenVas*SpleenVas.Cold-PS_Spleen/SpleenInt*SpleenInt.Cold) - (k_on/SpleenInt*SpleenInt.Cold*Spleen.R-k_off*SpleenBound.Cold) + (lambdaPhys*SpleenInt.Hot)
98	d(SpleenBound.Hot)/dt = (k_on/SpleenInt*SpleenInt.Hot*Spleen.R-k_off*SpleenBound.Hot) - (lambdaIntern_Spleen*SpleenBound.Hot) - (lambdaPhys*SpleenBound.Hot)
99	d(SpleenBound.Cold)/dt = (k_on/SpleenInt*SpleenInt.Cold*Spleen.R-k_off*SpleenBound.Cold) - (lambdaIntern_Spleen*SpleenBound.Cold) + (lambdaPhys*SpleenBound.Hot)
100	d(SpleenIntern.Hot)/dt = (lambdaIntern_Spleen*SpleenBound.Hot) - (lambdaPhys*SpleenIntern.Hot) - (lambdaRel_Spleen*SpleenIntern.Hot)
101	d(SpleenIntern.Cold)/dt = (lambdaIntern_Spleen*SpleenBound.Cold) + (lambdaPhys*SpleenIntern.Hot) - (lambdaRel_Spleen*SpleenIntern.Cold)
102	d(LiverVas.Hot)/dt = -(lambdaPhys*LiverVas.Hot) - (k_on_toAlb*LiverVas.Hot*LiverVas.Albumin/LiverVas-k_off_toAlb*LiverVas.AlbHot) - (PS_Liver/LiverVas*LiverVas.Hot-PS_Liver/LiverInt*LiverInt.Hot) + (F_Liver/Art*Art.Hot) - (F_Liver/LiverVas*LiverVas.Hot) + (F_Spleen/SpleenVas*SpleenVas.Hot) + (F_GI/GIVas*GIVas.Hot)
103	d(LiverVas.Cold)/dt = (lambdaPhys*LiverVas.Hot) - (k_on_toAlb*LiverVas.Cold*LiverVas.Albumin/LiverVas-k_off_toAlb*LiverVas.AlbCold) - (PS_Liver/LiverVas*LiverVas.Cold-PS_Liver/LiverInt*LiverInt.Cold) + (F_Liver/Art*Art.Cold) - (F_Liver/LiverVas*LiverVas.Cold) + (F_Spleen/SpleenVas*SpleenVas.Cold) + (F_GI/GIVas*GIVas.Cold)
104	d(LiverVas.AlbHot)/dt = (k_on_toAlb*LiverVas.Hot*LiverVas.Albumin/LiverVas-k_off_toAlb*LiverVas.AlbHot) - (lambdaPhys*LiverVas.AlbHot) + (F_Liver/Art*Art.AlbHot) - (F_Liver/LiverVas*LiverVas.AlbHot) + (F_Spleen/SpleenVas*SpleenVas.AlbHot) + (F_GI/GIVas*GIVas.AlbHot)
105	d(LiverVas.AlbCold)/dt = (lambdaPhys*LiverVas.AlbHot) + (k_on_toAlb*LiverVas.Cold*LiverVas.Albumin/LiverVas-k_off_toAlb*LiverVas.AlbCold) + (F_Liver/Art*Art.AlbCold) - (F_Liver/LiverVas*LiverVas.AlbCold) + (F_Spleen/SpleenVas*SpleenVas.AlbCold) + (F_GI/GIVas*GIVas.AlbCold)
106	d(LiverVas.Albumin)/dt = -(k_on_toAlb*LiverVas.Hot*LiverVas.Albumin/LiverVas-k_off_toAlb*LiverVas.AlbHot) - (k_on_toAlb*LiverVas.Cold*LiverVas.Albumin/LiverVas-k_off_toAlb*LiverVas.AlbCold) + (F_Liver/Art*Art.Albumin) - (F_Liver/LiverVas*LiverVas.Albumin)
107	d(LiverInt.Hot)/dt = (PS_Liver/LiverVas*LiverVas.Hot-PS_Liver/LiverInt*LiverInt.Hot) - (k_on/LiverInt*LiverInt.Hot*Liver.R-k_off*LiverBound.Hot) - (lambdaPhys*LiverInt.Hot)
108	d(LiverInt.Cold)/dt = (PS_Liver/LiverVas*LiverVas.Cold-PS_Liver/LiverInt*LiverInt.Cold) - (k_on/LiverInt*LiverInt.Cold*Liver.R-k_off*LiverBound.Cold) + (lambdaPhys*LiverInt.Hot)
109	d(LiverBound.Hot)/dt = (k_on/LiverInt*LiverInt.Hot*Liver.R-k_off*LiverBound.Hot) - (lambdaIntern_Liver*LiverBound.Hot) - (lambdaPhys*LiverBound.Hot)
110	d(LiverBound.Cold)/dt = (k_on/LiverInt*LiverInt.Cold*Liver.R-k_off*LiverBound.Cold) - (lambdaIntern_Liver*LiverBound.Cold) + (lambdaPhys*LiverBound.Hot)
111	d(LiverIntern.Hot)/dt = (lambdaIntern_Liver*LiverBound.Hot) - (lambdaPhys*LiverIntern.Hot) - (lambdaRel_Liver*LiverIntern.Hot)

112	d(LiverIntern.Cold)/dt = (lambdaIntern_Liver*LiverBound.Cold) + (lambdaPhys*LiverIntern.Hot) - (lambdaRel_Liver*LiverIntern.Cold)
113	d(ProstateVas.Hot)/dt = -(lambdaPhys*ProstateVas.Hot) - (k_on_toAlb*ProstateVas.Hot*ProstateVas.Albumin/ProstateVas-k_off_toAlb*ProstateVas.AlbHot) - (PS_Prostate/ProstateVas*ProstateVas.Hot-PS_Prostate/ProstateInt*ProstateInt.Hot) + (F_Prostate/Art*Art.Hot) - (F_Prostate/ProstateVas*ProstateVas.Hot)
114	d(ProstateVas.Cold)/dt = (lambdaPhys*ProstateVas.Hot) - (k_on_toAlb*ProstateVas.Cold*ProstateVas.Albumin/ProstateVas-k_off_toAlb*ProstateVas.AlbCold) - (PS_Prostate/ProstateVas*ProstateVas.Cold-PS_Prostate/ProstateInt*ProstateInt.Cold) + (F_Prostate/Art*Art.Cold) - (F_Prostate/ProstateVas*ProstateVas.Cold)
115	d(ProstateVas.AlbHot)/dt = (k_on_toAlb*ProstateVas.Hot*ProstateVas.Albumin/ProstateVas-k_off_toAlb*ProstateVas.AlbHot) - (lambdaPhys*ProstateVas.AlbHot) + (F_Prostate/Art*Art.AlbHot) - (F_Prostate/ProstateVas*ProstateVas.AlbHot)
116	d(ProstateVas.AlbCold)/dt = (lambdaPhys*ProstateVas.AlbHot) + (k_on_toAlb*ProstateVas.Cold*ProstateVas.Albumin/ProstateVas-k_off_toAlb*ProstateVas.AlbCold) + (F_Prostate/Art*Art.AlbCold) - (F_Prostate/ProstateVas*ProstateVas.AlbCold)
117	d(ProstateVas.Albumin)/dt = -(k_on_toAlb*ProstateVas.Hot*ProstateVas.Albumin/ProstateVas-k_off_toAlb*ProstateVas.AlbHot) - (k_on_toAlb*ProstateVas.Cold*ProstateVas.Albumin/ProstateVas-k_off_toAlb*ProstateVas.AlbCold) + (F_Prostate/Art*Art.Albumin) - (F_Prostate/ProstateVas*ProstateVas.Albumin)
118	d(ProstateInt.Hot)/dt = (PS_Prostate/ProstateVas*ProstateVas.Hot-PS_Prostate/ProstateInt*ProstateInt.Hot) - (k_on/ProstateInt*ProstateInt.Hot*Prostate.R-k_off*ProstateBound.Hot) - (lambdaPhys*ProstateInt.Hot)
119	d(ProstateInt.Cold)/dt = (PS_Prostate/ProstateVas*ProstateVas.Cold-PS_Prostate/ProstateInt*ProstateInt.Cold) - (k_on/ProstateInt*ProstateInt.Cold*Prostate.R-k_off*ProstateBound.Cold) + (lambdaPhys*ProstateInt.Hot)
120	d(ProstateBound.Hot)/dt = (k_on/ProstateInt*ProstateInt.Hot*Prostate.R-k_off*ProstateBound.Hot) - (lambdaIntern_Prostate*ProstateBound.Hot) - (lambdaPhys*ProstateBound.Hot)
121	d(ProstateBound.Cold)/dt = (k_on/ProstateInt*ProstateInt.Cold*Prostate.R-k_off*ProstateBound.Cold) - (lambdaIntern_Prostate*ProstateBound.Cold) + (lambdaPhys*ProstateBound.Hot)
122	d(ProstateIntern.Hot)/dt = (lambdaIntern_Prostate*ProstateBound.Hot) - (lambdaPhys*ProstateIntern.Hot) - (lambdaRel_Prostate*ProstateIntern.Hot)
123	d(ProstateIntern.Cold)/dt = (lambdaIntern_Prostate*ProstateBound.Cold) + (lambdaPhys*ProstateIntern.Hot) - (lambdaRel_Prostate*ProstateIntern.Cold)
124	d(GIVas.Hot)/dt = -(lambdaPhys*GIVas.Hot) - (k_on_toAlb*GIVas.Hot*GIVas.Albumin/GIVas-k_off_toAlb*GIVas.AlbHot) - (PS_GI/GIVas*GIVas.Hot-PS_GI/GIInt*GIInt.Hot) + (F_GI/Art*Art.Hot) - (F_GI/GIVas*GIVas.Hot)
125	d(GIVas.Cold)/dt = (lambdaPhys*GIVas.Hot) - (k_on_toAlb*GIVas.Cold*GIVas.Albumin/GIVas-k_off_toAlb*GIVas.AlbCold) - (PS_GI/GIVas*GIVas.Cold-PS_GI/GIInt*GIInt.Cold) + (F_GI/Art*Art.Cold) - (F_GI/GIVas*GIVas.Cold)
126	d(GIVas.AlbHot)/dt = (k_on_toAlb*GIVas.Hot*GIVas.Albumin/GIVas-k_off_toAlb*GIVas.AlbHot) - (lambdaPhys*GIVas.AlbHot) + (F_GI/Art*Art.AlbHot) - (F_GI/GIVas*GIVas.AlbHot)
127	d(GIVas.AlbCold)/dt = (lambdaPhys*GIVas.AlbHot) + (k_on_toAlb*GIVas.Cold*GIVas.Albumin/GIVas-k_off_toAlb*GIVas.AlbCold) + (F_GI/Art*Art.AlbCold) - (F_GI/GIVas*GIVas.AlbCold)
128	d(GIVas.Albumin)/dt = -(k_on_toAlb*GIVas.Hot*GIVas.Albumin/GIVas-k_off_toAlb*GIVas.AlbHot) - (k_on_toAlb*GIVas.Cold*GIVas.Albumin/GIVas-k_off_toAlb*GIVas.AlbCold) + (F_GI/Art*Art.Albumin) - (F_GI/GIVas*GIVas.Albumin)
129	d(GIInt.Hot)/dt = (PS_GI/GIVas*GIVas.Hot-PS_GI/GIInt*GIInt.Hot) - (k_on/GIInt*GIInt.Hot*GI.R-k_off*GIBound.Hot) - (lambdaPhys*GIInt.Hot)
130	d(GIInt.Cold)/dt = (PS_GI/GIVas*GIVas.Cold-PS_GI/GIInt*GIInt.Cold) - (k_on/GIInt*GIInt.Cold*GI.R-k_off*GIBound.Cold) + (lambdaPhys*GIInt.Hot)
131	d(GIBound.Hot)/dt = (k_on/GIInt*GIInt.Hot*GI.R-k_off*GIBound.Hot) - (lambdaIntern_GI*GIBound.Hot) - (lambdaPhys*GIBound.Hot)
132	d(GIBound.Cold)/dt = (k_on/GIInt*GIInt.Cold*GI.R-k_off*GIBound.Cold) - (lambdaIntern_GI*GIBound.Cold) + (lambdaPhys*GIBound.Hot)
133	d(GIIntern.Hot)/dt = (lambdaIntern_GI*GIBound.Hot) - (lambdaPhys*GIIntern.Hot) - (lambdaRel_GI*GIIntern.Hot)
134	d(GIIntern.Cold)/dt = (lambdaIntern_GI*GIBound.Cold) + (lambdaPhys*GIIntern.Hot) - (lambdaRel_GI*GIIntern.Cold)
135	d(RestVas.Hot)/dt = -(lambdaPhys*RestVas.Hot) - (k_on_toAlb*RestVas.Hot*RestVas.Albumin/RestVas-k_off_toAlb*RestVas.AlbHot) - (PS_Rest/RestVas*RestVas.Hot-PS_Rest/RestInt*RestInt.Hot) + (F_Rest/Art*Art.Hot) - (F_Rest/RestVas*RestVas.Hot)
136	d(RestVas.Cold)/dt = (lambdaPhys*RestVas.Hot) - (k_on_toAlb*RestVas.Cold*RestVas.Albumin/RestVas-k_off_toAlb*RestVas.AlbCold) - (PS_Rest/RestVas*RestVas.Cold-PS_Rest/RestInt*RestInt.Cold) + (F_Rest/Art*Art.Cold) - (F_Rest/RestVas*RestVas.Cold)
137	d(RestVas.AlbHot)/dt = (k_on_toAlb*RestVas.Hot*RestVas.Albumin/RestVas-k_off_toAlb*RestVas.AlbHot) - (lambdaPhys*RestVas.AlbHot) + (F_Rest/Art*Art.AlbHot) - (F_Rest/RestVas*RestVas.AlbHot)
138	d(RestVas.AlbCold)/dt = (lambdaPhys*RestVas.AlbHot) + (k_on_toAlb*RestVas.Cold*RestVas.Albumin/RestVas-k_off_toAlb*RestVas.AlbCold) + (F_Rest/Art*Art.AlbCold) - (F_Rest/RestVas*RestVas.AlbCold)
139	d(RestVas.Albumin)/dt = -(k_on_toAlb*RestVas.Hot*RestVas.Albumin/RestVas-k_off_toAlb*RestVas.AlbHot) - (k_on_toAlb*RestVas.Cold*RestVas.Albumin/RestVas-k_off_toAlb*RestVas.AlbCold) + (F_Rest/Art*Art.Albumin) - (F_Rest/RestVas*RestVas.Albumin)
140	d(RestInt.Hot)/dt = (PS_Rest/RestVas*RestVas.Hot-PS_Rest/RestInt*RestInt.Hot) - (lambdaPhys*RestInt.Hot)
141	d(RestInt.Cold)/dt = (PS_Rest/RestVas*RestVas.Cold-PS_Rest/RestInt*RestInt.Cold) + (lambdaPhys*RestInt.Hot)
142	d(SkinVas.Hot)/dt = -(lambdaPhys*SkinVas.Hot) - (k_on_toAlb*SkinVas.Hot*SkinVas.Albumin/SkinVas-k_off_toAlb*SkinVas.AlbHot) - (PS_Skin/SkinVas*SkinVas.Hot-PS_Skin/SkinVas*SkinInt.Hot) + (F_Skin/Art*Art.Hot) - (F_Skin/SkinVas*SkinVas.Hot)
143	d(SkinVas.Cold)/dt = (lambdaPhys*SkinVas.Hot) - (k_on_toAlb*SkinVas.Cold*SkinVas.Albumin/SkinVas-k_off_toAlb*SkinVas.AlbCold) - (PS_Skin/SkinVas*SkinVas.Cold-PS_Skin/SkinInt*SkinInt.Cold) + (F_Skin/Art*Art.Cold) - (F_Skin/SkinVas*SkinVas.Cold)
144	d(SkinVas.AlbHot)/dt = (k_on_toAlb*SkinVas.Hot*SkinVas.Albumin/SkinVas-k_off_toAlb*SkinVas.AlbHot) - (lambdaPhys*SkinVas.AlbHot) + (F_Skin/Art*Art.AlbHot) - (F_Skin/SkinVas*SkinVas.AlbHot)
145	d(SkinVas.AlbCold)/dt = (lambdaPhys*SkinVas.AlbHot) + (k_on_toAlb*SkinVas.Cold*SkinVas.Albumin/SkinVas-k_off_toAlb*SkinVas.AlbCold) + (F_Skin/Art*Art.AlbCold) - (F_Skin/SkinVas*SkinVas.AlbCold)
146	d(SkinVas.Albumin)/dt = -(k_on_toAlb*SkinVas.Hot*SkinVas.Albumin/SkinVas-k_off_toAlb*SkinVas.AlbHot) - (k_on_toAlb*SkinVas.Cold*SkinVas.Albumin/SkinVas-k_off_toAlb*SkinVas.AlbCold) + (F_Skin/Art*Art.Albumin) - (F_Skin/SkinVas*SkinVas.Albumin)

147	d(SkinInt.Hot)/dt = (PS_Skin/SkinVas*SkinVas.Hot-PS_Skin/SkinVas*SkinInt.Hot) - (lambdaPhys*SkinInt.Hot)
148	d(SkinInt.Cold)/dt = (PS_Skin/SkinVas*SkinVas.Cold-PS_Skin/SkinInt*SkinInt.Cold) + (lambdaPhys*SkinInt.Hot)
149	d(MuscleVas.Hot)/dt = -(lambdaPhys*MuscleVas.Hot) - (k_on_toAlb*MuscleVas.Hot*MuscleVas.Albumin/MuscleVas-k_off_toAlb*MuscleVas.AlbHot) - (PS_Muscle/MuscleVas*MuscleVas.Hot-PS_Muscle/MuscleInt*MuscleInt.Hot) + (F_Muscle/Art*Art.Hot) - (F_Muscle/MuscleVas*MuscleVas.Hot)
150	d(MuscleVas.Cold)/dt = (lambdaPhys*MuscleVas.Hot) - (k_on_toAlb*MuscleVas.Cold*MuscleVas.Albumin/MuscleVas-k_off_toAlb*MuscleVas.AlbCold) - (PS_Muscle/MuscleVas*MuscleVas.Cold-PS_Muscle/MuscleInt*MuscleInt.Cold) + (F_Muscle/Art*Art.Cold) - (F_Muscle/MuscleVas*MuscleVas.Cold)
151	d(MuscleVas.AlbHot)/dt = (k_on_toAlb*MuscleVas.Hot*MuscleVas.Albumin/MuscleVas-k_off_toAlb*MuscleVas.AlbHot) - (lambdaPhys*MuscleVas.AlbHot) + (F_Muscle/Art*Art.AlbHot) - (F_Muscle/MuscleVas*MuscleVas.AlbHot)
152	d(MuscleVas.AlbCold)/dt = (lambdaPhys*MuscleVas.AlbHot) + (k_on_toAlb*MuscleVas.Cold*MuscleVas.Albumin/MuscleVas-k_off_toAlb*MuscleVas.AlbCold) + (F_Muscle/Art*Art.AlbCold) - (F_Muscle/MuscleVas*MuscleVas.AlbCold)
153	d(MuscleVas.Albumin)/dt = -(k_on_toAlb*MuscleVas.Hot*MuscleVas.Albumin/MuscleVas-k_off_toAlb*MuscleVas.AlbHot) - (k_on_toAlb*MuscleVas.Cold*MuscleVas.Albumin/MuscleVas-k_off_toAlb*MuscleVas.AlbCold) + (F_Muscle/Art*Art.Albumin) - (F_Muscle/MuscleVas*MuscleVas.Albumin)
154	d(MuscleInt.Hot)/dt = (PS_Muscle/MuscleVas*MuscleVas.Hot-PS_Muscle/MuscleInt*MuscleInt.Hot) - (lambdaPhys*MuscleInt.Hot)
155	d(MuscleInt.Cold)/dt = (PS_Muscle/MuscleVas*MuscleVas.Cold-PS_Muscle/MuscleInt*MuscleInt.Cold) + (lambdaPhys*MuscleInt.Hot)
156	d(BrainVas.Hot)/dt = -(lambdaPhys*BrainVas.Hot) - (k_on_toAlb*BrainVas.Hot*BrainVas.Albumin/BrainVas-k_off_toAlb*BrainVas.AlbHot) - (PS_Brain/BrainVas*BrainVas.Hot-PS_Brain/BrainInt*BrainInt.Hot) + (F_Brain/Art*Art.Hot) - (F_Brain/BrainVas*BrainVas.Hot)
157	d(BrainVas.Cold)/dt = (lambdaPhys*BrainVas.Hot) - (k_on_toAlb*BrainVas.Cold*BrainVas.Albumin/BrainVas-k_off_toAlb*BrainVas.AlbCold) - (PS_Brain/BrainVas*BrainVas.Cold-PS_Brain/BrainInt*BrainInt.Cold) + (F_Brain/Art*Art.Cold) - (F_Brain/BrainVas*BrainVas.Cold)
158	d(BrainVas.AlbHot)/dt = (k_on_toAlb*BrainVas.Hot*BrainVas.Albumin/BrainVas-k_off_toAlb*BrainVas.AlbHot) - (lambdaPhys*BrainVas.AlbHot) + (F_Brain/Art*Art.AlbHot) - (F_Brain/BrainVas*BrainVas.AlbHot)
159	d(BrainVas.AlbCold)/dt = (lambdaPhys*BrainVas.AlbHot) + (k_on_toAlb*BrainVas.Cold*BrainVas.Albumin/BrainVas-k_off_toAlb*BrainVas.AlbCold) + (F_Brain/Art*Art.AlbCold) - (F_Brain/BrainVas*BrainVas.AlbCold)
160	d(BrainVas.Albumin)/dt = -(k_on_toAlb*BrainVas.Hot*BrainVas.Albumin/BrainVas-k_off_toAlb*BrainVas.AlbHot) - (k_on_toAlb*BrainVas.Cold*BrainVas.Albumin/BrainVas-k_off_toAlb*BrainVas.AlbCold) + (F_Brain/Art*Art.Albumin) - (F_Brain/BrainVas*BrainVas.Albumin)
161	d(BrainInt.Hot)/dt = (PS_Brain/BrainVas*BrainVas.Hot-PS_Brain/BrainInt*BrainInt.Hot) - (lambdaPhys*BrainInt.Hot)
162	d(BrainInt.Cold)/dt = (PS_Brain/BrainVas*BrainVas.Cold-PS_Brain/BrainInt*BrainInt.Cold) + (lambdaPhys*BrainInt.Hot)
163	d(RedMarrowVas.Hot)/dt = -(lambdaPhys*RedMarrowVas.Hot) - (k_on_toAlb*RedMarrowVas.Hot*RedMarrowVas.Albumin/RedMarrowVas-k_off_toAlb*RedMarrowVas.AlbHot) - (PS_RedMarrow/RedMarrowVas*RedMarrowVas.Hot-PS_RedMarrow/RedMarrowInt*RedMarrowInt.Hot) + (F_RedMarrow/Art*Art.Hot) - (F_RedMarrow/RedMarrowVas*RedMarrowVas.Hot)
164	d(RedMarrowVas.Cold)/dt = (lambdaPhys*RedMarrowVas.Hot) - (k_on_toAlb*RedMarrowVas.Cold*RedMarrowVas.Albumin/RedMarrowVas-k_off_toAlb*RedMarrowVas.AlbCold) - (PS_RedMarrow/RedMarrowVas*RedMarrowVas.Cold-PS_RedMarrow/RedMarrowInt*RedMarrowInt.Cold) + (F_RedMarrow/Art*Art.Cold) - (F_RedMarrow/RedMarrowVas*RedMarrowVas.Cold)
165	d(RedMarrowVas.AlbHot)/dt = (k_on_toAlb*RedMarrowVas.Hot*RedMarrowVas.Albumin/RedMarrowVas-k_off_toAlb*RedMarrowVas.AlbHot) - (lambdaPhys*RedMarrowVas.AlbHot) + (F_RedMarrow/Art*Art.AlbHot) - (F_RedMarrow/RedMarrowVas*RedMarrowVas.AlbHot)
166	d(RedMarrowVas.AlbCold)/dt = (lambdaPhys*RedMarrowVas.AlbHot) + (k_on_toAlb*RedMarrowVas.Cold*RedMarrowVas.Albumin/RedMarrowVas-k_off_toAlb*RedMarrowVas.AlbCold) + (F_RedMarrow/Art*Art.AlbCold) - (F_RedMarrow/RedMarrowVas*RedMarrowVas.AlbCold)
167	d(RedMarrowVas.Albumin)/dt = -(k_on_toAlb*RedMarrowVas.Hot*RedMarrowVas.Albumin/RedMarrowVas-k_off_toAlb*RedMarrowVas.AlbHot) - (k_on_toAlb*RedMarrowVas.Cold*RedMarrowVas.Albumin/RedMarrowVas-k_off_toAlb*RedMarrowVas.AlbCold) + (F_RedMarrow/Art*Art.Albumin) - (F_RedMarrow/RedMarrowVas*RedMarrowVas.Albumin)
168	d(RedMarrowInt.Hot)/dt = (PS_RedMarrow/RedMarrowVas*RedMarrowVas.Hot-PS_RedMarrow/RedMarrowInt*RedMarrowInt.Hot) - (lambdaPhys*RedMarrowInt.Hot)
169	d(RedMarrowInt.Cold)/dt = (PS_RedMarrow/RedMarrowVas*RedMarrowVas.Cold-PS_RedMarrow/RedMarrowInt*RedMarrowInt.Cold) + (lambdaPhys*RedMarrowInt.Hot)
170	d(LungsVas.Hot)/dt = -(lambdaPhys*LungsVas.Hot) - (k_on_toAlb*LungsVas.Hot*LungsVas.Albumin/LungsVas-k_off_toAlb*LungsVas.AlbHot) - (PS_Lungs/LungsVas*LungsVas.Hot-PS_Lungs/LungsInt*LungsInt.Hot) - (F_Lungs/LungsVas*LungsVas.Hot) + (F_Lungs/Vein*Vein.Hot)
171	d(LungsVas.Cold)/dt = (lambdaPhys*LungsVas.Hot) - (k_on_toAlb*LungsVas.Cold*LungsVas.Albumin/LungsVas-k_off_toAlb*LungsVas.AlbCold) - (PS_Lungs/LungsVas*LungsVas.Cold-PS_Lungs/LungsInt*LungsInt.Cold) - (F_Lungs/LungsVas*LungsVas.Cold) + (F_Lungs/Vein*Vein.Cold)
172	d(LungsVas.AlbHot)/dt = (k_on_toAlb*LungsVas.Hot*LungsVas.Albumin/LungsVas-k_off_toAlb*LungsVas.AlbHot) - (lambdaPhys*LungsVas.AlbHot) - (F_Lungs/LungsVas*LungsVas.AlbHot) + (F_Lungs/Vein*Vein.AlbHot)
173	d(LungsVas.AlbCold)/dt = (lambdaPhys*LungsVas.AlbHot) + (k_on_toAlb*LungsVas.Cold*LungsVas.Albumin/LungsVas-k_off_toAlb*LungsVas.AlbCold) - (F_Lungs/LungsVas*LungsVas.AlbCold) + (F_Lungs/Vein*Vein.AlbCold)
174	d(LungsVas.Albumin)/dt = -(k_on_toAlb*LungsVas.Hot*LungsVas.Albumin/LungsVas-k_off_toAlb*LungsVas.AlbHot) - (k_on_toAlb*LungsVas.Cold*LungsVas.Albumin/LungsVas-k_off_toAlb*LungsVas.AlbCold) + (F_Lungs/Vein*Vein.Albumin) - (F_Lungs/LungsVas*LungsVas.Albumin)
175	d(LungsInt.Hot)/dt = (PS_Lungs/LungsVas*LungsVas.Hot-PS_Lungs/LungsInt*LungsInt.Hot) - (lambdaPhys*LungsInt.Hot)
176	d(LungsInt.Cold)/dt = (PS_Lungs/LungsVas*LungsVas.Cold-PS_Lungs/LungsInt*LungsInt.Cold) + (lambdaPhys*LungsInt.Hot)
177	d(AdiposeVas.Hot)/dt = -(lambdaPhys*AdiposeVas.Hot) - (k_on_toAlb*AdiposeVas.Hot*AdiposeVas.Albumin/AdiposeVas-k_off_toAlb*AdiposeVas.AlbHot) - (PS_Adipose/AdiposeVas*AdiposeVas.Hot-PS_Adipose/AdiposeInt*AdiposeInt.Hot) + (F_Adipose/Art*Art.Hot) - (F_Adipose/AdiposeVas*AdiposeVas.Hot)
	d(AdiposeVas.Cold)/dt = (lambdaPhys*AdiposeVas.Hot) - (k_on_toAlb*AdiposeVas.Cold*AdiposeVas.Albumin/AdiposeVas-

178	k_off_toAlb*AdiposeVas.AlbCold) - (PS_Adipose/AdiposeVas*AdiposeVas.Cold-PS_Adipose/AdiposeInt*AdiposeInt.Cold) + (F_Adipose/Art*Art.Cold) - (F_Adipose/AdiposeVas*AdiposeVas.Cold)
179	d(AdiposeVas.AlbHot)/dt = (k_on_toAlb*AdiposeVas.Hot*AdiposeVas.Albumin/AdiposeVas-k_off_toAlb*AdiposeVas.AlbHot) - (lambdaPhys*AdiposeVas.AlbHot) + (F_Adipose/Art*Art.AlbHot) - (F_Adipose/AdiposeVas*AdiposeVas.AlbHot)
180	d(AdiposeVas.AlbCold)/dt = (lambdaPhys*AdiposeVas.AlbHot) + (k_on_toAlb*AdiposeVas.Cold*AdiposeVas.Albumin/AdiposeVas-k_off_toAlb*AdiposeVas.AlbCold) + (F_Adipose/Art*Art.AlbCold) - (F_Adipose/AdiposeVas*AdiposeVas.AlbCold)
181	d(AdiposeVas.Albumin)/dt = -(k_on_toAlb*AdiposeVas.Hot*AdiposeVas.Albumin/AdiposeVas-k_off_toAlb*AdiposeVas.AlbHot) - (k_on_toAlb*AdiposeVas.Cold*AdiposeVas.Albumin/AdiposeVas-k_off_toAlb*AdiposeVas.AlbCold) + (F_Adipose/Art*Art.Albumin) - (F_Adipose/AdiposeVas*AdiposeVas.Albumin)
182	d(AdiposeInt.Hot)/dt = (PS_Adipose/AdiposeVas*AdiposeVas.Hot-PS_Adipose/AdiposeInt*AdiposeInt.Hot) - (lambdaPhys*AdiposeInt.Hot)
183	d(AdiposeInt.Cold)/dt = (PS_Adipose/AdiposeVas*AdiposeVas.Cold-PS_Adipose/AdiposeInt*AdiposeInt.Cold) + (lambdaPhys*AdiposeInt.Hot)

References

[1] Chandra, R., Rahmim, A.: Nuclear Medicine Physics: the Basics. Wolters Kluwer, Philadelphia (2017)

[2] Kletting, P., Schuchardt, C., Kulkarni, H.R., Shahinfar, M., Singh, A., Glatting, G., Baum, R.P., Beer, A.J.: Investigating the effect of ligand amount and injected therapeutic activity: A simulation study for 177Lu-Labeled PSMA-Targeting peptides. PLoS One **11**(9), 0162303 (2016)
